# Chromatin loops are an ancestral hallmark of the animal regulatory genome

**DOI:** 10.1038/s41586-025-08960-w

**Published:** 2025-05-07

**Authors:** Iana V. Kim, Cristina Navarrete, Xavier Grau-Bové, Marta Iglesias, Anamaria Elek, Grygoriy Zolotarov, Nikolai S. Bykov, Sean A. Montgomery, Ewa Ksiezopolska, Didac Cañas-Armenteros, Joan J. Soto-Angel, Sally P. Leys, Pawel Burkhardt, Hiroshi Suga, Alex de Mendoza, Marc A. Marti-Renom, Arnau Sebé-Pedrós

**Affiliations:** 1 Centre for Genomic Regulation (CRG), Barcelona Institute of Science and Technology (BIST), Barcelona, Spain; 2 Centre Nacional d’Anàlisis Genòmic (CNAG), Barcelona, Spain; 3 Universitat Pompeu Fabra (UPF), Barcelona, Spain; 4 Michael Sars Centre, University of Bergen, Bergen, Norway; 5 Department of Biological Sciences, University of Alberta, Edmonton, Alberta Canada; 6 Department of Life and Environmental Sciences, Faculty of Bioresource Sciences, Prefectural University of Hiroshima, Shobara, Japan; 7 School of Biological and Behavioral Sciences, Queen Mary University of London, London, UK; 8 ICREA, Barcelona, Spain; 9 Wellcome Sanger Institute, Wellcome Genome Campus, Cambridge, UK

**Keywords:** Evolution, Gene regulation, Chromatin structure, Comparative genomics

## Abstract

In bilaterian animals, gene regulation is shaped by a combination of linear and spatial regulatory information. Regulatory elements along the genome are integrated into gene regulatory landscapes through chromatin compartmentalization^1,2^, insulation of neighbouring genomic regions^3,4^ and chromatin looping that brings together distal *cis*-regulatory sequences^5^. However, the evolution of these regulatory features is unknown because the three-dimensional genome architecture of most animal lineages remains unexplored^6,7^. To trace the evolutionary origins of animal genome regulation, here we characterized the physical organization of the genome in non-bilaterian animals (sponges, ctenophores, placozoans and cnidarians)^8,9^ and their closest unicellular relatives (ichthyosporeans, filastereans and choanoflagellates)^10^ by combining high-resolution chromosome conformation capture^11,12^ with epigenomic marks and gene expression data. Our comparative analysis showed that chromatin looping is a conserved feature of genome architecture in ctenophores, placozoans and cnidarians. These sequence-determined distal contacts involve both promoter–enhancer and promoter–promoter interactions. By contrast, chromatin loops are absent in the unicellular relatives of animals. Our findings indicate that spatial genome regulation emerged early in animal evolution. This evolutionary innovation introduced regulatory complexity, ultimately facilitating the diversification of animal developmental programmes and cell type repertoires.

## Abstract (web summary)

The physical organization of the genome in non-bilaterian animals and their closest unicellular relatives is characterized; comparative analysis shows chromatin looping is a conserved feature of genome architecture and spatial genome regulation emerged early in animal evolution.

## Main

A fundamental characteristic of animal multicellularity is the existence of specialized cell types. These cell types result from differential access to genomic information in each cell. Thus, evolutionary changes in genome regulation are proposed to be a major innovation linked to the emergence of complex multicellularity with stable cell differentiation^6,10^. This idea is supported by comparative genomic analyses showing that gene innovation at the origin of animals was less extensive than previously thought^10,13^, thus suggesting that an important animal innovation was the ability to coregulate existing genes in different combinations.

In bilaterian animals, genome spatial compartmentalization mediates the organization of gene neighbourhoods that can be independently regulated^3,4,6^ and that are specific to different cell types^14^. Another mechanism contributing to elaborate gene regulation in bilaterians is the combinatorial interaction of distal *cis-*regulatory elements and gene promoters by means of chromatin loops that bring distant regions into spatial proximity through genome folding, contrasting with the predominant regulation by proximal promoter elements in unicellular eukaryotes^10^. Comparative analyses of histone posttranslational modifications have shown that candidate distal enhancer elements, as defined by chromatin features, predate the origin of bilaterian animals^8,15,16^, whereas such enhancers are absent in the closest unicellular relatives of animals^17^. However, it is still unclear whether distal regulation in early-branching metazoans is mediated by physical interactions with gene promoters or linked to the existence of insulated gene regulatory landscapes.

**Fig. 1:**
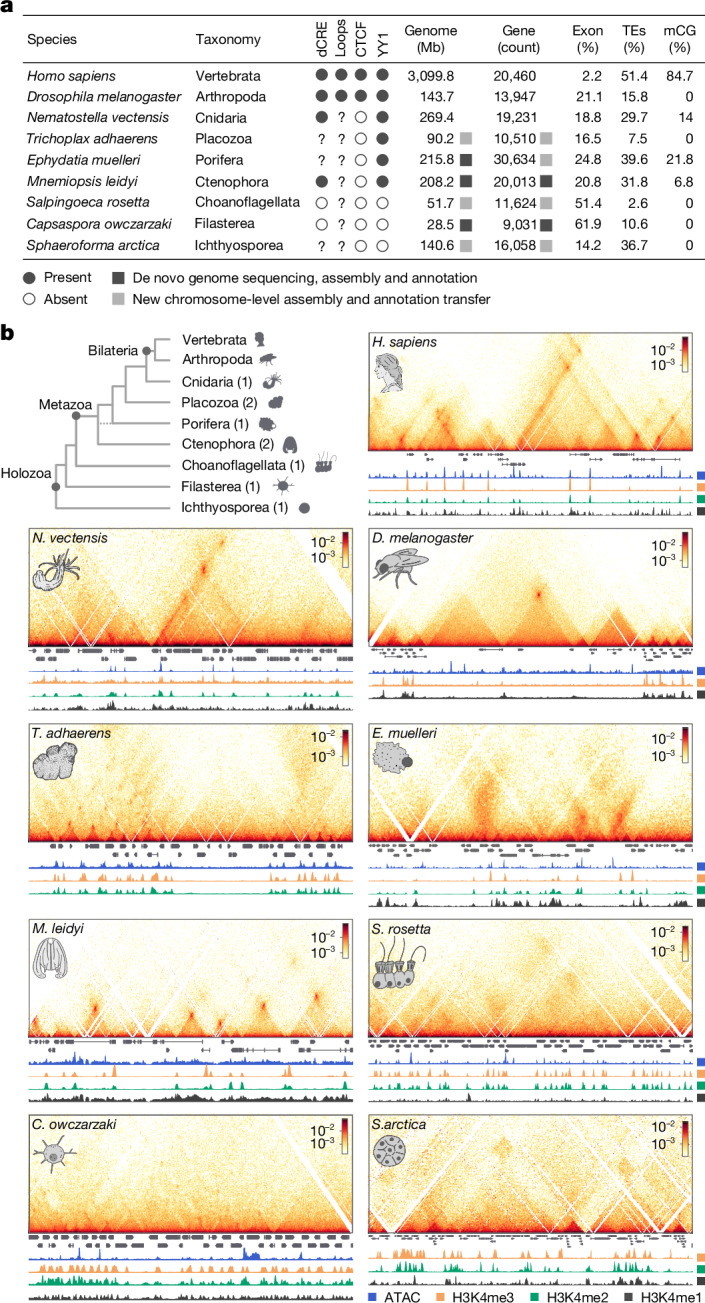
Chromatin architecture in early animal evolution. **a**, Comparison of genomic features across metazoans and unicellular holozoans. For *H. sapiens*, we used previously published mCG methylation percentage data from H1 ESCs cells. Of note, although distal *cis*-regulatory elements (dCRE) were identified in *Amphimedon queenslandica*^15^, their presence in *E. muelleri* had not been reported previously. mCG, CG methylation; TEs, transposable elements. **b**, Top left, phylogenetic tree showing the taxon sampling in this study, along with the number of profiled species per clade. Top right and below, Micro-C interaction maps of specific genomic regions (*S. arctica*, chr. 2: 3400000–3700000, bin 1 kb; *C. owczarzaki*, chr. 01: 3660000–3800000, bin 400 bp; *S. rosetta*, chr. 21: 800000–1100000, bin 800 bp; *M. leidyi*, chr. 8: 15500000–15700000, bin 400 bp; *E. muelleri*, Emue22: 2200000–2400000, bin 800 bp; *T. adhaerens*, TadhH1_4: 3880000–4180000, bin 800 bp; *N. vectensis*, NC_064040.1: 11650000–12000000, bin 1 kb; *D. melanogaster*, chr. 3L: 20480000–20820000, bin 800 bp; and *H. sapiens*, chr. 12: 69000000–71000000, bin 5 kb), showing examples of insulation boundaries or chromatin loops. All interaction maps were balanced using ICE normalization.

To investigate the origins of animal gene regulation, here we comparatively studied chromatin architecture at subkilobase resolution in non-bilaterian animal lineages and their closest unicellular relatives of animals (Fig. 1). This includes the two phyla proposed as the sister group to all other animals^9,18^: ctenophores, which are mostly pelagic, marine predators that swim using ciliated comb cells and have complex nerve nets^19,20^; and sponges, which are sessile, benthic organisms that filter-feed using collared choanocyte cells^8,21^. We also examined placozoans, which are millimetre-sized, flat animals that feed on microbial mats by gliding using ciliary movement and mucus secretion, controlled by peptidergic secretory cells^22^, and cnidarians, the sister group to bilaterians that includes jellyfishes, corals and anemones^23^. Finally, we studied three unicellular relatives of animals, known as unicellular holozoans: ichthyosporeans, which are osmotrophic unicellular eukaryotes that reproduce through multinucleated coenocytes^24^; filastereans, which are heterotrophic protists with complex life cycles, including aggregative multicellular stages^17,25^ and choanoflagellates, which are heterotrophic flagellates that show both single-cell and colonial forms and are the closest living relatives to animals^26^. The comparative analysis of chromatin maps across these lineages allows us to reconstruct the evolutionary history of genome regulation in animals.

## Large-scale genome organization

**Extended Data Fig. 1:**
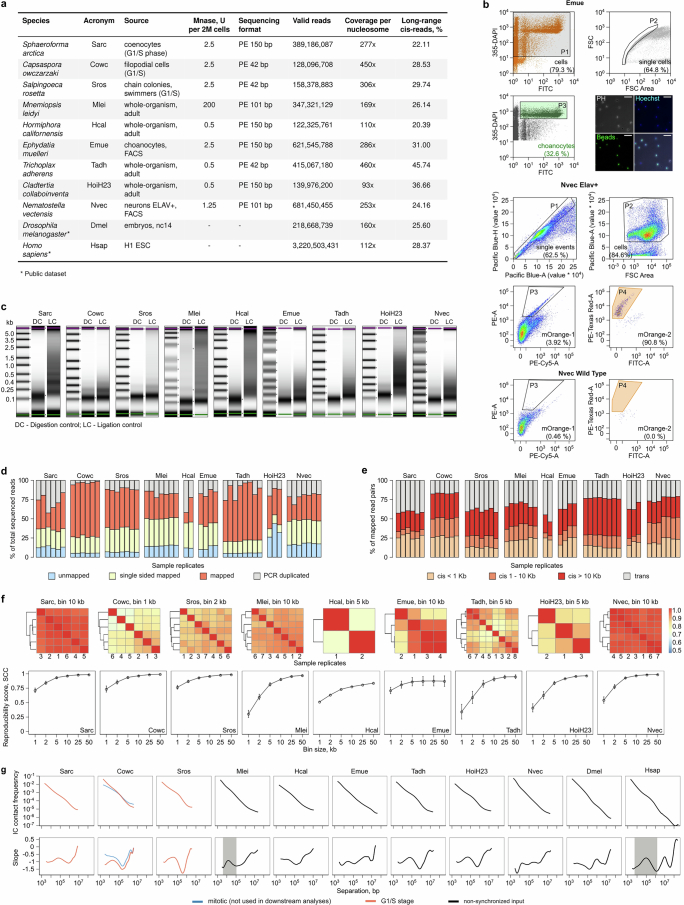
Micro-C experimental design and quality metrics. **a**, Overview of the input material for Micro-C experiments, library preparation strategy, and sequencing statistics in each species. The *D. melanogaster* dataset^27^ was subsampled to match the coverage of generated Micro-C maps in this study. **b**, Top, fluorescence-activated cell sorting (FACS) profile of crosslinked phagocytic choanocytes from *E. muelleri* labelled by feeding sponges with fluorescent microspheres. Only cells positive for nuclei Hoechst 33342 staining together with fluorescent beads were sorted. The sorted cell population (P3) was selected using sequential gating strategy through P1 and P2. Right, fluorescent microscopy image of sorted choanocytes, where PH stands for phase contrast, nuclei are in blue, FluoSpheres beads are in green. Scale bar (top right corner) is 50 µm. Below, the sequential FACS gating strategy (P1 - P3) to sort mOrange::NvElav+ neuronal cells (P4) from the *N. vectensis* transgenic line^65^. Wild type animals lacking the mOrange fluorescent protein were used to verify the gating strategy. The FACS sorting experiment data for *E. muelleri* and *N. vectensis* are representative of at least 6 independent experiments. **c**, The quality of chromatin digestion with MNase and followed proximity ligation was assessed with High sensitivity D5000 ScreenTapes using the Agilent 2200 TapeStation systems. The optimal chromatin fragmentation with Mnase results in up to 80% mononucleosomes profile. **d**, Barplots showing the percentage of reads mapped to the genome of each species. **e**, Barplots illustrating the distibution of intrachromosomal (*cis*) and interchromosomal (*trans*) interactions in each replicate experiment. The percentage of trans-contacts observed is species-specific but can be influenced by several factors: (i) the type of nuclear organization, such as Rabl-like configuration or the presence of chromosome territories, (ii) a high chromosome count, as seen in *S. arctica* and *S. rosetta*, (iii) and the reduction in nuclear diameter during the growth of coenocytes in *S. arctica*. **f**, Heatmap showing pairwise similarity scores between biological and technical replicates calculated as the stratum adjusted correlation coefficient (SCC). Below, SCC scores were estimated for a range of resolutions of 1 Kb, 2 Kb, 5 Kb, 10 Kb, 25 Kb, and 50 Kb. Differences in pairwise comparisons between experimental replicates are shown as mean ± s.d. The number of replicates per species as in (d-e). **g**, Top, *cis*-decay plots showing the rate of decay of contact frequency over genomic distance. The contact probability is averaged over all chromosomes. For *C. owczarzaki*, samples obtained from mitotic (blue) and synchronised G1/S stage (in orange) show different contact frequency behavior at short (below 10 Kb) and long (over 1 Mb) genomic distances. Bottom, log-derivative of cis-decay plots that predicts the folding of DNA into genomic structures and their size, most commonly chromatin loops, which tend to be the dominant micro-scale contacts. The first pronounced peak and dip at log-derivative cis-decay plots (highlighted in grey) in *M. leidyi* is observed at the scale from 10 Kb to 100 Kb. In *H. sapiens*, the peak size (highlighted in grey) ranges from 100 Kb to 1 Mb, which in both cases correspond to the average loop sizes in each species.

**Extended Data Fig. 2:**
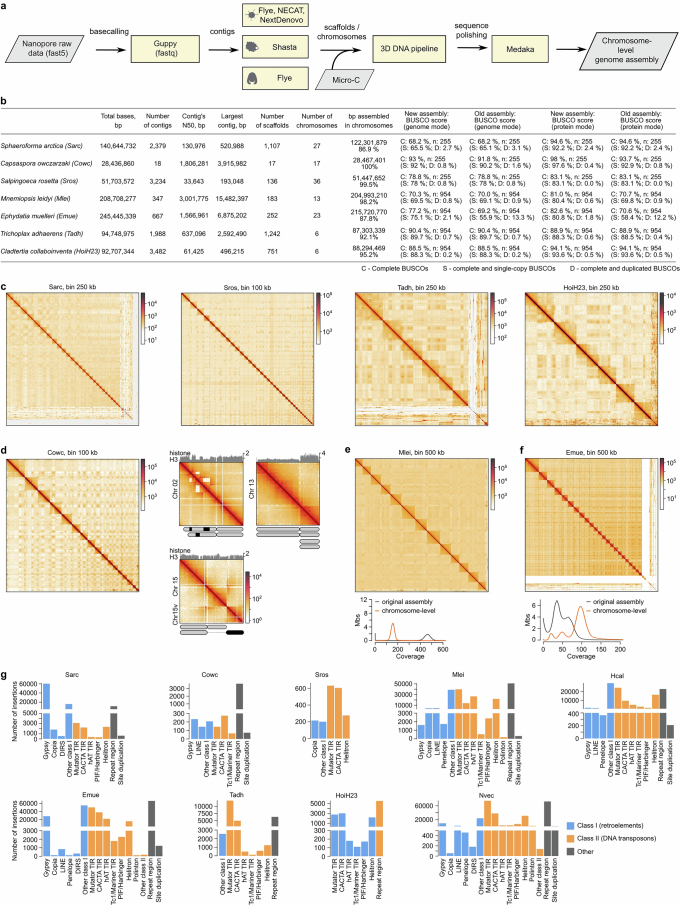
Genome sequencing, assembly and annotation. **a**, Genome assembly strategy. **b**, Genome assembly statistics. BUSCO completeness score was calculated using genome mode or protein mode against metazoan BUSCO dataset for all species except unicellular holozoans, where eukaryotic dataset was used. **c**, Chromosome-level re-assembly of *S. arctica*, *S. rosetta*, *T. adhaerens* and *C. collaboinventa* genomes using Micro-C data resulting in total of 27, 36, 6 and 6 chromosomes, respectively. Both *S. arctica* and *S. rosetta* posses genome-wide telomere clustering, whereas placozoans display strong interchromosomal compartmentalization signal. **d**, Left, genome-wide Micro-C contact map showing the chromosome-level assembly of *C. owczarzaki*. *C. owczarzaki* exhibit increased interactions between telomeres and between centromeres, suggesting Rabl-like chromosome configuration^138^. Right, chromosomal rearrangements in *C. owczarzaki*. Visual inspection of chromatin interaction maps revealed heterozygous deletions on *C. owczarzaki* chromosome 2, which is also confirmed by the uneven distribution of anti-H3 ChIP-seq coverage. In addition, one arm of the chromosome 13 exhibits genome-wide increase in the interaction frequency with other chromosomes, as well as two-fold coverage of H3 ChIP-seq, suggesting the gain of a chromosome arm pair. Finally, chromosome 15 v shares one arm with chromosome 15 and appears to be whole-arm translocation. **e**, Same as (d) for *M. leidyi*. The presence of uncollapsed haplotypes was estimated by distribution of per-base sequencing depth (left). Chromosomes in *M. leidyi* exhibit telomere clustering as well as increased intrachromosomal interactions similar to chromosome territories. **f**, Same as (d) for *E. muelleri* genome assembly with chromosome organisation similar to chromosome territories. **g**, Repeat content for each assembled genome, annotated using EDTA^97^.

We used Micro-C^11,12^ to map genome-wide chromatin contacts at single-nucleosome resolution in representatives of non-bilaterian animal lineages (Fig. 1, Extended Data Fig. 1, Supplementary Table 1 and Supplementary Text 1): the ctenophore *Mnemiopsis leidyi*^19,20^, the sponge *Ephydatia muelleri*^21^, the placozoan *Trichoplax adhaerens*^22^ and the cnidarian *Nematostella vectensis*^23^. As outgroup species, we studied chromatin architecture in three unicellular holozoans: the ichthyosporean *Sphaeroforma arctica*^24^, unicellular filasterean amoeba *Capsaspora owczarzaki*^17,25^ and the choanoflagellate *Salpingoeca rosetta*^26^. We also compared our chromatin maps with existing datasets from two bilaterians: *Drosophila melanogaster*^27^ and *Homo sapiens*^12^. To analyse our chromatin contact experiments, we first resequenced de novo and assembled to chromosome-scale the genomes of *M. leidyi*, *E. muelleri* and *C. owczarzaki* using a combination of Nanopore (Oxford Nanopore Technology) long-read sequencing and Micro-C data (Extended Data Fig. 2). For *S. arctica*, *S. rosetta* and *T. adhaerens*, we rescaffolded existing genomes^22,24,26^ to chromosome level using Micro-C data. In addition, to interpret the observed contact features, we generated genome-wide maps of chromatin accessibility (assay for transposase-accessible chromatin with high-throughput sequencing or ATAC-seq), chromatin modifications (chromatin immunoprecipitation with sequencing (ChIP–seq) for H3K4me3, H3K4me2, H3K4me1) and gene expression (RNA sequencing or RNA-seq), or used published datasets when available (Supplementary Table 2). We integrated three-dimensional (3D) chromatin data with linear chromatin marks to systematically compare genome architectural features at different resolutions^3,4,7^ (compartmentalization, insulation and chromatin looping) and across phylogenetically distant species with diverse genome sizes, gene densities and transposable element content (Fig. 1a).

**Fig. 2:**
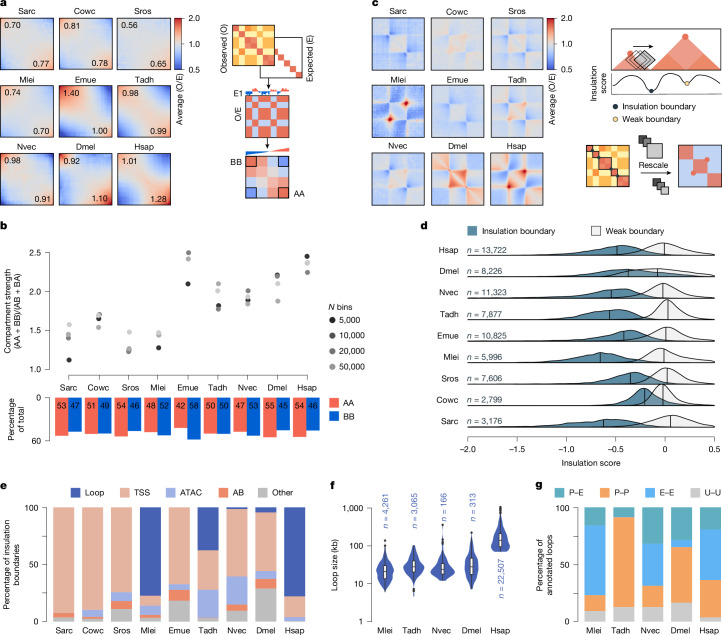
Chromatin compartments and insulation boundaries across species. **a**, Saddle plots showing contact interactions between A and B compartments in each species, organized by eigenvector ranking. To obtain the distance-normalized matrix, the ratio of observed-over expected interactions is calculated, followed by eigenvector decomposition. The eigenvectors are oriented and sorted from the lowest (B compartment) to the highest (A compartment) values. The bins of the interaction matrix then reordered according to the rank of the eigenvector. The observed (O) and expected (E) values are averaged to create a saddle plot. The top 20% of the interaction values were used to calculate the compartment strength values shown on the saddle plots. Cowc, *C. owczarzaki*; Dmel, *D. melanogaster*; Emue, *E. muelleri*; Hsap, *H. sapiens*; Mlei, *M. leidyi*; Nvec, *N. vectensis*; Sarc, *S. arctica*; Sros, *S. rosetta*; Tadh, *T. adhaerens*. **b**, Compartment strength quantification at different relative resolutions. The barplot below shows the contribution of homotypical chromatin interactions within active (AA) and inactive (BB) chromatin states. **c**, Aggregate plots showing contact enrichment within a rescaled region between two insulation boundaries. The boundaries are identified using the sliding diamond window to detect the changes in contact frequencies in each genomic bin. To plot pile-ups, regions between insulation boundaries are rescaled and their normalized observed and expected contact frequencies are averaged. **d**, Insulation score distributions illustrating the degree of isolation between linear genomic neighbourhoods. Number of annotated strong boundaries is indicated in blue, with a vertical line representing the median value of each distribution. **e**, Classification of insulation boundaries using hierarchical assignment of structural and genomic features. **f**, Size distribution of annotated chromatin loops in each species. The boxplots show the median (centre line), 25th and 75th percentiles (box limits) and the whiskers show the range of variability, excluding outliers, which are shown as individual points. **g**, Annotation of chromatin loop anchors with promoter (P) and enhancer (E) signatures based on normalized H3K4me3 and H3K4me2 or H3K4me1 ChIP–seq coverage. Chromatin loop anchors with undefined (U) epigenetic signature are shown in grey.

**Extended Data Fig. 3:**
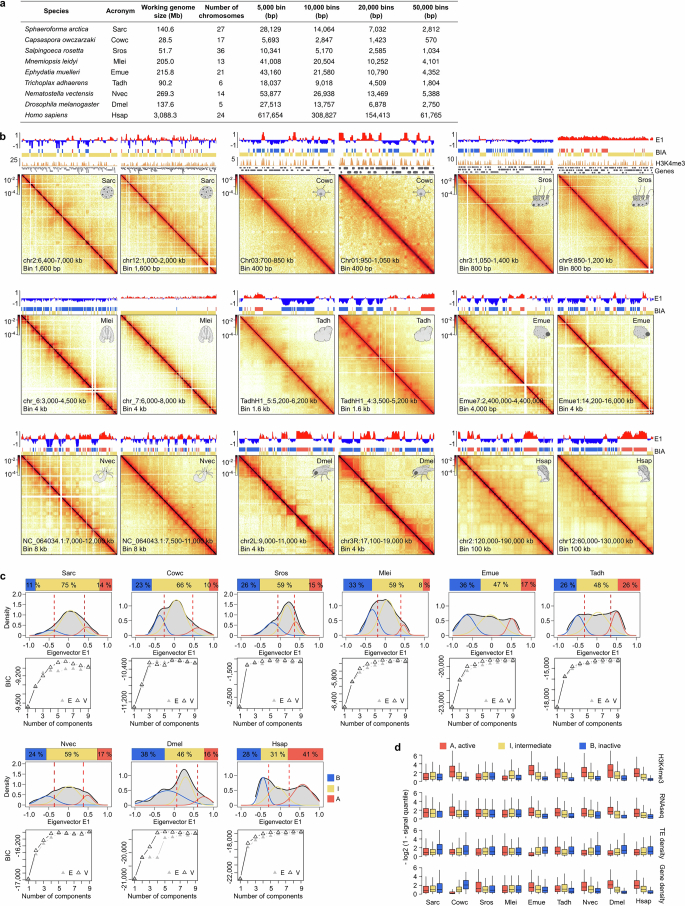
Genome compartmentalisation analysis. **a**, Table translating relative resolutions of contact maps that were used to calculate compartmentalisation signal into base-pair resolutions. **b**, Example genomic regions showing eigenvector coefficients E1 and compartment annotation into A (active, red), B (inactive, blue) and I (intermediate, yellow). **c**, Density plots showing genome-wide distribution of E1 eigenvalues and the relative abundance of each defined compartments (stacked barplot on top). Compartments were defined using fitting of Gaussian mixture with three components (k = 3). A Bayesian Information Criterion (BIC) was computed for the specified mixture (bottom plot) (see Method section). **d**, Association between chromatin compartments and different genomic features calculated per genomic bin at a relative resolution of 20,000 bins per genome, as in (a). The proportion of features in each compartment category follows the classification as in (c). The boxplots indicate the relative signal (measured as genome-wide quantiles) of the different features in the genomic bins belonging to each compartment category (active, intermediate, inactive). The mean value of distributions is shown as the center line on the boxplots, with interquartile range (IQR) as the box limits and whiskers extending to 1.5x IQR.

We first analysed global chromosomal compartmentalization, which results from the spatial segregation of distinct chromatin states genome-wide (active, A; inactive, B) and is influenced by histone marks, DNA methylation and gene transcription, among other phenomena^28,29^. As such, compartmentalization is often considered an intrinsic biophysical property of the chromatin driven by phase separation^30,31^. To compare the degree of self-affinity and segregation between major chromatin compartments, we defined A/B compartment limits in each species. We then calculated the intensity of compartmentalization in genomic bins with compartment A and B interaction frequency in the top 20th percentile (Fig. 2a). Compartmentalization strength in each species was quantified as the ratio of homotypic (AA, BB) to heterotypic (AB) interactions (Fig. 2b). The relative resolutions were obtained by partitioning genomes into equal number of bins across species (Extended Data Fig. 3a,b), but the differences between species remained consistent regardless of the number of bins used (Fig. 2b). Furthermore, we assigned an intermediate compartment (I) to regions with weak spatial separation (Extended Data Fig. 3c,d).

Our analysis revealed that, with exception of *M. leidyi*, animal genomes were globally segregated into transcriptionally active, gene-dense compartments and transcriptionally inactive, transposable element-rich compartments, similar to what is observed in bilaterian animals (Extended Data Fig. 3d). In these species, we detected a strong separation of A and B compartments in saddle plots (Fig. 2a) and the compartment strength values above 1.8 (Fig. 2b). Moreover, these compartments encompass relatively large contiguous regions across the genome (Extended Data Fig. 3b). By contrast, unicellular holozoans and *M. leidyi* did not show strong separation of large A and B compartments (Fig. 2a,b), similar to what is observed in yeast^32^ and other protists^33^. The absence of large-scale chromatin compartments in *M. leidyi* is unusual among animals, although it has been previously reported in certain species^34^. This lack of compartmentalization may be due to the absence of constitutively silenced regions across different cell types. Overall, our results indicate that A/B chromosomal compartmentalization is a phylogenetically conserved feature across animal genomes.

## Insulation and micro-scale contacts

**Extended Data Fig. 4:**
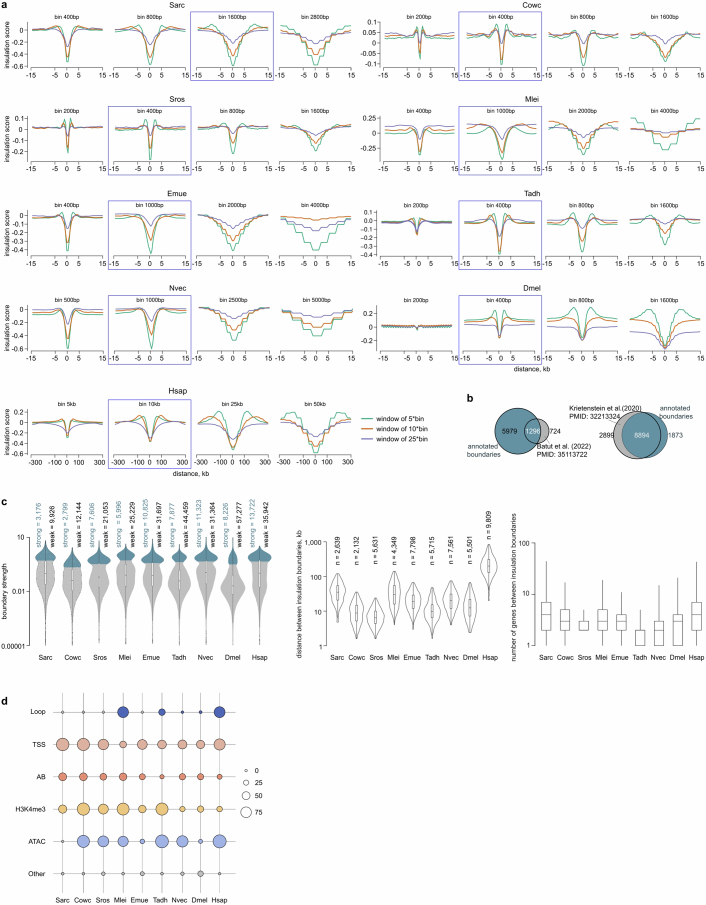
Genomic insulation and chromatin loop analysis. **a**, Insulation score profiles aligned at insulation boundary regions. Insulation score profiles were calculated for multiple resolutions with window sizes corresponding to 5x, 10x and 25x the chosen resolution. For example, for 400 bp resolution, we used window sizes of 2,000 bp (5*bin), 4,000 bp (10*bin), and 10,000 bp (25*bin). Parameters showing two strongest average insulation scores were considered optimal. For each of our studied species, an example contact map region with calculated insulation profile is shown. **b**, Left, overlap between regions annotated as strong boundaries using strategy described in this paper (see Method section) and previously published datasets^12,27^. **c**, Left, distribution of boundary strength values per species. Insulation boundaries (marked in blue) were selected using Li threshold as implemented in cooltools^103^. Middle, distribution of linear distances (Kb) between successive boundaries, with the number of examined region between boundaries indicated. Right, boxplots showing the number of genes located between insulation boundaries (same number of examined regions as in the previous plot). Boxplots center line shows the median value, with box limits indicating the IQR and whiskers as 1.5x IQR. **d**, Epigenetic, structural and gene features associated to insulation boundaries in each species. Note that a boundary can be annotated with multiple features (e.g. TSS, ATAC and H3K4me3 peaks).

We next characterized small-scale chromosomal features across species by defining spatial insulation boundaries between neighbouring loci. The boundary elements that partition genome into domains can arise from active transcription, silenced repetitive regions or binding of sequence-specific architectural proteins at insulator or tethering elements^5,27,35,36^. Thus, our first goal was to identify the occurrence of insulation boundaries in each species (Fig. 2c,d and Extended Data Fig. 4), and then classify these points into different regulatory or structural features (domain boundaries, gene bodies, regulatory loops and so on) (Fig. 2e). To this end, we calculated insulation scores for each species, representing the difference in contact frequencies between each genomic bin and its neighbouring bins. We used different resolutions and sliding window sizes (Extended Data Fig. 4a,b) and, for each species, we selected the resolution and two window sizes that yielded the maximal insulation signal, indicating the strongest partitioning of the genome into isolated structural and functional domains. The median distance between successive identified boundary elements varied between 6.4 kilobases (kb) in *S. rosetta* and 190 kb in *H. sapiens*, yet the median number of genes per interval was consistently similar across species, with two to four genes (Extended Data Fig. 4c).

The presence of self-interacting domains, contiguous regions of the genome with enriched interactions, was assessed by examining the average pile-up plots between insulation boundaries (Fig. 2c). We observed weak contact enrichment between pairs of insulated boundaries in unicellular holozoans and *E. muelleri*. In *M. leidyi*, boundary elements were tethered through strong focal contacts and without intradomain interactions, contrary to what would be expected within topologically associating domains (TADs)^3^. By contrast, *D. melanogaster* showed intradomain enrichment without focal contacts, in agreement with previously reported domains^37^. *T. adhaerens* and *N. vectensis* showed a certain degree of self-affinity within insulated neighbourhoods, as well as focal point enrichment (Fig. 2c). The degree of insulation of genomic regions could be quantified from the distribution of genome-wide insulation scores (Fig. 2d and Extended Data Fig. 4c). *M. leidyi*, *T. adhaerens*, *N. vectensis*, *H. sapiens* and *S. arctica* genomes contained strong boundary elements in comparison with *E. muelleri* and, especially, the weakly insulated genomes of *C. owczarzaki* and *S. rosetta* (Fig. 2d and Extended Data Fig. 4c).

After identifying insulation points, we investigated the genomic features associated with these boundaries (Fig. 2e and Extended Data Fig. 4d). We first assigned insulation boundaries to annotated chromatin loops, followed by the transcription start sites (TSSs) of genes not involved in chromatin looping and then accessible chromatin regions that may represent other regulatory elements. Remaining boundaries were assigned to A/B compartment limits. This analysis revealed that most insulation boundaries in unicellular holozoans and *E. muelleri* were associated with active TSSs (Fig. 2e), suggesting that active transcription is the main factor defining insulation in these species^37^. By contrast, many insulation boundaries could be assigned to chromatin loop anchors in *M. leidyi* (77%; compared to 78% in *H. sapiens* human embryonic stem cells) and in *T. adhaerens* (38%), whereas in *N. vectensis*, we identified 166 chromatin loops that represented only 1.6% of insulation boundaries. The number of chromatin loops in *M. leidyi* (4,261) and *T. adhaerens* (3,065) was much higher than those found in *N. vectensis* (166) and *D. melanogaster* (313)^27^, despite their similar genome sizes and gene densities (Fig. 1a). Loop sizes were comparable in these four species (median 21–28 kb), but much smaller than in *H. sapiens* (median 140 kb) with a genome 15–30 times larger (Fig. 2f). To further characterize these distal contacts, we examined genome-wide H3K4me3, H3K4me2 and H3K4me1 to classify many of the identified loop anchor sites as promoter-like elements (Fig. 2g). In *M. leidyi* and *N. vectensis*, chromatin loops predominantly occurred between promoters and enhancers (77 and 69%, respectively), similar to *H. sapiens* (63%). By contrast, 79% of loops in *T. adhaerens* connected promoters to other promoters, similarly to what is observed in *D. melanogaster* (49%)^38^. Our results show that enhancer–promoter and promoter–promoter long-range chromatin loops are shared between bilaterians and early-branching animal lineages, and possibly date back to the origin of animal multicellularity.

## Protists, sponges and cnidarians

**Extended Data Fig. 5:**
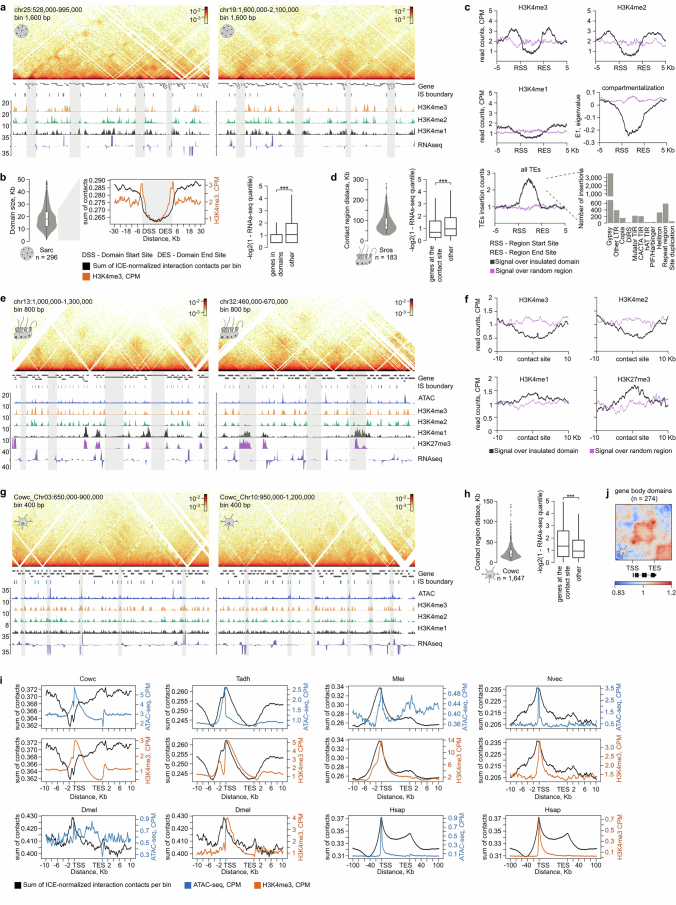
Genome architecture in unicellular holozoans. **a**, Example genomic regions in *S. arctica* illustrating the co-segregation of inactive chromatin regions. The interacting regions, highlighted in grey, fold into chromatin domains that exhibit local compartmentalised interactions. **b**, The manually annotated 296 compartment domains have a median size of 18 Kb. Middle, the observed long-distance interactions within the domains display a local checkerboard pattern, where contacts are enriched within certain set of loci, while contacts between them are depleted. To quantify the contact distribution, we calculated the sum of ICE (iterative correction and eigenvector decomposition)-normalized contacts within the segregated regions and their flanking regions (30 Kb) at a resolution of 2,800 bp, across the size range of 50 Kb to 5 Mb. The contact interaction pattern observed over the silenced regions showed a reduced interaction frequency across the region body compared to flanking loci. This interaction pattern is typical for checkerboard compartmentalisation, in contrast to loop interactions, which manifest as local peaks in interaction frequencies. Right, genes located within the compartment domains are lowly expressed or silenced (*** p-value < 2.2e^−16^, Wilcoxon rank sum test). Boxplots center line shows the median value, with box limits indicating the IQR and whiskers as 1.5x IQR. **c**, Distribution of epigenetic signals across compartment domains. The regions within the annotated domains were located within the inactive B compartment and were enriched in transposable elements, predominantly Gypsy LTRs, which accounted for 63% of the total TEs in these regions. **d**, Size distribution of manually annotated 183 contact regions in *S. rosetta* that harbour lowly expressed genes (*** p-value = 4.8e^−6^, Wilcoxon rank sum test), boxplots as in (b). **e**, Example genomic regions in *S. rosetta* forming distal interactions. **f**, Same as (c) for *S. rosetta*. The interacting regions show weak enrichment in H3K4me1 and H3K27me3 signals compared to random genomic regions. **g**, Same as (e) for *C. owczarzaki*. **h**, Distal contacts in *C. owczarzaki* connect promoter regions of highly expressed genes (*** p-value = 5.3e^−12^, Wilcoxon rank sum test), boxplots as in (b). **i**, Distal contacts in *C. owczarzaki* are indicative of micro-compartmentalisation signal because of the characteristic alternating contact pattern and the decreased cumulative interactions in the promoter regions of the target genes compared to the concentration typically seen in chromatin loops annotated in *T. adhaerens*, *M. leidyi*, *N. vectensis*, *D. melanogaster* and *H. sapiens*. To quantify the distribution of contact interactions around TSS-TES sites, we calculated and compared the sum of ICE (iterative correction and eigenvector decomposition)-normalized contacts at species-specific resolutions (400 bp for *C. owczarzaki, T. adhaerens* and *D. melanogaster*, 500 bp for *N. vectensis*, 800 bp for *M. leidyi* and 5 Kb for *H. sapiens*). To eliminate confounding signals from distal compartmentalisation pattern or other long-distance interaction patterns, the sum of considered interactions was restricted to contacts that fall within the range size of annotated loops or interacting regions (4–100 Kb for *C. owczarzaki, T. adhaerens*, 5–250 Kb for *D. melanogaster*, 10–360 Kb for *N. vectensis*, 5–150 Kb for *M. leidyi* and 50–1,060 Kb for *H. sapiens*). To calculate the average distribution of interaction contacts around the TSS-TES sites we used the function *stackup* form the *pybbi* package version 0.4.0 (https://github.com/nvictus/pybbi). The TSS-TES regions were rescaled into 50 bins with flanking regions of 10 Kb for each species except *H. sapiens* with 100 Kb flanking regions. Additionally, we flipped the TSS-TES regions and their corresponding flanking regions for negative-stranded genes. Notice that in *C. owczarzaki*, the sum of interactions around the TSS was lower than average interactions within the gene body. This is due to a small-scale local checkerboard pattern, where regions between interaction loci showed low contact frequency. As a result, cumulative interactions at promoters were even lower than average background signal and signal over gene bodies. In contrast, in other examined species, including *T. adhaerens*, *M. leidyi*, *N. vectensis*, *D. melanogaster* and *H. sapiens*, where chromatin loops connected examined promoter regions to cis-regulatory elements, the contact frequency at loop anchor regions was enriched and higher than the average across gene bodies. These differences highlight distinct modes of chromatin organization of *C. owczarzaki* with other species. **j**, In *C. owczarzaki*, a subset of highly expressed genes (274) exhibit increased interaction frequencies between TSS and TES forming gene body interaction domains.

In unicellular holozoans, we did not observe any spatial contact patterns indicative of chromatin loops. However, manual inspection revealed a few regions enriched in distal contacts. For example, in *S. arctica*, we could identify 296 self-interacting insulated domains that also contact each other (Extended Data Fig. 5a,b). These regions were depleted of active histone marks and were enriched in transposable elements, probably representing repressed chromatin domains that cosegregate (Extended Data Fig. 5c). In *S. rosetta*, there were 183 distally interacting regions that contained lowly expressed genes (Extended Data Fig. 5d,e) and were enriched in H3K4me1 and H3K27me3 or lacked profiled marks (Extended Data Fig. 5f). These may also represent repressed regions^39^, albeit they do not form well-defined domains like in *S. arctica*. In *C. owczarzaki*, we observed a plaid pattern indicative of chromatin microcompartments (Extended Data Fig. 5g), reflecting the spatial cosegregation of active promoters of highly transcribed genes with a strong H3K4me3 signal (Extended Data Fig. 5h,i). These microcompartment contacts form a regional small-scale checkerboard pattern with alternating loci of high and low interactions. Furthermore, we also detected high-frequency contact domains over gene bodies of highly expressed genes (Extended Data Fig. 5j).

**Extended Data Fig. 6:**
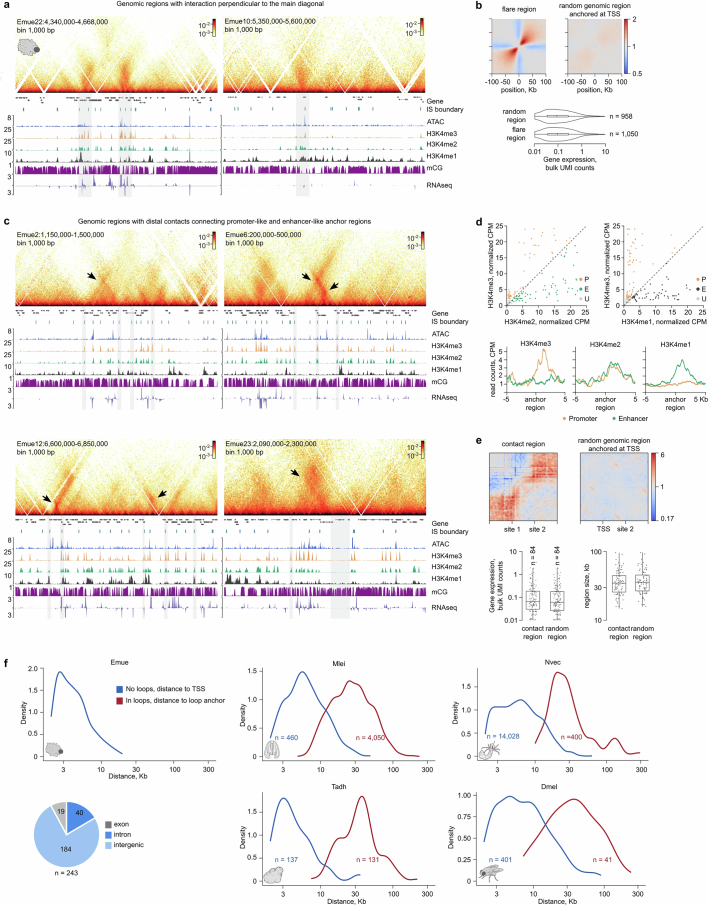
Genome architecture in the sponge *Ephydatia muelleri.* **a**, Example *E. muelleri* genomic regions showing contact patterns perpendicular to the diagonal of the Micro-C matrix and visually resembling flares^139^, jets^140^, or fountains^40,141^. **b**, Aggregated contact strength around the midpoints of flare regions. Random genomic regions anchored at the TSS of expression-matched genes were used as a control. Boxplots center line shows the median value, with box limits indicating the IQR and whiskers as 1.5x IQR. **c**, Example *E. muelleri* genomic regions showing distal interactions connecting promoter and enhancer-like anchor regions. Unlike typical chromatin loops, the preferential contact interactions in *E. muelleri* are diffuse and do not form a conspicuous dot contact pattern. **d**, A total of 84 manually annotated focal contacts connecting distal regulatory elements were classified as enhancers or promoters based on their H3K4me3 to H3K4me1 ratio. **e**, Aggregate plots demonstrating contact enrichment within rescaled contact regions, compared to random genomic regions anchored at TSS of expression-matched genes on one side and distance-matched random points on the other side. Boxplot limits are as in (b). **f**, Non-promoter cis-regulatory elements were identified based on chromatin state, defined by low H3K4me3 and high H3K4me1 enrichment around regions of accessible chromatin. The plots illustrate the distribution of these elements and their proximity to the nearest transcription start site (TSS) or other contact anchors within loop-forming enhancers. Notice the distance-to-TSS distribution of *E. muelleri* enhancer-like elements is similar to that of enhancers that do not form stable loops in other species.

In the sponge *E. muelleri*, we identified local interactions perpendicular to the main diagonal, and visually reminiscent to fountains observed in mouse, zebrafish and *C. elegans*^40^ (Extended Data Fig. 6a,b). Manual inspection further revealed 84 focal contacts between distal genomic loci (Extended Data Fig. 6c), including gene promoters interacting with other regions showing promoter or enhancer-like chromatin signatures (Extended Data Fig. 6d,e). These weak distal interactions occurred between extended genomic regions, in contrast to the point-to-point contacts typical of chromatin loops (Extended Data Fig. 6c). Although chromatin loops were absent in *E. muelleri*, we identified 243 distal *cis*-regulatory elements, consistent with findings in other sponge species^15^. These elements were characterized by chromatin accessibility, with surrounding regions showing high H3K4me1 and low H3K4me3 signals, and were mostly intergenic but close to annotated TSS (median 3.8 kb) (Extended Data Fig. 6f). This distance-to-TSS distribution was similar to that of annotated enhancer elements in *M. leidyi*, *T. adhaerens*, *N. vectensis* and *D. melanogaster* that do not form loops (median 5.6 kb, compared to 31 kb in loop-forming enhancers) (Extended Data Fig. 6f), suggesting that sponges’ enhancer elements might function by proximity without the need for stable looping^41^.

**Extended Data Fig. 7:**
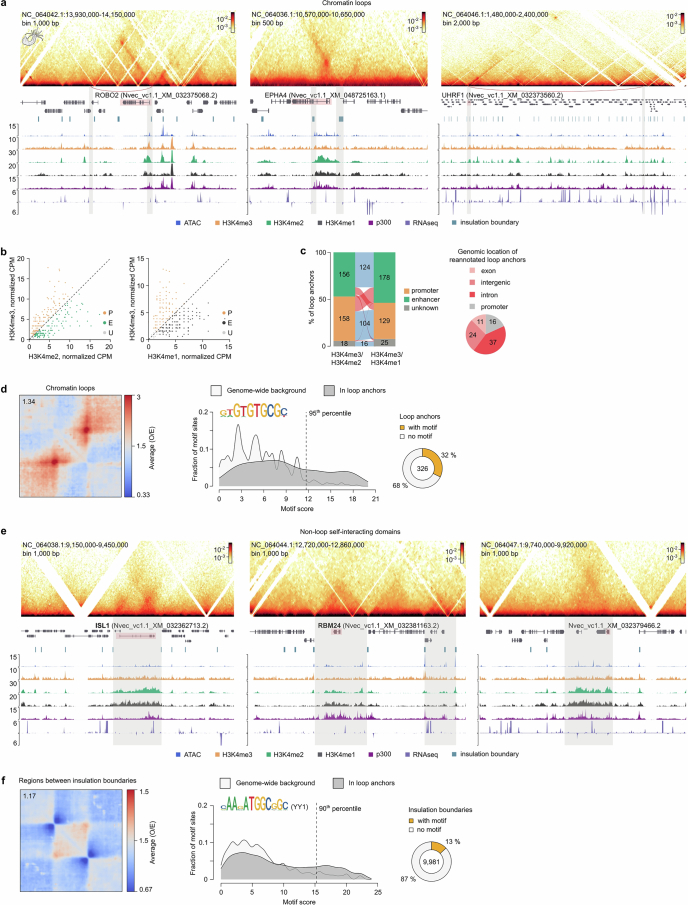
Genome architecture in the cnidarian *Nematostella vectensis.* **a**, Example genomic region in *N. vectensis* showing chromatin loop contacts with loop anchors highlighted in grey. **b**, Loop anchor regions were classified as promoter-side if characterized by high H3K4me3 ChIP-seq signal levels and low H3K4me2 or low H3K4me1 signal. Enhancer-side loop anchors were defined as regions with low H3K4me3 and high H3K4me2 or H3K4me1. **c**, Most loop anchors retained their original classification, regardless of whether the H3K4me3/H3K4me2 or H3K4me3/H3K4me1 ratio was used. For *N. vectensis*, the ratio of H3K4me3/H3K4me1 outperformed H3K4me3/H3K4me2 in classifying loop anchors, as most of the disputed loops anchors annotated as promoters with H3K4me3/H3K4me2 were predominantly located in intronic and intergenic regions (pie chart). **d**, Left, aggregated contact strength of chromatin loop interactions, showing the overall intensity and frequency of chromatin contacts across loop anchor points. Right, loop anchors in *N. vectensis* show GTGT-motif enrichment (FC = 327, p-value = 1e^−40^) compared to GC-normalised background genomic regions. **e**, Genomic regions in *N. vectensis* displaying non-loop self-interacting domains. **f**, Same as (d), but for regions between insulation boundaries that also harbour self-interacting domains. Right, motif enrichment analysis was focused on accessible chromatin regions at the insulation boundaries. Accessible promoter regions in neuronal Elav+ cells were used as the background for comparison.

Genome folding in the cnidarian *N. vectensis* was characterized by the presence of chromatin loops, as well as weakly insulated self-interacting domains (Extended Data Fig. 7). We identified 166 chromatin loops forming both promoter–promoter and promoter–enhancer contacts, and with some loops spanning nearly 1 megabase (Mb) (Extended Data Fig. 7a–c). Chromatin loops have also been reported in the hydrozoan *Hydra vulgaris*^42^, suggesting they are a conserved feature in cnidarians. Notably, some of the identified chromatin loops in *N. vectensis* showed a one-sided stripe pattern similar to those observed in other species, which are generated by cohesin extrusion^43^. Moreover, we identified an enriched GTGT motif (FC = 327, *P* = 1 × 10^−40^) present in 32% of loop anchors (Extended Data Fig. 7d). This motif resembles sequences with G-quadruplex-forming potential^44^, which have been shown to stabilize enhancer–promoter interactions in other species^45^. Beyond chromatin loops, we also observed self-interacting domains in *N. vectensis* (Extended Data Fig. 7e). The insulation boundaries of these domains were enriched for the YY1 motif (FC = 9,016, *P* = 1 × 10^−87^) (Extended Data Fig. 7f), which is known to mediate chromatin interactions^35,45^. These regions represent high-frequency contacts within the same gene regulatory landscape, but are not stabilized by chromatin loops as in vertebrate TADs^3^, nor are they as strongly insulated as the domains defined by insulator elements in *D. melanogaster*^27^.

## 3D promoter hubs in placozoans

**Fig. 3:**
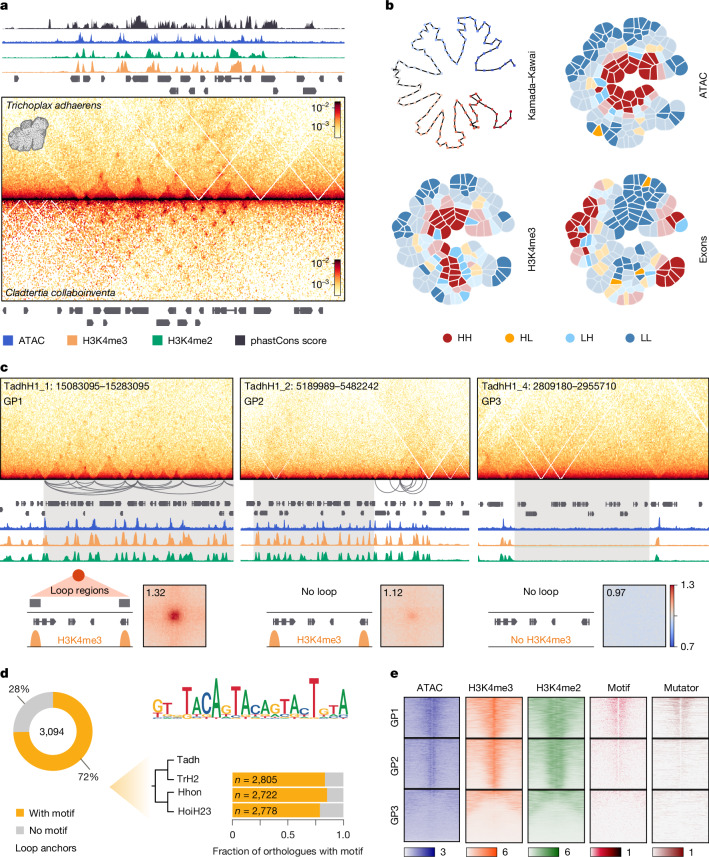
Promoter hubs in placozoans. **a**, Example of syntenic genomic regions in placozoans *T. adhaerens* (TadhH1_4: 3860000–4060000, bin 800 bp) and *C. collaboinventa* (chr. 4: 8983000–9183000, bin 800 bp). **b**, Gaudí plots projecting ATAC-seq, H3K4me3 and exon annotation signals onto a two-dimensional Kamada–Kawai graph layout (top left) represented by the top 20% of contact pairs with solid colours highlighting statistically significant regions (*P* < 0.05) identified using a one-sided permutation test. The high–high (HH) signal marks genomic bins enriched in signal and that are in spatial proximity with other bins enriched in signal; low–low (LL) bins are depleted in signal as well as neighbourhoods are depleted in signal; high–low (HL) and low–high (LH) are bins that are enriched in signal, but not their neighbourhood, and in reverse. **c**, Classification of *T. adhaerens* genes into three categories (GP1, GP2 and GP3) on the basis of structural and epigenetic features. Top, example regions containing genes classified into GP1, GP2 and GP3 groups. The resolution of Micro-C maps is 800 bp, maximum intensity value of ICE normalized Micro-C maps is as in **a**. Bottom, average loop strength between promoter regions of the genes from each groups is measured with APA. The colour bar of pile-up plots shows enrichment of observed over expected values. **d**, Sequence motif found in loop regions, which are also overlapping GP1 promoter regions (left panel), is present in promoter regions of orthologous GP1 genes in other placozoan species (right panel). The total number of shared orthologues is indicated. TrH2, *Trichoplax* sp. H2; Hhon, *Hoilungia hongkongensis*; HoiH23, *C. collaboinventa*. **e**, Heatmaps showing CPM normalized ATAC-seq and ChIP–seq coverage, motif scores and Mutator transposable element density ±5 kb around the TSS of GP1, GP2 and GP3 genes. Each heatmap scale starts at zero.

**Extended Data Fig. 8:**
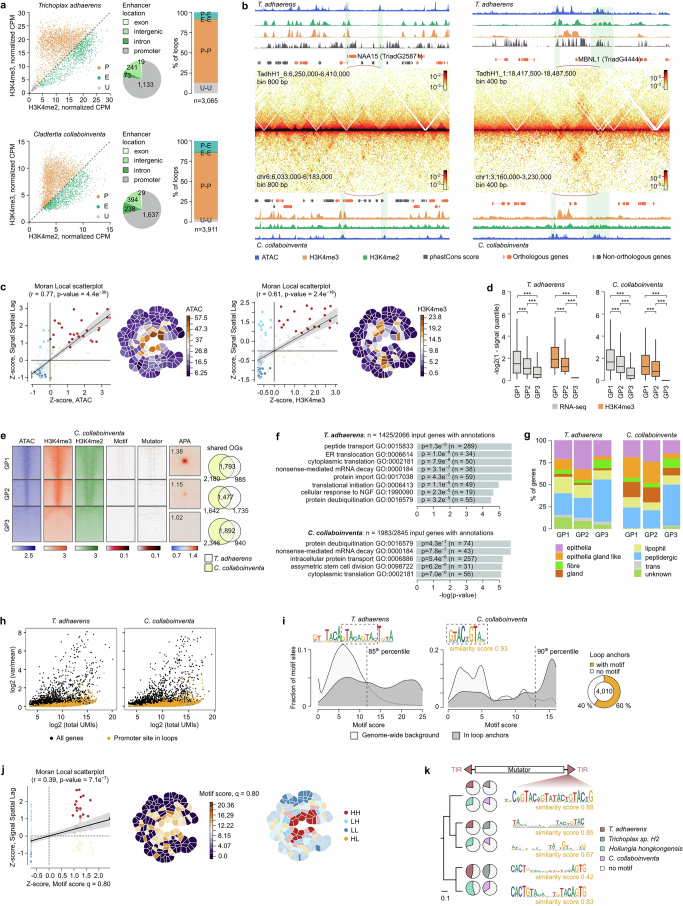
Placozoan genome architecture additional analyses. **a**, Annotation of chromatin loop anchors with promoter and enhancer chromatin signatures for *T. adhaerens* and *C. collaboinventa*. Loop anchors annotated as enhancers were mostly located within promoter regions of other genes. To resolve this ambiguity, such loop anchors were classified as promoters based on their genomic context. **b**, Example contact map regions depicting promoter-enhancer distal interactions highlighted in grey in syntenic regions of placozoans. **c**, Local Moran’s Index scatterplot visualises assignment of genomic bins to four distinct groups: High-High (HH), where examined signal (ATAC or H3K4me3) spatially co-localises in a neighbourhood of other bins with high signal; Low-Low (LL) bin has low examined signal and located in a neighbourhood of bins with low signal; when bin and its neighbourhood have different levels of signal, then the bin is assigned to Low-High (LH) or High-Low (HL) quadrants. Statistically significant values are in solid colors. Right panel illustrates intensity of examined signal layered over the two-dimensional Kamada-Kawai representation of top 20% contact interactions. p-values and r-values (Pearson correlation coefficients) were determined using a one-sided permutation test. A linear least-squares regression was then performed between z-scores of ATAC or H3K4me3 values and the signal’s spatial lag. The 95% confidence interval of the regression is shown as a grey shadow. **d**, Boxplots showing relative gene expression (RNA-seq) and peak intensity (H3K4me3) at promoter regions of genes from GP1, GP2, and GP3 groups. For each pairwise comparison for both *T. adhaerens* (GP1: n = 2,978; GP2: n = 3,681; GP3; n = 3,851) and *C. collaboinventa* (GP1: n = 3,973; GP2: n = 3,119; GP3: n = 4,238), *** indicates p-values below 2.22e^−16^, two-sided Wilcoxon rank sum test. Boxplots center line shows the median value, with box limits indicating the IQR and whiskers as 1.5x IQR. **e**, Left, heatmaps showing CPM normalised ATAC-seq and ChIP-seq coverage, motif scores and Mutator transposable element density within 5 Kb of the TSSs of GP1, GP2, and GP3 genes in *C. collaboinventa*. Each heatmap scale starts at zero. Middle, aggregate peak analysis displaying the contact strength between gene promoters within each annotated group. Right: Genes in *C. collaboinventa* from various gene groups, classified based on the presence of chromatin loops and their epigenetic states, demonstrate overlap with orthologous genes from GP1, GP2, and GP3 in *T. adhaerens*. **f**, GO-term enrichment analysis of GP1 genes with p-values determined using Fisher’s exact test. **g**, Barplots showing the cell type (from previously published dataset^123^) in which genes belonging to each group are maximally expressed. Only variable genes (with a fold-change higher than 1.8) are included. **h**, Scatterplot showing total gene expression (x-axis) versus gene expression variability (y-axis) across cell types. **i**, Distribution of motif scores in loop anchor regions compared to the genome-wide background. *C. collaboinventa* harbour similar motif to *T. adhaerens* (similarity score = 0.93) in 60% of annotated loop anchor regions. **j**, Local Moran’s Index scatterplot and Gaudí plots demonstrate spatial co-localisation of sequence motif identified in promoters of GP1 genes of *T. adhaerens* (motif score above 80^th^ percentile). Statistically significant values are calculated as in (c). **k**, Schematic phylogenetic tree of TIR sequences of Mutator DNA transposons from four placozoan species (*Trichoplax adhaerens*, *Trichoplax* sp. H2, *Hoilungia hongkongensis*, *Cladtertia collaboinventa*). Placozoan Mutator DNA TIRs can be classified into 5 clades with consensus sequences. The similarity score between the TIR consensus sequence and the sequence motif in GP1 promoters is indicated. Pie charts shows the proportion of Mutator transposons harbouring the consensus TIR sequences.

Our high-resolution chromatin contract maps revealed a complex 3D genome organization in the placozoan *T. adhaerens*, characterized by many loop contacts forming 3D interaction hubs (Fig. 3a). To confirm this observation, we profiled chromatin contacts in a distantly related placozoan species, *Cladtertia collaboinventa*, which showed a very similar pattern (Fig. 3a). Most of these interactions are promoter–promoter hubs (*n* = 2,413 for *T. adhaerens* and *n* = 3,239 for *C. collaboinventa*) (Extended Data Fig. 8a). Notably, 7–10% of chromatin contacts (*n* = 241 for *T. adhaerens*, *n* = 394 for *C. collaboinventa*) connected promoters with intronic or intergenic enhancer regions (Extended Data Fig. 8a,b), revealing the presence of distal *cis*-regulatory elements in placozoans.

We identified 321 promoter hub regions in the *T. adhaerens* genome and 331 in *C. collaboinventa*, involving 1,695 and 2,191 genes, respectively, with a median of four promoters in each hub in *T. adhaerens* and five in *C. collaboinventa*. To further reconstruct the 3D organization of these hubs, we used METALoci to calculate spatial correlation between genome folding and epigenetic (ATAC, H3K4me3 ChIP) or genomic (exon annotation) features (Fig. 3b and Extended Data Fig. 8c). This analysis revealed a nested structure where accessible promoter regions were central to the 3D interactions tightly clustered in space (ATAC-seq in Fig. 3b), whereas gene bodies and the first nucleosome (H3K4me3) occupied more peripheral locations (Fig. 3b). Furthermore, genes within spatial promoter hubs were linearly grouped along the genome, resembling the arrangement of housekeeping genes observed in mouse embryonic stem cells^46^. Alternatively, these structures could be associated with active transcription and the formation of micro-compartmentalized RNA polymerase II-driven transcription hotspots^47^.

Notably, not all collinear genes formed promoter hubs. Following this observation, we categorized genes into three groups based on their spatial and epigenetic organization (Fig. 3c and Extended Data Fig. 8d,e). This includes group 1 genes (GP1, *n* = 2,978 in *T. adhaerens*, *n* = 3,973 in *C. collaboinventa*) that had both ATAC and H3K4me3 peaks and formed chromatin loops, with an average interaction strength in aggregate peak analysis (APA) of 1.32, indicating the enrichment of Micro-C signal at loop anchors. Group 2 genes (GP2: *n* = 3,681 in *T. adhaerens*, *n* = 3,119 in *C. collaboinventa*) also showed ATAC and H3K4me3 peaks, but lacked strong distal contacts (APA = 1.12). Last, group 3 genes (GP3: *n* = 3,851 in *T. adhaerens*, *n* = 4,238 in *C. collaboinventa*) had neither chromatin loops (APA = 0.968) nor active chromatin marks (Fig. 3c). On average, GP1 genes showed a stronger H3K4me3 ChIP–seq signal and higher expression levels compared to genes in GP2 and GP3 (Extended Data Fig. 8d) and were associated with housekeeping functions, including intracellular trafficking, translation and messenger RNA processing (Extended Data Fig. 8f). By contrast, GP3 genes were enriched in cell type-specific functions related to peptidergic cells (Extended Data Fig. 8g,h), potentially explaining the lack of chromatin features in our bulk epigenomic experiments.

To understand what distinguishes placozoan GP1 genes, we analysed loop anchor sequences in both species using genomic sequences as background. We identified an enriched motif at chromatin loop anchor regions in both placozoan species (Fig. 3d and Extended Data Fig. 8i,j) and found that GP1 promoters frequently contained insertions of Mutator DNA transposable elements (Fig. 3e and Extended Data Fig. 8e), with the terminal inverted repeat (TIR) sequence of this transposon containing the identified sequence motif. To further explore this association, we constructed a phylogenetic tree including all intact Mutator TIR sequences in four placozoan species (Extended Data Fig. 8k and Supplementary Data 1). This analysis revealed a Mutator family shared across species and with consensus TIR sequences resembling the motif found in chromatin loops anchors (Extended Data Fig. 8k). The connection between chromatin loops and the Mutator transposable element suggests a potential evolutionary and functional relationship. One possibility is that an architectural protein in placozoans evolved to recognize the sequence motif within the Mutator TIRs, leading to ‘domestication’ of these sites as regulatory elements. Alternatively, the presence of the motif and Mutator TIR sequences may indicate targeted integration of Mutator transposons into promoter regions of highly expressed genes. Overall, our analyses showed that roughly one-third of *T. adhaerens* and *C. collaboinventa* genes are part of promoter hubs mediated by chromatin loops and that these contacts are associated with the presence of conserved Mutator DNA transposons harbouring a specific sequence motif.

## Enhancer–promoter loops in ctenophores

**Fig. 4:**
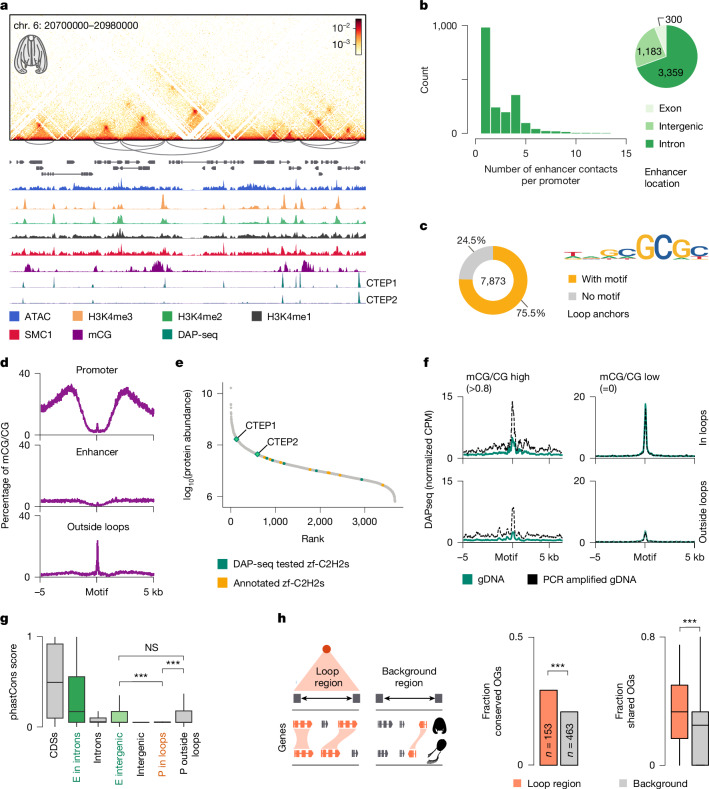
Chromatin loops in the ctenophore *M. leidyi.* **a**, Example genomic region showing chromatin loops between promoters and enhancers at 400 bp resolution. **b**, Left, histogram of enhancer contacts per promoter. Right, genomic location of enhancers. **c**, Sequence motif enriched in loop anchors. **d**, DNA methylation profiles centred around motifs located at promoter and enhancer loop regions, or outside loops. **e**, Chromatin-bound proteome of *M. leidyi*, showing identified proteins sorted by abundance with architectural proteins CTEP1 and CTEP2 as the most abundant zf-C2H2s. **f**, DAP-seq signals around GC-motif sites with high (left) versus low (right) methylation levels, and sites located within (top) or outside (bottom) of loop anchors. CTEP1 showed higher affinity for unmethylated GC-rich motifs in DAP-seq assays with native or PCR amplified gDNA (lacking methylation). **g**, Boxplots showing PhastCons conservation scores across three ctenophore species (*B. microptera*, *P. bachei* and *H. californensis*). The boxplot limits indicate the interquartile range (IQR), with the median as the middle line and whiskers extending to 1.5× IQR. Two-sided Wilcoxon rank sum test showed significant conservation differences between intergenic enhancers (*n* = 969) and promoters in loops (*n* = 778) (***P = 1.3 × 10^−15^) and between promoters in loops and promoters outside loops (*n* = 14,996) (***P < 2.22 × 10^−16^), whereas intergenic enhancers and promoters outside loops showed no significant difference (not significant (NS), *P* = 0.88). **h**, Syntenic conservation within *M. leidyi* chromatin loops compared to *H. californensis*. Left plot, barplot showing the fraction of conserved orthologues (OGs) in all alignable genomic regions across ctenophore species (***P *=* 5.5 × 10^−4^, chi-squared test for given probabilities). Right plot, boxplot of shared orthologues between individual genomic regions within chromatin loops (*n* = 115) versus in random genomic regions (*n* = 259) of similar size (***P = 2.4 × 10^−5^, Wilcoxon rank sum test with continuity correction). Boxplot limits as in **g**. Silhouette of *H. californensis* in **h** reproduced from PhyloPic (https://www.phylopic.org/), created by S. Haddock and K. Wothe under a CC0 1.0 Universal Public Domain licence.

**Extended Data Fig. 9:**
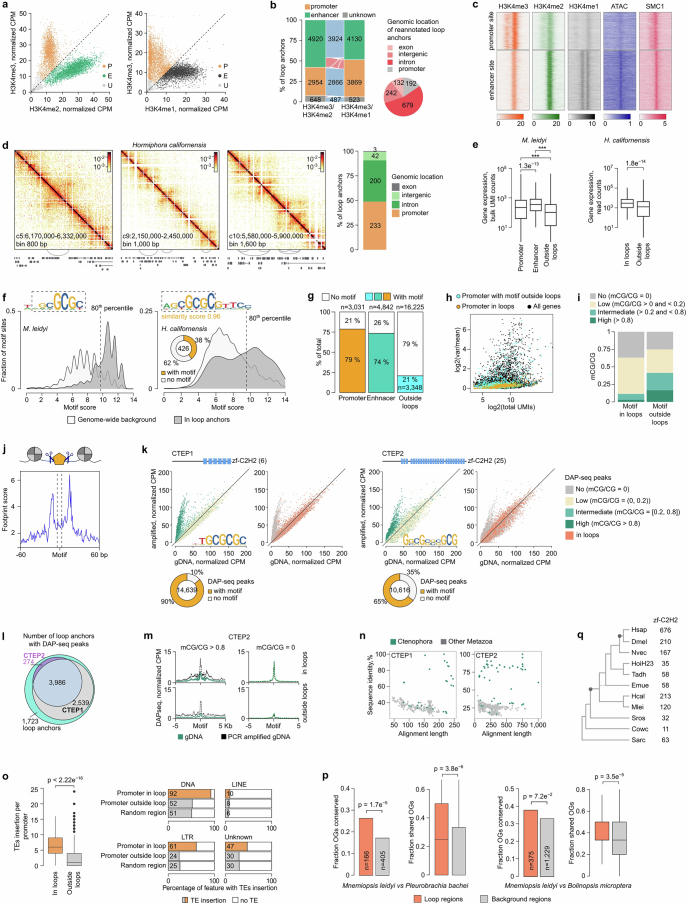
Ctenophore genome architecture additional analyses. **a**, Scatter plot showing the normalised H3K4me3 and H3K4me2 ChIP-seq coverage in 2 Kb region around loop anchor. **b**, Comparison of loop anchor annotation using either H3K4me3/H3K4me2 or H3K4me3/H3K4me1 ratios. For *M. leidyi*, H3K4me3/H3K4me2 ratio were more effective in annotating loop anchors, as many loop anchors classified as promoters using H3K4me3/H3K4me1 were found within intergenic or intronic regions (pie chart). The discrepancy is attributed to the high background noise observed in the H3K4me1 ChIP-seq signal. **c**, Normalised coverage for different chromatin features around loop anchors classified as promoters and enhancers. **d**, Genomic regions in *H. californensis* showing chromatin loops. In total, we annotated 239 chromatin loops, with 51% of loop anchors located within intronic or intergenic regions. High-resolution chromatin maps are expected to significantly increase the number of reported loops in *H. californensis*. **e**, Boxplots showing the total expression in scRNA-seq data^8^ for *M. leidyi* or RNA-seq data^51^ for *C. californensis* of genes with a loop anchor at their promoter regions, in their introns (enhancer sites), and genes not involved in distal chromatin interactions (outside loops). *** stands for p-value < 2.22e^−16^ of two-sided Wilcoxon rank sum test. Boxplots center line shows the median value, with box limits indicating the IQR and whiskers as 1.5x IQR. **f**, Motif score distributions at loop anchors (max score in 2,000 bp window around the center of a loop anchor) compared to genomic background. In *H. californensis*, we detected similar to *M. leidyi* GC-rich motif (similarity score = 0.96) enriched in 38% of loop anchors. **g**, Fraction of loop anchor sites containing the identified GC-rich motif at promoter sites (in orange), at enhancer sites (green) or at the promoters of genes not involved in chromatin loops (cyan). **h**, Scatterplot showing total gene expression (x-axis) versus gene expression variability (y-axis) across cell types, highlighting genes with their promoter involved in chromatin loops (orange) and also genes containing the GC-rich motif in their promoters but not involved in loops (cyan). These motif-containing genes without detected loops showed lower and more variable expression across cell types than genes with detected loops, suggesting the former could be forming loops in low-abundance cell types that we are unable to detect in bulk Micro-C experiments. **i**, DNA methylation levels at GC-motif sites located at chromatin loops (left) compared to methylation levels in motif occurrences outside detected chromatin loops (right). **j**, Bias-corrected ATAC footprint profiles centered around motifs located at loop anchors. **k**, Distribution of CTEP1 and CTEP2 bound DAP-seq peaks across genomic regions with varying DNA methylation levels and within annotated loop anchors. Below, the number of DAP-seq peaks containing the identified GC-rich motif. **l**, Number of loop anchor regions that contain CTEP1 and CTEP2 DAP-seq peaks. **m**, DAP-seq quantile normalized CPM coverage around GC-rich motif from CTEP2 binding assay using native genomic DNA fragments or unmethylated PCR amplified genomic DNA. CTEP2 as well as CTEP1 (Fig. 4f) exhibited higher affinity for the unmethylated GC-rich motif. **n**, Multiple sequence alignments of CTEP1 and CTEP2 genes were performed against the dataset of 358 metazoan genomes (Supplementary Table 3). The significant hits against CTEP proteins, exhibiting sequence identity above 50%, were found exclusively within ctenophores. **o**, Left, boxplots showing the number of transposable element insertions per promoter region of genes involved in chromatin loops compare to genes that are outside loops (*** indicates p-value < 2.22e^−16^, two-sided Wilcoxon rank sum test). Right, barplots showing the fraction of promoters in loops containing TE insertions compared to promoters not involved in loops and random genomic regions. Over 90% of promoter regions involved in distal interactions harbour insertion of DNA transposon. Additionally, promoters in loops have higher frequency of insertions of LTR and Unknown type transposons. Boxplot limits as in (e). **p**, Syntenic conservation within *M. leidyi* chromatin loops compared to *Pleurobrachia bachei* or *Bolinopsis microptera*. Left, barplot showing the fraction of conserved orthologs in all alignable genomic regions across ctenophore species (chi-squared test for given probabilities). Right, boxplot showing the fraction of shared orthologs between individual genomic regions within chromatin loops (*P. bachei*: n = 105; *B. microptera*: n = 332) versus in random genomic regions of similar size (*P. bachei*: n = 198; *B. microptera*: n = 945). p-value significance was calculated using two-sided Wilcoxon rank sum test. Boxplot limits as in (e). **q**, Number of predicted genes with zf-C2H2 protein domain in the different species studied included in this study.

The physical architecture of *M. leidyi* genome is dominated by thousands of chromatin loops (*n* = 4,261) (Fig. 4a), primarily connecting promoter and enhancer elements (61%), as well as enhancer to enhancer regions (16%) (Fig. 2g and Extended Data Fig. 9a,b). In total, we identified 916 gene promoters participating in chromatin loops, with each promoter contacting between one (50%) and up to 15 enhancers (Fig. 4b). These enhancers are mainly located in intronic (69%) and intergenic (24%) regions at one to eight genes from the contacted promoters. We also observed the accumulation of cohesin at loop anchor sites using ChIP–seq against SMC1 cohesin subunit (Extended Data Fig. 9c). To assess whether these features are conserved across ctenophores, we profiled chromatin contacts, albeit at lower resolution, in the cydippid ctenophore *Hormiphora californensis* (Extended Data Fig. 9d), which diverged from the lobate ctenophore *M. leidyi* roughly 180 million years ago^9^. At the sampled resolution, we detected 239 strong chromatin loops in *H. californensis*. In both ctenophores, genes involved in chromatin loop formation showed higher expression (Extended Data Fig. 9e).

To investigate whether the chromatin loops in ctenophores are formed in a sequence-specific manner, we searched for the enriched motif in loop anchors of both species, using GC-normalized genomic random sequences as a background. We identified GC-rich motif (FC = 8,522; *P* *=* 1 × 10^−497^) that was present in over 75% of loop anchors (Fig. 4c and Extended Data Fig. 9f) and at both promoter (79%) and enhancer sites (74%) involved in chromatin loops (Extended Data Fig. 9g). In addition, this motif was found in an extra 3,348 gene promoters (21% of all genes) with no chromatin loops detected (Extended Data Fig. 9g,h).

As the identified GC-rich motif contains two CpG dinucleotides, we examined DNA methylation using long-read Nanopore sequencing data. The overall methylation level in *M. leidyi* was low (6.8%), in agreement with previous reports using whole-genome bisulfite sequencing^48^. However, at loop anchor sites motifs showed low cytosine methylation, whereas motif occurrences outside loop anchor points showed high methylation (Fig. 4d and Extended Data Fig. 9i). Thus, we propose that DNA methylation of this GC-rich motif serves as a regulatory mechanism of loop formation in *M. leidyi*, potentially controlling the binding of an unknown, methylation-sensitive architectural DNA-binding factor, similar to mechanisms described for CCCTC-binding factor (CTCF) and other transcription factors^49^.

The presence of DNA-binding proteins was further supported by the ATAC-seq footprint profile at motif regions in loop anchors (Extended Data Fig. 9j). To identify these potential architectural proteins, we profiled the chromatin-bound proteome of *M. leidyi* (Fig. 4e). We then selected the most abundant zf-C2H2 domain-containing proteins and analysed their DNA-binding specificity using DAP-seq, as zf-C2H2 factors are often associated with chromatin looping in other species^2,5,35,50^. This analysis identified two proteins, named here CTEP1 (Ctenophore-specific Tethering Protein 1) and CTEP2, which overlapped with 80% of detected loop anchor regions and showed strong affinity for the same GC-rich motif we had previously identified (Extended Data Fig. 9k–m). Moreover, DAP-seq confirmed that the binding of both proteins was inhibited at sites with high DNA methylation (Fig. 4f and Extended Data Fig. 9k,m). Thus, we conclude that CTEP1 and CTEP2 bind unmethylated GC-rich motif sites at chromatin loops. Notably, these proteins are conserved across ctenophore species (Extended Data Fig. 9n and Supplementary Table 3), but are absent from genomes of other metazoans.

Finally, we analysed evolutionary conservation of the sequences at the loop anchor points. To this end, we calculated genome-wide conservation scores from alignments of *M. leidyi* genome with three other ctenophore species (*Bolinopsis microptera*^9^, *Pleurobrachia bachei*^20^ and *H. californensis*^51^). Chromatin loop anchors, both at intronic and intergenic regions, showed higher sequence conservation compared to other introns or random genomic regions, respectively (Fig. 4g). The promoters of genes involved in distal contacts showed lower conservation score compared to other promoters (Fig. 4g), with conservation levels similar to those of random intergenic regions. Moreover, these promoters had a high frequency of transposable element integrations and elevated DNA methylation (Fig. 4d and Extended Data Fig. 9o). Furthermore, we found that genes located within enhancer–promoter loop regions in *M. leidyi* have higher syntenic conservation across ctenophore species compared to other genomic regions of similar size (Fig. 4h and Extended Data Fig. 9p). Overall, the conservation of loop anchor regions across ctenophore species and the increased syntenic linkage of genes suggest that gene positioning is constrained by genome architecture. These findings indicate that the distal chromatin contacts identified in *M. leidyi* represent an evolutionary conserved mechanism of genome regulation present in both lobate and cydippid ctenophores.

## Discussion

Genome architecture is the result of both physicochemical and regulatory processes^3,4,31^. In unicellular organisms, chromatin contact patterns are shaped by the polymer nature of the chromatin fibre^32^ and by gene transcriptional states^52^. For example, gene body contact domains are observed in highly transcribed genes in *S. cerevisiae* and *S. pombe*^52^, and in *Arabidopsis thaliana*^53^. Also, insulation boundaries resulting from highly transcribed genes in divergent orientations are described in dinoflagellate genomes^33^. In unicellular holozoans, we observed similar insulation patterns around TSSs, but without evidence of further regulatory features or sequence-specific determinants associated with insulation boundaries. We also found cosegregating inactive chromatin regions in the large genome of *S. arctica*, and to a lesser extent in *S. rosetta*^39^. By contrast, these structures are absent in unicellular organisms such as *C. owczarzaki* or *S. cerevisiae*, which both have gene-dense genomes without heterochromatic regions.

In bilaterian species, extra chromatin structures involved in gene regulation have been observed, often mediated by architectural proteins binding to specific sequences^2,5,35,50^. These include discrete chromatin loops between *cis*-regulatory elements and promoters, mediated by tethering elements^27^, as well as insulated gene regulatory landscapes, such as loop TADs bounded by convergent CTCF sites in vertebrates^3^. Notably, TAD-like domain structures can also result from the passive cosegregation of active versus inactive chromatin states^37,54^, rather than being determined by sequence-specific insulation elements. Examples of these are Polycomb bodies^55^ and other heterochromatic compartment domains^28,29^. In early-branching animals we did not identify loop-bound TADs or any evidence of sequence-defined insulated TADs. However, we did detect chromatin loops spanning tens of kilobases and linking distal *cis*-regulatory elements and promoters in cnidarians, ctenophores and placozoans. In the case of ctenophores, thousands of chromatin loops link enhancers and promoters, showing that distal loops can be extremely frequent even in small genomes (roughly 200 Mb). Another example is the thousands of chromatin loops in placozoans, with even smaller genomes (roughly 100 Mb). Both placozoans and ctenophores complex looping architectures are associated with transposable elements. Although the causal relationship between transposable elements and chromatin loops is unclear, this observation suggests that complex 3D genome architectures might be influenced by lineage-specific transposable element invasion histories^56^.

The mechanisms and factors responsible for loop formation in non-bilaterians and most invertebrates remain unknown^7^. The zf-C2H2 protein CTCF is the main architectural protein in vertebrates and is conserved across bilaterians. In annelids it has been associated to open chromatin regions^57^ and in cephalopods it defines TAD boundaries^58^. Given that CTCF is absent in non-bilaterians^36^, other factors, possibly from the zf-C2H2 family (Extended Data Fig. 9q), might be involved in the formation of these loops. In fact, a variety of architectural proteins other than CTCF have been described in *Drosophila*, many of which are zf-C2H2 proteins with restricted phylogenetic distributions such as the insect-specific CP190 factor^2,50,59^. Similarly, we identified two ctenophore-specific zf-C2H2 proteins (CTEP1 and CTEP2) associated with loop anchor regions in *M. leidyi*. It is possible that other, yet unidentified, lineage-specific zf-C2H2 proteins contribute to chromatin architecture in different animal lineages.

**Fig. 5:**
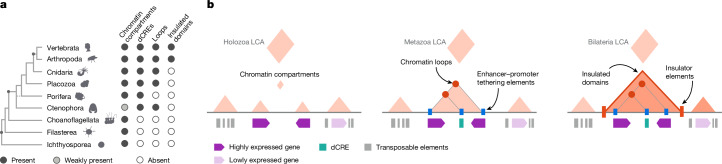
The evolution of animal regulatory genome architecture. **a**, Phylogenetic tree illustrating the taxonomic distribution of 3D-chromatin features. **b**, Schematic depicting major innovations in animal genome regulation at different ancestral nodes. LCA, last common ancestor.

Globally, our findings suggest an evolutionary scenario (Fig. 5) in which chromatin compartment domains defined by transcriptional states^28^ (but lacking sequence-specific insulation or tethering elements) were present in the unicellular ancestor of animals, as seen in extant unicellular holozoans. At the origin of animals, distal *cis*-regulatory elements evolved, requiring sequence-determined, stable chromatin looping mechanisms to link these enhancers with gene promoters (at least at certain distances^41^). This added an extra layer of regulatory complexity to cell type-specific gene regulation. The origin of this distal gene regulation would also explain the existence of regulatory-linked genomic regions showing conserved synteny^60^, as observed in ctenophore regions between loop anchor points. Moreover, domains insulated by sequence elements probably originated at the root of bilaterian animals, as they are observed in vertebrates, insects and probably spiralians^57,58^. In the specific case of vertebrates these domains are formed by a mechanism of CTCF-dependent loop extrusion so far not observed in any other lineage^7^, which further exemplifies the potential diversity of mechanisms involved in chromatin architecture across metazoans. Future extended taxon sampling will further refine this evolutionary scenario and help solve open questions such as whether there are conserved or lineage-specific factors involved in the establishment of chromatin loops across animals, how dynamic these structures are in development and across cell types or when did sequence-determined, insulated TADs first emerged in animal evolution.

## Methods

### Cell and animal cultures, sample preparation and crosslinking

*S. arctica* coenocytic culture was grown in marine broth (Difco, 3704 g l^−1^) at 12 °C in 25 cm^2^ flasks. Cells were passaged every 7 days using a 1:100 dilution. To synchronize cells in the G1/early S phase, an 8-day old culture was treated with 200 mM hydroxyurea (Sigma-Aldrich, catalogue no. H8627) for 18 h in the presence of 0.3% dimethylsulfoxide (DMSO). Synchronized cells were pelleted at 2,000*g* for 5 min at 12 °C, washed twice with Ca^2+^/Mg^2+^-free artificial sea water (CMFSW) (10 mM HEPES (pH 7.4), 450 mM NaCl, 9 mM KCl, 33 mM Na_2_SO_4_, 2.5 mM NaHCO_3_) and flash-frozen in liquid nitrogen. Frozen cells were then reconstituted in CMFSW and crosslinked with 1% formaldehyde (Thermo Scientific, catalogue no. 28906) for 10 min under vacuum. The crosslinking reaction was quenched with 128 mM glycine for 5 min in the vacuum desiccator, followed by a 15 min incubation on ice. Cells were pelleted at 4 °C for 10 min at 2,000*g*, washed once with CMFSW, reconstituted in CMFSW to the concentration of 2 M ml^−1^ and crosslinked with 3 mM DSG (Thermo Scientific, catalogue no. A35392) for 40 min at room temperature on a rotating wheel. The reaction was quenched with 400 mM glycine for 5 min. Double-crosslinked cells were pelleted at 4 °C for 15 min at 2,000*g* and flash-frozen in liquid nitrogen.

*C. owczarzaki* strain ATCC30864 was maintained in axenic culture at 23 °C in the ATCC (American Type Culture Collection) medium 1034 (modified PYNFH medium) in 25 cm^2^ flasks. For subculture, filopodial cells were passaged every 2–3 days using a dilution of 1:100. Before collection, filopodial cells were synchronized in G1 or early S phase by treating a filopodial culture of 70–80% confluency with 100 mM hydroxyurea (Sigma-Aldrich, catalogue no. H8627) for 18 h (ref. ^61^). Synchronized cells were scraped off the surface and pelleted at 2,200*g* for 5 min at room temperature. Collected cells were crosslinked as described in ref. ^62^. Briefly, cells were crosslinked with 1% formaldehyde (Thermo Scientific, catalogue no. 28906) in PBS for 10 min on a rotating wheel at room temperature. The crosslinking reaction was quenched with 128 mM glycine for 5 min at room temperature followed by extra incubation on ice for 15 min. The crosslinked cells were pelleted at 4 °C for 10 min at 2,000*g* and washed once with ice-cold PBS. Cells were diluted in PBS to the concentration of 2 M ml^−1^ and also crosslinked with 3 mM DSG (Thermo Scientific, catalogue no. A35392) for 40 min at room temperature on a rotating wheel. The crosslinking was quenched with 400 mM glycine for 5 min. Cells were pelleted at 4 °C for 15 min at 2,000*g* and flash-frozen in aliquots of 2 million cells.

*S. rosetta* was cocultured with *Echinicola pacifica* bacteria in artificial sea water supplemented with 20% cereal grass media (CGM3) at 23 °C. To synchronize the cell culture in the G1 or early S phase, cells from a 3-day-old culture were pelleted at 2,000*g* for 10 min and diluted in 4% CGM3 in artificial sea water to the concentration of 300,000 cells per ml. Cells were treated with 0.05 mM aphidicolin (Sigma-Aldrich, catalogue no. 178273) in the presence of 0.3% DMSO. After 18 h of incubation, cells, including chain colonies, fast and slow swimmers, were pelleted at 2,000*g* for 15 min. To remove bacteria from the choanoflagellate culture, collected cells reconstituted in 1 ml of culture media were passed through a Ficoll layer (1.6% Ficoll (Sigma-Aldrich, catalogue no. F5415), 0.5 M sorbitol, 50 mM Tris-HCl (pH 8.8), 15 mM MgCl_2_, 1% artificial sea water) by centrifugation at 1,000*g* for 10 min at 4 °C. Pelleted choanoflagellate cells were then double-crosslinked with 1% formaldehyde in CMFSW and 3 mM DSG in CMFSW as described above for *C. owczarzaki*. The crosslinked cells were pelleted at 4 °C for 15 min at 2,000*g* and flash-frozen in liquid nitrogen.

*E. muelleri* sponges gemmules were hatched and grown for 1 week in Strekal’s media^63^ in 150 × 25 mm culture dishes (Corning, catalogue no. 353025). To isolate phagocytic choanocyte cell population, specimens were fed for 10 min with 0.5 µm fluorescent carboxylate-modified FluoSpheres (Invitrogen, catalogue no. F8813) added to Strekal’s media to final 0.02% concentration (1:100 dilution of stock 2% FluoSpheres slurry)^64^. Sponges were washed once with Strekal’s media, and 1% formaldehyde solution in Strekal’s media was added to crosslink specimens for 10 min at room temperature with occasional mixing. To quench formaldehyde, 128 mM glycine was added and incubated for 5 min at room temperature and 15 min on ice. Crosslinked sponge specimens were washed twice with ice-cold Strekal’s media. Roughly 80 specimens were transferred in 5 ml of the Strekal’s media and dissociated by trituration until all tissue was removed from the gemmule husks (roughly ten trituration passages). The dissociated cell suspension was filtered through a 40-µm cell strainer, and cells were diluted to 2 M ml^−1^ concentration. The second crosslinking was performed with 3 mM DSG (Thermo Scientific, catalogue no. 20593) in Strekal’s media for 40 min at room temperature on a rotating wheel. The reaction was quenched with 400 mM glycine for 5 min at room temperature. Crosslinked cells were pelleted at 4 °C for 15 min at 2,000*g*, and then resuspended in 2 ml of ice-cold Strekal’s media with 2 µg ml^−1^ Hoechst 33342 (Thermo Scientific, catalogue no. 62249). Choanocytes were isolated using a BD FACS Aria II sorter with BD FACSDiva v.6.1.3 (BD Biosciences) as cells showing both FluoSphere fluorescence and Hoechst nuclei staining. Fluorescence-activated cell sorting (FACS) profiles were analysed with FlowJo v.10.7 (Extended Data Fig. 1b).

*M. leidyi* specimens were kept in 300-ml glass beakers with 5–10 individuals at 21 °C in artificial sea water (Red Sea, catalogue no. R11055) with a salinity of 27 ppt. Ctenophores were fed daily with a mixture of living rotifers (*Brachionus* sp.) and brine shrimps (*Artemia salina*). The water was exchanged once a week. For all experiments, adult lobate animals were starved for 2 days before collection. To dissociate animal tissue, roughly five adult animals (10 mm long) were transferred into CMFSW and washed twice to exchange the buffer. Animal tissue was dissociated into single cells in 5 ml of fresh CMFSW by triturating every 2 min for a total of 10 min. The efficiency of tissue dissociation was monitored under the microscope. Dissociated cells were filtered through a 40-µm cell strainer and diluted to 2 M ml^−1^ for the subsequent formaldehyde crosslinking. Cells were crosslinked in 1% formaldehyde in CMFSW for 10 min at room temperature. The reaction was stopped with 128 mM glycine for 5 min at room temperature and 15 min on ice. Crosslinked cells were pelleted at 4 °C for 10 min at 2,000*g*, washed once with CMFSW and resuspended to 2 M ml^−1^ for a second crosslinking with 3 mM DSG in CMFSW. The crosslinking reaction was stopped after 40 min of incubation at room temperature on a rotating wheel with 400 mM glycine for 5 min. The crosslinked cells were pelleted at 4 °C for 15 min at 2,000*g*.

*H. californensis* specimens from the first generation (F1) of a laboratory-reared culture at the Monterey Bay Aquarium (USA) were flash-frozen and pulverized in liquid nitrogen. Extracted cells and nuclei were filtered through a 40-µm cell strainer and pelleted by centrifugation at 4 °C for 10 min at 2,000*g*. Cells were double-crosslinked with 1% formaldehyde in CMFSW and 3 mM DSG in CMFSW as described for *M. leidyi*.

*T. adhaerens* and *C. collaboinventa* colonies were grown in 200 × 30 mm glass Petri dishes at 21 °C in artificial sea water (Red Sea, catalogue no. R11055) with a salinity of 33 ppt. Placozoans were fed once a week with unicellular algae (*Pyrenomonas* sp.), the water was exchanged every second week. To prepare single-cell suspension, roughly 500 animals were collected, washed twice with CMFSW and resuspended in 1 ml of CMFSW supplemented with 2 mM EDTA. Animal tissue was triturated every 2 min for a total of 10 min at room temperature. The efficiency of dissociation was monitored under the microscope. Dissociated cells were filtered through a 40-µm cell strainer, diluted to 2 M ml^−1^ and crosslinked with 1% formaldehyde in CMFSW for 10 min at room temperature on a rotating wheel. The reaction was quenched with 128 mM glycine for 5 min at room temperature and 15 min on ice. Cells were pelleted at 4 °C for 10 min at 2,000*g*, washed once with CMFSW and resuspended in 3 mM DSG in CMFSW for a second crosslinking. After 40 min of incubation at room temperature on a rotating wheel, 400 mM glycine was added to stop the reaction and cells were pelleted at 4 °C for 15 min at 2,000*g*.

*N. vectensis NvElav1::mOrange* transgenic line^65^ was maintained in one-third artificial sea water (Red Sea, catalogue no. R11055) with salinity of 14 ppt. To isolate *NvElav1::mOrange* positive cells, 1.5–2-month-old animals starved for 1 day before the experiment were crosslinked with 1% formaldehyde in Ca^2+^/Mg^2+^-free one-third sea water (one-third CMF: 17 mM HEPES (pH 7.4), 167 mM NaCl, 9 mM NaHCO_3_, 3.3 mM KCl) for 10 min under vacuum. The crosslinking reaction was stopped by adding 128 mM glycine and incubating the tissue under vacuum for 5 min, followed by a 15 min incubation on ice. The crosslinked tissue was dissociated into single cells by incubating the tissue with 10 mg ml^−1^ of Protease XIV (Sigma-Aldrich, catalogue no. P5147) in one-third CMF and 1 mM CaCl_2_ for 5 min at 24 °C triturating the tissue every 1 min. The digested tissue was pelleted at 800*g* for 5 min, reconstituted in one-third CMF supplemented with 2 mM EDTA and 2 µg ml^−1^ Hoechst 33342 (Thermo Scientific, catalogue no. 62249), and the trituration continued for another 5–10 min. Dissociated cells were filtered through a 40-µm cell strainer, and neurons were isolated using a BD FACS Aria II as cells showing both the mOrange signal and Hoechst nuclei staining (Extended Data Fig. 1b). Isolated *NvElav1::mOrange* positive cells were also crosslinked with 3 mM DSG for 40 min at room temperature.

### Micro-C library preparation

Micro-C libraries were prepared as previously described^11,12^ with the following modification. Double-crosslinked cells (2 million cells per sample) with 1% formaldehyde and 3 mM DSG were permeabilized with 500 µl of MB1 buffer (10 mM Tris-HCl (pH 7.4), 50 mM NaCl, 5 mM MgCl_2_, 1 mM CaCl_2_, 0.2% NP-40, protease inhibitor cocktail) for 20 min on ice with occasional trituration. Cells were pelleted at 4,500*g* for 5 min at 4 °C and washed once with MB1 buffer. To digest chromatin to a 80% monomers to 20% dimer and oligomers nucleosome ratio, an appropriate amount of MNase (Takara Bio, catalogue no. 2910a) was added (Extended Data Fig. 1a), and samples were incubated for 10 min at 37 °C with mixing at 850 rpm. The digestion reaction was stopped with 4 mM EGTA (pH 8.0) followed by incubation at 65 °C for 10 min without agitation. Cells were washed twice with ice-cold MB2 buffer (10 mM Tris-HCl (pH 7.4), 50 mM NaCl, 10 mM MgCl_2_, 0.1% BSA) and pelleted at 4,500*g* for 5 min at 4 °C. Next, to repair the fragment ends after MNase digestion, pelleted cells were resuspended in the repair reaction mix (5 µl of 10× NEBuffer 2.1, 34 µl of nuclease-free water, 1 µl of 100 mM ATP, 2.5 µl of 100 mM DTT) supplemented with 2.5 µl of 10 U µl^−1^ T4 PNK (NEB, catalogue no. M0201). After 15 min of incubation at 37 °C with 850 rpm agitation, 5 µl of 5 U µl^−1^ Klenow Fragment (NEB, catalogue no. M0210) was added to generate 3′–5′ overhangs in the absence of dNTPs for a subsequent incorporation of biotin-labelled dNTPs. The reaction mixture was incubated for another 15 min at 37 °C at 850 rpm. To biotinylate DNA fragment ends, the mixture of dNTPs was added to the reaction mix (2.5 µl of 10× T4 DNA Ligase buffer, 11.875 µl of nuclease-free water, 5 µl of 1 mM Biotin-dATP (Jena Bioscience, catalogue no. NU-835-BIO14), 5 µl of 1 mM Biotin-dCTP (Jena Bioscience, catalogue no. NU-809-BIOX), 0.5 µl of a mixture of 10 mM dTTP and dGTP, 0.125 µl of 20 mg ml^−1^ BSA). After 45 min of incubation at room temperature with interval mixing at 850 rpm, the reaction was stopped with 30 mM EDTA (pH 8.0) followed by incubation at 65 °C for 20 min without agitation. The chromatin from lysed cells and nuclei was pelleted at 10,000*g* for 10 min at 4 °C and washed twice with MB3 buffer (50 mM Tris-HCl (pH 7.5), 10 mM MgCl_2_). Finally, the chromatin was resuspended in 1,200 µl of proximity ligation mix (920 µl of nuclease-free water, 120 µl of 10× T4 DNA Ligase buffer, 100 µl of 10% Triton X-100, 12 µl of 20 mg ml^−1^ BSA, 36 µl of 50% PEG 4000, 12 µl of 5 U µl^−1^ T4 DNA ligase (Thermo Scientific, catalogue no. EL0012)) and incubated at room temperature for at least 2.5 h. To remove biotin from unligated ends, pelleted chromatin was treated with 2 µl of 100 U µl^−1^ Exonuclease III (NEB, catalogue no. M0206) for 5 min at 37 °C and agitation 850 rpm. Then, chromatin was decrosslinked and deproteinased overnight at 65 °C at 850 rpm in the presence of 350 mM NaCl, 1% SDS and 1 mg ml^−1^ proteinase K (Roche, catalogue no. 3115879001). The DNA was purified using DNA Clean & Concentrator-5 kit (Zymo Research, catalogue no. D4014) and eluted in 50 µl of 10 mM Tris-HCl (pH 8.0) (Extended Data Fig. 1c). Next, biotinylated proximity ligated DNA fragments were captured with Dynabeads MyOne Streptavidin (Life Technologies, catalogue no. 65602). DNA ends were prepared for adapter ligation and dA-tailed using NEBNext End repair/dA-tailing mix (NEB, catalogue no. E7546). The Y-shaped Illumina adapters were ligated with NEBNext Ultra II Ligation Module (NEB, catalogue no. E7595S), and the final library was amplified using NEBNext High-Fidelity 2× PCR Master Mix (NEB, catalogue no. M0541). The final libraries were double-size selected with Ampure XP (Beckman Coulter, catalogue no. A63881) resulting in libraries ranging from 350 to 750 bp in length. The detailed Micro-C stepwise protocol is reported in Supplementary Text 1.

### High molecular weight gDNA extraction for genome sequencing

Genomic DNA (gDNA) from *C. owczarzaki* (Cowc) strain ATCC30864 was extracted with Blood & Cell Culture DNA Mini Kit (Qiagen, catalogue no. 13323). The library was constructed by the use of Ligation Sequencing Kit (Oxford Nanopore, catalogue no. SQK-LSK109) and NEBNext Companion Module (NEB, catalogue no. E7180), and sequenced with the R9.4.1 Flow Cell set on a MinION device (Oxford Nanopore). We obtained 4.3 M reads with an estimated Oxford Nanopore N50 of 5.4 kb.

*E. muelleri* gDNA was isolated using the Nanobind Tissue (Circulomics, catalogue no. NB-900-701-01) from 177 mg of frozen tissue of clonal juvenile sponges hatched from overwintering cysts (gemmules). Gemmules were obtained from the head tank of the Kapoor Tunnel (Sooke Reservoir), part of the drinking water system of the city of Victoria, British Columbia, Canada^21^. Short DNA fragments of less than 10 kb were removed with Short Read Eliminator Kit (Circulomics, catalogue no. SS-100-101-01). gDNA was quantified with a Qubit fluorometer and sequenced on an Oxford Nanopore using a PromethION flow cell (R9.4), producing 5.31 million reads with an estimated Oxford Nanopore N50 of 18.97 kb.

To reduce the level of heterozygosity during the assembly of *M. leidyi* genome (below), an animal culture was established from a single individual through self-fertilization. High molecular weight DNA was isolated from 5–8 animals (3–5 cm) starved for 24 h before flash-freezing. Frozen tissues were powdered with mortar and pestle, dissolved in 10 ml of urea extraction buffer (50 mM Tris-HCl (pH 8.0), 7 M Urea, 312.5 mM NaCl, 20 mM EDTA (pH 8.0), 1% w/v *N*-lauroylsarcosine sodium salt) as described in ref. ^66^ and incubated for 10 min at room temperature on a rocking platform 20 rpm. gDNA was then purified twice with a phenol-chloroform-isoamyl alcohol mixture pH 7.7–8.3 (Sigma-Aldrich, catalogue no. 77617), precipitated with 0.7 volume of 100% isopropanol and subsequently washed twice with 70% ethanol. Finally, the isolated DNA was subjected to another round of purification with Nanobind Tissue kit (Circulomics, catalogue no. NB-900-701-01), followed by short-read elimination with the Short Read Eliminator Kit (Circulomics, catalogue no. SS-100-101-01). Sequencing was performed on Oxford Nanopore using PromethION flow cell (R9.4). We obtained 4.54 million reads with an estimated Oxford Nanopore N50 of 36.84 kb.

### ATAC-seq library preparation

ATAC-seq libraries from *M. leidyi* and from sorted choanocytes of *E. muelleri* were prepared using Omni-ATAC protocol as described previously^67^. Briefly, two *M. leidyi* adult specimens were dissociated using CMFSW with 0.25% α-Chymotrypsin (Sigma-Aldrich, catalogue no. C8946). To isolate nuclei, dissociated cells were transferred into cold hypotonic ATAC lysis buffer adjusted for marine animals (10 mM Tris-HCl (pH 7.5), 35 mM NaCl, 3 mM MgCl_2,_ 0.1% Tween-20, 0.01% NP-40, 0.01% digitonin, 70 µM Pitstop (Abcam, AB1206875MG)). Cell lysis was stopped after 2 min by adding marine ATAC wash buffer (10 mM Tris-HCl (pH 7.5), 35 mM NaCl, 3 mM MgCl_2_, 0.1% Tween-20, 1% BSA). Nuclei were then pelleted and resuspended in cold PBS buffer with 0.8 M Sorbitol. We used 50,000 nuclei per each tagmentation reaction.

To sort choanocytes of *E. muelleri*, 7 days posthatching sponges were fed with 0.5 µm fluorescent carboxylate-modified FluoSpheres (Invitrogen, catalogue no. F8813). After 10 min of incubation, sponges were washed twice with Strekal’s media, collected and dissociated for 15 min at 28 °C using Protease XIV (Sigma-Aldrich, catalogue no. P5147) in Strekal’s media and 1 mM CaCl_2_. Cells were pelleted at 800*g* for 5 min at room temperature and resuspended in Strekal’s media with 2 mM EDTA (pH 8.0). Further dissociation and trituration of sponge tissue continued for another 15 min at room temperature. Cells were filtered through a 40-µm cell strainer, stained with 2 µg ml^−1^ Hoechst 33342 and sorted using FACS. Sorted cells were lysed for 3 min in ATAC lysis buffer (10 mM Tris-HCl (pH 7.5), 10 mM NaCl, 3 mM MgCl_2_, 0.1% NP-40, 0.1% Tween-40, 0.01% digitonin). For each tagmentation reaction we used 100,000 nuclei. ATAC-seq libraries were prepared as described previously^67^ and sequenced on Illumina NextSeq 500 using High-Output 75 cycles.

### iChIP–seq library preparation

For *S. arctica*, *C. owczarzaki*, *S. rosetta*, *M. leidyi*, *E. muellleri*, *T. adhaerens*, *C. collaboinventa* and *N. vectensis*, double-crosslinked cells, as above, were washed with PBS, resuspended in 500 µl of cell lysis buffer (20 mM HEPES (pH 7.5), 10 mM NaCl, 0.2% IGEPAL CA-630, 5 mM EDTA, protease inhibitors cocktail) and incubated on ice for 10 min. Samples were centrifuged at 16,000*g* for 10 min at 4 °C. The resulting pellets were resuspended in bead beating buffer (20 mM HEPES (pH 7.5), 10 mM NaCl, 5 mM EDTA, protease inhibitors cocktail), and then transferred to 0.2 ml tubes containing acid-washed glass beads (Sigma-Aldrich, G8772). Cells were lysed by vortexing five times for 30 s. The supernatant was transferred to a 1.5-ml sonication tube, SDS was added to 0.6% and samples were sonicated 3–5 cycles of 30 s on, 30 s off in a Bioruptor Pico (Diagenode) to generate 200–300 bp fragments. Chromatin was diluted with 5 volumes of dilution buffer (20 mM HEPES (pH 7.5), 140 mM NaCl), centrifuged at 16,000*g* for 10 min at 4 °C and stored at −80 °C before use.

Chromatin immunoprecipitation was performed as previously described^68^ with the following modifications. Briefly, for each species 100 ng of chromatin was used for immunoprecipitation. The pool of chromatin was incubated for 14–16 h at 4 °C with 5 µl (1:50 dilution) of anti-H3K4me1 (Cell Signaling, catalogue no. 5326), 6 µl (3.4 µg) of anti-H3K4me2 (Abcam, catalogue no. ab32356), 2.5 µl (1:100 dilution) of anti-H3K4me3 (Millipore, catalogue no. 07-473), 5 µl (5 µg) of anti-SMC1 (Thermo Fisher, A300-055A) or 2 µl (2 µg) of anti-H3 (Abcam, catalogue no. ab1791) and recovered using a 1:1 mix of Protein A (Sigma-Aldrich, catalogue no. 16-661) and Protein G (Sigma-Aldrich, catalogue no. 16-662) magnetic beads. Immunoprecipitated complexes were washed, reverse crosslinked for 3 h at 68 °C, deproteinased and then purified using Ampure XP beads (Beckman Coulter, catalogue no. A63881). Final libraries were prepared using the NEBNext Ultra II DNA Library Prep Kit (New England BioLabs) according to the manufacturer’s protocol. ChIP–seq libraries were sequenced on Illumina NextSeq 500 sequencer using High-Output 75 cycles.

### MARS-seq library preparation

Single-cell libraries were prepared from freshly dissociated and sorted choanocytes of *E. muelleri* as previously described^8^. To collect cells for MARS-seq libraries, 7 days posthatching, sponges were fed with 0.5 µm fluorescent carboxylate-modified FluoSpheres (Invitrogen, catalogue no. F8813). Animal tissues were dissociated and prepared for sorting as described above for ATAC-seq. Dissociated cells were sorted through FACS into four 384-well MARS-seq plates. In total, 1,536 single-cell libraries were prepared and sequenced on an Illumina NextSeq 500 using High-Output 75 cycles.

### Chromatin proteomics

Chromatin proteomics samples were prepared as previously descibed^69^ with minor modifications. Briefly, double-crosslinked cells of *M. leidyi* (1 million per replicate) were solubilized in 1 ml of lysis buffer (4 M guanidine thiocyanate, 100 mM Tris-HCl (pH 8.0), 10 mM EDTA, 2% *N*-lauroylsarcosine sodium salt) and incubated for 10 min. Next, before adding DNA-binding beads (Invitrogen, catalogue no. 37002D), cell lysate was mixed with 1 ml of 2-propanol. The beads were separated on a magnet and the supernatant was saved as the unbound control. The beads were washed using 1 ml of wash buffer (1:1 lysis buffer to 2-propanol ratio), transferred to a 1.5-ml sonication tube and washed again with 1 ml of 80% ethanol. The chromatin was then eluted in 200 µl of 10 mM Tris-HCl (pH 8.0) containing proteinase inhibitors (Roche, catalogue no. 04693132001) and sonicated using a Bioruptor Pico at 4 °C for 3 cycles (30 s ON, 30 s OFF). To remove RNA-binding proteins, RNase A (Roche, catalogue no. 10109142001) was added to the sonicated samples, which were then incubated at 37 °C with agitation in a thermomixer. Afterwards, chromatin was re-bound to the beads by adding 250 µl of lysis buffer, vortexing and then sequentially adding 300 µl of 2-propanol. The beads were washed twice with 1 ml of 80% ethanol, and proteins were digested on the beads using trypsin (Promega, catalogue no. V5111) and LysC (NEB, catalogue no. P8109S).

### Chromatographic and mass spectrometric analysis

Samples were analysed using an Orbitrap Eclipse mass spectrometer (Thermo Fisher Scientific) coupled to an EASY-nLC 1200 (Thermo Fisher Scientific (Proxeon)). Peptides were loaded directly onto the analytical column and were separated by reversed-phase chromatography using a 50-cm column with an inner diameter of 75 μm, packed with 2-μm C18 particles (Thermo Fisher Scientific, catalogue no. ES903).

Chromatographic gradients started at 95% buffer A (0.1% formic acid in water) and 5% buffer B (0.1% formic acid in 80% acetonitrile) with a flow rate of 300 nl min^−1^ and gradually increased to 25% buffer B and 75% A in 52 min and then to 40% buffer B and 60% A in 8 min. After each analysis, the column was washed for 10 min with 100% buffer B.

The mass spectrometer was operated in positive ionization mode with nanospray voltage set at 2.4 kV and source temperature at 305 °C. The acquisition was performed in data-dependent acquisition mode and full mass spectrometry scans with one micro-scan at resolution of 120,000 were used over a mass range of *m/z* 350–1,400 with detection in the Orbitrap mass analyser. Automatic gain control was set to ‘standard’ and injection time to ‘auto’. In each cycle of data-dependent acquisition analysis, following each survey scan, the most intense ions above a threshold ion count of 10,000 were selected for fragmentation. The number of selected precursor ions for fragmentation was determined by the ‘Top Speed’ acquisition algorithm and a dynamic exclusion of 60 s. Fragment ion spectra were produced by means of high-energy collision dissociation at normalized collision energy of 28% and they were acquired in the ion trap mass analyser. Automatic gain control and injection time were set to ‘Standard’ and ‘Dynamic’, respectively, and an isolation window of 1.4 *m/z* was used. Digested bovine serum albumin (NEB, catalogue no. P8108S) was analysed between each sample to avoid sample carryover and to assure stability of the instrument, and Qcloud^70^ was used to control instrument longitudinal performance during the project.

Acquired spectra were analysed using the Proteome Discoverer software suite (v.2.5, Thermo Fisher Scientific) and the Mascot search engine (v.2.6, Matrix Science^71^). The data were searches against *M. leidyi* database and a list of common contaminants (16,042 entries)^72^ as well as all the corresponding decoy entries. For the peptide identification a precursor ion mass tolerance of 7 ppm was used for the MS1 level, trypsin was chosen as enzyme and up to three missed cleavages were allowed. The fragment ion mass tolerance was set to 0.5 Da for MS2 spectra. Oxidation of methionine and N-terminal protein acetylation were used as variable modifications whereas carbamidomethylation on cysteines was set as a fixed modification. False discovery rate in peptide identification was set to a maximum of 1%.

Peptide quantification data were retrieved from the ‘Precursor ions quantifier’ node from Proteome Discoverer (v.2.5) using 2-ppm mass tolerance for the peptide extracted ion current. The obtained values were used to calculate protein fold-changes and their corresponding *P* value and adjusted *P* values.

### DAP-seq (DNA affinity purification sequencing) library preparation

The DNA-binding domains of candidate zf-C2H2 proteins were cloned from complementary DNA (cDNA) library of *M. leidyi* into the pIX-HALO vector using NEBuilder HiFi DNA Assembly Master Mix (NEB, catalogue no. E2621). The obtained HALO-fusion constructs were translated using the TnT SP6High-Yield Wheat Germ Protein Expression System (Promega, catalogue no. L3260). Next, an adapter-ligated DNA library was prepared from native gDNA of *M. leidyi* using NEBNext Ultra II FS DNA library prep kit (NEB, catalogue no. E7805) or PCR amplified gDNA. The binding to HALO-zf-C2H2 fusion proteins and recovery of adapter-ligated gDNA libraries was performed as described in ref. ^73^. The generated DAP-seq libraries were sequenced in paired-end mode on an Illumina NextSeq 500 using High-Output 75 cycles.

### De novo genome assembly and scaffolding

We made preliminary genome assemblies of *C. owczarzaki* from Oxford Nanopore reads basecalled by Guppy v.6.0.1 using NextDenovo v.2.5.0 (ref. ^74^), Flye v.2.9.0 (ref. ^75^) and NECAT v.0.0.1 (ref. ^76^), which produced 20, 141 and 56 contigs including the mitochondrial genome, respectively. For the Flye assembly, we only used 5,000 bp or longer reads. We then integrated the three assemblies by manually comparing them to each other, with a help of reciprocal large-scale alignments generated with minimap2 (ref. ^77^). The integrated assembly was polished with the Nanopore reads using Flye ten times, and with Illumina reads^78^ using HyPo^79^ twice. A chromosome-scale duplication, which was in the end included in chromosome 15 after the 3D assembly (Extended Data Fig. 2d), was temporarily removed before annotating the genome. Finally, we manually inspected the whole assembly sequence together with the mapped Illumina data, Nanopore data and the previous Sanger sequence data^25^, and navigated them on the Integrative Genomic Viewer (IGV)^80^ to find and fix errors occurred during the consensus calling. We also manually phased chimeric haplotypes for some genes using the long reads. In total, 7,937 nucleotides were manually inserted or deleted at 430 sites and 1,193 nucleotides were substituted at 1,081 sites.

We produced two new genome assemblies for *E. muelleri* and *M. leidyi*. In both cases, we used Oxford Nanopore reads after base call correction using Guppy v.5.0.17 (using the dna_r9.4.1_450bps_sup_prom.cfg configuration the super-accurate base calling model, and a filtering reads with min_qscore=10). Then, we used two different long-read assemblers (Flye v.2.9-b1768, ref. ^75^ and Shasta v.0.8.0, ref. ^81^) and various assembly strategies (filtering by read length at 0, 10 and 50 kb), and selected the best resulting draft assemblies for each species. To that end, we evaluated the contiguity (measured using the contig N50), completeness and occurrence of uncollapsed haplotypes for each draft (Extended Data Fig. 2a,b). Contiguity was evaluated using total assembly length and contig N50. Completeness was measured with the fraction of conserved orthologues recovered by BUSCO v.5.1.2 (ref. ^82^) (using the genome mode and the metazoa_odb10) and the fraction of mappable genes from the original assemblies (mapped using Liftoff v.1.6.1, ref. ^83^). The presence of uncollapsed haplotypes was assessed with the distribution of per-base sequencing depths, calculated using the pbcstat utility in purge_dups v.1.2.5 (ref. ^84^) (for which we remapped the input reads to the assembly with minimap2 2.18-r1015 (ref. ^77^), using the -x map-ont preset for long-read mapping) (Extended Data Fig. 2e,f).

The best drafts for each species were produced using the following parameter combinations: (1) for *E. muelleri*, we used the Shasta assembler with the Nanopore configuration (--config Nanopore-Oct2021 flag), without filtering by read length (estimated sequencing depth roughly 100×) and (2) for *M. leidyi*, we used Flye with reads filtered at 50 kb (estimated sequencing depth roughly 150×), the raw Nanopore read configuration (--nano-raw flag) and an estimated total assembly size of 200 Mb.

Then, we used purge_dups to collapse putative uncollapsed haplotypes in each assembly, in the following manner: (1) we split the assembly into contigs with the split_fa utility; (2) we aligned the genome to itself with minimap2 and the -x asm5 preset; (3) we used the read alignments to the unsplit assembly (produced with minimap2 -x map-ont) to obtain the sequencing depth histogram and calculate coverage cutoffs with pbcstat and calcuts, respectively; (4) we used these cutoffs and the mapped reads to remove haplotigs and overlaps for the draft, with purge_dups proper and using two rounds of alignment chaining (-2 flag) and finally (5) we reevaluated the assembly quality using per-base sequencing depth distributions (above) and reductions in the fraction of duplicated BUSCO orthologues.

### Chromosome-level assembly

To obtain chromosome-level genome assemblies, generated Micro-C libraries were mapped to de novo draft genome assemblies (*C. owczarzaki*, *E. muelleri* and *M. leidyi*) or current genome assemblies (*T. adhaerens* ASM15027v1, ref. ^22^, *S. arctica*^24^, *S. rosetta* GCA_000188695.1, ref. ^26^, *C. collaboinventa*^85^) using Juicer v.1.6 (ref. ^86^) with an option -p assembly. Proximity ligation alignments were used by 3D de novo assembly pipeline^87^ to order and orient available contigs into chromosomes with the following parameters: *S. arctica* -r 3 --editor-repeat-coverage 10, *C. owczarzaki* -r 0 --editor-repeat-coverage 4, *S. rosetta* -r 3 --editor-repeat-coverage 2, *E. muelleri* -r 2 --editor-repeat-coverage 10, *M. leidyi* -r 2 -i 1000 --editor-repeat-coverage 2, *T. adhaerens* -r 3 --editor-repeat-coverage 2 and *C. collaboinventa* -r 3 --editor-repeat-coverage 2. The resulting assemblies were manually reviewed and corrected with Juicebox Assembly Tools^88^ (Extended Data Fig. 2c–f). Finally, chromosome-level genome assemblies were polished with Medaka (v.1.5.0) to correct possible sequence errors such as indels and mismatches, as follows: (1) first, we mapped the Nanopore reads to the chromosome-level assembly using the minimap2-based mini_align utility; (2) we then used Medaka consensus to obtain consensus sequences, specifying a batch size of 200 (--batch 200 flag) and the r941_prom_sup_g507 configuration (--model flag) and (3) we merged the consensus and variant calls for all chromosomes into a polished assembly using Medaka stitch.

### Genome annotation

To annotate the *C. owczarzaki* genome, we did not mask the repeats because the intergenic regions are very small^25^ and, thus, masking only increased annotation failure on duplicated genes. We used BRAKER2 (ref. ^89^) with OrthoDB^90^ protein sequence collections as hint data, as well as with RNA-seq data from a previous study^61^. The three preliminary annotations, evidenced by metazoan proteins, protozoan proteins and RNA-seq data, were combined with TSEBRA^91^, giving rise to 9,069 annotated transcripts. Finally, we manually searched and fixed wrong annotations by navigating the assembly on IGV^80^, comparing the combined annotation with the three preliminary annotations together with the mapped RNA-seq data. By this careful inspection, we modified or newly annotated 1,871 transcripts including alternatively spliced ones. Compared to the previously published proteome^25^ (v.2), only 4,076 out of 8,792 proteins (including alternatively spliced ones) had completely matched sequences to the those predicted in this study, allowing simple amino acid mismatches probably accounting for polymorphisms.

To annotate *M. leidyi* genome we first downloaded developmental Illumina RNA-seq samples (GSE93977), trimmed them with fastp and built a de novo Trinity assembly, which was mapped to the genome using gmap^92^. The RNA-seq was also directly mapped to genome using HISAT2 (ref. ^93^) with the –dta parameter, and genome-based transcriptomes were built for each sample using StringTie^94^. Merged mapped RNA-seq samples were then used to find high-quality intron junctions using Portcullis. The combination of Trinity, StringTie and Portcullis intron junctions were then fed to Mikado for transcript selection. The best resulting gene models based on mapping to UniProt were then used to train an Augustus model for *M. leidyi*. Augustus was used for an ab initio gene prediction, using exonic hints from Mikado, intron hints from Portcullis and coding sequence hints from a MetaEuk^95^ run with query fasta files combining proteins from *H. californensis* and UniProt. Mikado transcripts and Augustus gene models were then merged using EVidenceModeler (scores of 10 for Mikado transcripts and 2 for Augustus gene models). The resulting gene models were updated with PASA^96^ to incorporate the untranslated regions from the Mikado transcripts.

To annotate *S. arctica*, *S. rosetta*, *E. muelleri*, *T. adhaerens* and *C. collaboinventa* genome assemblies, gene models from previous assemblies were mapped onto new coordinates using Liftoff (v.1.6.1)^83^ with -overlap 1 -flank 1 options.

### Repeat annotation

Repetitive sequences and transposable elements were annotated using EDTA (v.2.1.0)^97^ with the following parameters: --sensitive 1 --anno 1 (Extended Data Fig. 2g). For *H. sapiens*, we used RepeatMasker (v.open-4-0-3) annotation of GRCh38 genome released by UCSC.

### DNA methylation calling from Nanopore long-read sequencing data

The fast5 files obtained from the PromethION were used as input for Megalodon (v.2.5), with the Remora model dna_r9.4.1_e8 sup for 5hmc_5mc modification only on CG dinucleotides. We then built bigwig files using the bedGraphToBigWig tool from UCSC. The Megalodon CG methylation calls were compared to previously published Whole-Genome Bisulfite Sequencing remapped to the new reference genomes using Bismark (SRR8346013 and SRR10356110)^21,48^. Both data sources were congruent, yet Nanopore had deeper and broader coverage, we used Megalodon methylation data for subsequent analysis.

### Micro-C data processing

Micro-C data were processed using the 4D Nucleome processing pipeline^98^. Briefly, raw reads were mapped to the reference genome using bwa mem (v.0.7.17-r1188) with the -SP5M option. The mapped reads were sorted and filtered with pairtools (v.0.3.0)^99^. Pairs that mapped within a 2-bp distance from each other were considered duplicates. We also discarded reads mapping within the distance of 200 bp, which eliminates self-ligated pairs and reads mapping to adjacent nucleosomes. Only uniquely mapping pairs and 5′ most unique alignments of multiple ligations pairs were aggregated into 200-bp bin contact matrices and multiresolution .cool or .hic files (Extended Data Fig. 1d). Contact matrices were normalized with cooler (v.0.8.11)^100^ using the iterative correction and eigenvector (ICE) balancing method^101^ for .cool files or with Juicer tools^86^ using Knight–Ruiz balancing^102^ for .hic files. All contact heatmaps were visualized with either Cooltools (v.0.5.1)^103^ or Coolbox (v.0.3.8)^104^ and genome assembly heatmaps were visualized using HiGlass^105^.

Reproducibility between replicates was estimated using the stratum-adjusted correlation coefficient (SCC) implemented in HiCRep^106^ at resolutions of 1, 2, 5, 10, 25 and 50 kb (Extended Data Fig. 1f). The SCC scores were averaged across chromosomes. Biological replicates with SCC score estimated above 0.7 at resolutions equivalent to roughly 20,000 bins per species genome (resolution of 10 kb for *S. arctica*, 1 kb for *C. owczarzaki*, 2 kb for *S. rosetta*, 10 kb for *E. muelleri*, 10 kb for *M. leidyi*, 5 kb for *H. californensis*, 5 kb for *T. adhaerens*, 5 kb for *C. collaboinventa* and 10 kb for *N. vectensis*) were pooled to obtain final chromatin interaction matrices. Technical replicates were first merged, deduplicated and only then combined into the final contact maps.

The decay of the average contact frequency over genomic distance from 1 kb to 100 Mb was calculated using Cooltools (v.0.5.1)^103^. The decay curves were calculated for each chromosome separately, and then averaged across chromosomes (Extended Data Fig. 1g).

### Compartment analysis

Compartment analysis was performed on observed-over-expected contact maps at resolutions equivalent to 5,000, 10,000, 20,000 and 50,000 bins per species genome (Extended Data Fig. 3a) using Cooltools eigs-cis^103^. We visually examined calculated eigenvectors, and, for each organism, the E1 vector corresponded to the compartmentalization pattern of contact maps. Active (A) and inactive (B) compartment types were assigned by GC content (for all species except *C. owczarzaki*) or H3K4me3 chromatin signal for *C. owczarzaki*, such that higher GC regions or positively correlated with H3K4me3 signal regions correspond to A compartment. Saddle plots were generated using the Cooltools saddle module. Specifically, the eigenvectors were sorted from lowest to highest value and combined into 40 groups according to their eigenvector value. The first (bottom 2.5% E1 values) and last (top 2.5% E1 values) groups were ignored to exclude potential outliers. The observed-over-expected value of the remaining 38 groups was averaged across all bins and chromosomes and visualized as saddle plots.

Compartment strength was calculated as the ratio of homotypic (AA + BB) over heterotypic (AB + BA) compartment contacts. We choose the top 20% of observed-over-expected values for both homotypic and heterotypic interactions. To assess the error in estimating compartment strength, we compared the compartments strength across different resolutions as well as performed visual inspection of the contact maps (Extended Data Fig. 3b). The latter showed varying degrees of accuracy in identifying compartment types between species, with the algorithm performing particularly poorly on *M. leidyi* due to the lack of well-defined chromatin compartmentalization in this species in our sample. Therefore, we assigned an extra intermediate compartment I to the intermediate eigenvalues close to zero. To that end, we modelled the genome-wide eigenvalues distribution as a Gaussian mixture with three components using the normalmixEM function from the mixtools R package (v.2.0.0) as described^107^. The B–I and I–A thresholds were defined as intersection points between components (Extended Data Fig. 3c).

To characterize the distribution of genomic features in the A, I and B compartments, we calculated cumulative H3K4me3 chromatin signals and RNA-seq expression values for each compartment region. Furthermore, we estimated the percentage of bases annotated as transposable elements or coding gene regions within these compartments. All the values are presented as −log_2_(1 − the value’s quantile). Thus, a normalized value of six means that the coverage is in the upper 1–2^−6^ quantile, that is, in the upper 1/64th of the distribution (Extended Data Fig. 3d).

### Insulation profiles and boundaries classification

To compute the insulation profiles, we first determined the optimal resolution and window sizes for a target genome. To that end, we calculated insulation scores using Cooltools insulation module^103^ at resolutions roughly equivalent to 50,000, 100,000, 200,000 and 400,000 genomic bins per species genome with a sliding window for each resolution that is ×5, ×10 and ×25 the applied resolution (Extended Data Fig. 4a). The resolution and two window sizes with maximum average insulation scores were considered optimal because they reflected the strongest partitioning of genomes into isolated domains. Insulation boundaries located within two bins of unmappable genomic region were removed.

Identified insulation boundaries were categorized into strong and weak using the peak prominence of their boundary strength distributions (Li threshold) as implemented in the Cooltools insulation score module. Strong boundaries were further annotated with overlapping genomic features that fall within one bin of the annotated feature from the insulation boundary. For example, if compartment boundaries were called at the resolution 5 kb, then the maximum distance to the closest insulation boundary is ±10 kb.

To estimate internal interactions within contact domains, rescaled pile-ups were generated using coolpup.py^108^. Contact domains were defined as valleys between two strongly insulated regions, which were not further from each other than 100 kb.

### Loop calling and annotation

Chromatin loops were identified using SIP v.1.6.1 (ref. ^109^) on KR-normalized contact matrices. In *M. leidyi*, the SIP peak caller was applied with the following parameters: -norm KR -g 3.0 -min 2.0 -max 2.0 -mat 5000 -d 10 -res 400 -sat 0.01 -t 2000 -nbZero 6 -factor 4 -fdr 0.05 -isDroso false. For *T. adhaerens*, chromatin loops were called with the following parameters: -norm KR -g 5.0 -min 4.0 -max 4.0 -mat 5000 -d 20 -res 100 -sat 0.01 -t 2000 -nbZero 6 -factor 4 -fdr 0.05 -isDroso false; for *C. collaboinventa*: -norm VC_SQRT -g 1.5 -min 3.0 -max 3.0 -mat 5000 -d 20 -res 500 -sat 0.01 -t 2000 -nbZero 6 -factor 2 -fdr 0.05 -isDroso false; for *H. californensis*: -norm KR -g 2.5 -min 3.0 -max 3.0 -mat 5000 -d 10 -res 1000 -sat 0.01 -t 2000 -nbZero 6 -factor 4 -fdr 0.05 -isDroso false. Identified loops were then filtered based on APSscore, removing high-intensity signals outside the normal distribution of APSscore values. This threshold ensured accurate removal of false positive regions that corresponded to structural genomic rearrangements, such as inversions or assembly artefacts. For *H. californensis*, we kept only annotated loops with values greater than ten. Chromatin loops in *N. vectensis* and focal chromatin contacts in *E. muelleri* were annotated manually.

Each loop anchor was assigned a promoter or enhancer identity based on their epigenetic signature. We calculated quantile normalized counts per million (CPM) coverage of H3K4me3, H3K4me2 and H3K4me1 ChIP signals in 1-kb (*T. adhaerens*, *C. collaboinventa*, *N. vectensis*), 2-kb (*M. leidyi*, *E. muelleri*, *D. melanogaster*) or 10-kb (*H. sapiens*) windows from a centre of a loop anchor.

### METALoci autocorrelation analysis

METALoci^110^ (v.0.3.0) analysis was applied to explore the spatial distribution and autocorrelation of epigenetic signal in *T. adhaerens* contact maps. For each region of interest at 800 bp resolution, the top 20% pairs of contacts were used to create a two-dimensional graph layout by means of the Kamada–Kawai algorithm^111^ (Fig. 3b, top left panel) using the ‘metaloci layout’ with default parameters. Next, the signal of interest (H3K4me3 ChIP, ATAC, genic exon annotation) measured in the 800-bp genomic bin was mapped onto the built graph layout using ‘metaloci lm’ with default parameters. Spatial and epigenetic signals were embedded into Voronoi diagrams for enhanced visualization as a Gaudí plot (Extended Data Fig. 8c), and the local Moran’s index (LMI) analysis^112,113^ was applied for each bin of the Gaudí plot.

According to LMI analysis, each bin is assigned to one of the four distinct groups, called LMI quadrants, based on the signal value in a bin and average signal value in its neighbourhood. If a bin and its neighbourhood have similar amounts of signal (low or high), then this bin is assigned to a low–low (blue) or high–high (red) quadrant. If a bin and its neighbourhood have different amounts of signal, then the bin is assigned to a low–high (cyan) or high–low (orange) quadrants, respectively. Significantly colocalized bins according to LMI, in which a *P* value is obtained using a permutation test, are highlighted by colour in the LMI scatterplots (Extended Data Fig. 8c,j, left panels). An analogous colouring scheme is applied to the Voronoi diagrams of the Gaudí plots. Hence, the highlighted blue and red bins on a Gaudí plot represent bins in which the signal is significantly colocalized in the space. Thus, ATAC-seq, H3K4me3 and motif score signals are significantly enriched inside the nested focal contacts (Fig. 3b and Extended Data Fig. 8c), whereas exons are significantly enriched outside loop contacts (Fig. 3b). METALoci code is available at the GitHub repository (https://github.com/3DGenomes/METALoci).

### Motif analysis

Loop anchor regions of *M. leidyi* (*n* = 8,523) and *H. californensis* (*n* = 478) were scanned for enriched motifs with HOMER^114^ in de novo motif discovery mode. As background sequences, we used random genomic regions of equivalent size and GC content (*n* = 38,810 in *M. leidyi* and *n* = 49,097 in *H. californensis*). For motif enrichment analysis in *T. adhaerens* (*n* = 3,557) and *C. collaboinventa* (*n* = 4,037), we scanned loop anchor regions using random genomic sequences of equivalent size (*n* = 32,004 in *T. adhaerens* and *n* = 36,178 in *C. collaboinventa*) as background. Loop anchor regions of *N. vectensis* (*n* = 327) were scanned for enriched motif using random genomic sequences (*n* = 45,268) of equivalent size and GC content as background. In addition, we used ATAC-seq accessible neuronal promoter regions (*n* = 22,961) as background to scan for enriched motifs in genomic regions that overlap ATAC-seq peaks located at the non-loop insulation boundaries (*n* = 9,016). To annotate genomic regions with identified motifs, we used the monaLisa package^115^, selecting percentile threshold of motif scores by comparing the motif score distributions in the target regions with genome-wide motif score distributions (Extended Data Figs. 7d,f, 8i and 9f).

### Whole-genome alignment and sequence conservation analyses

We evaluated the degree of sequence conservation of the *M. leidyi* genome by comparing it to other ctenophores (*B. microptera*, *P. bachei* and *Hormiphora californiensis*). To that end, we first aligned all genomes to each other using Cactus v.2.6.4 (ref. ^116^), following a progressive approach guided by the species trees of ctenophores, namely: ((*M. leidyi*, *B. microptera*), (*H. californiensis*, *P. bachei*)). Second, we used the hal2maf utility from the HAL toolkit v.2.2 (ref. ^117^) to create MAF (multiple alignment format) alignments of each chromosome, using *M. leidyi* as reference. To identify conserved regions in these genomes, we used the rphast v.1.6.1 implementation of the Phast toolkit^118^, as follows: (1) we used phyloFit^119^ to create an initial null model of neutral change based on the fourfold degenerate codon positions of each genome’s coding regions, using a general reversible nucleotide transition matrix and the predefined species tree; (2) we used phastCons to optimize this model using the expectation–maximization procedure, re-estimating transition probabilities and tree parameters at each step (the optimization step was performed using only the longest chromosome in each genome).

### Loop synteny analysis in *M. leidyi*

We evaluated the degree of syntenic conservation of the loop regions in *M. leidyi* compared to the other ctenophore genomes, and compared it to that of length-matched regions not involved in loops. To that end, we first identified orthologous genes across the four ctenophore species (*M. leidyi*, *B. microptera*, *P. bachei* and *H. californensis*) using Broccoli v.1.2 (ref. ^120^) to obtain orthologous gene pairs (step 4), using predicted peptide sequences as input (longest isoform per gene only). Within Broccoli, we used the maximum-likelihood gene tree inference algorithm (based on IQ-TREE^121^) and set a *k*-mer length of 10,000 to avoid the removal of paralogous sequences from the analysis. Second, we mapped pairs of loop anchor regions from *M. leidyi* (2,353 pairs of promoter–enhancer and 99 promoter–promoter loops, *n* = 2,452 in total) to their closest overlapping genes, and used these genes and their orthologs as anchors to map these regions to the other ctenophore genomes. In parallel, we randomly selected length-matched, non-loop overlapping regions from the *M. leidyi* genome to compare their synteny conservation with that of loop regions (using the randomizeRegions function in the regioneR R package^122^ to select 3× background regions, *n* = 7,356). Then, for each pair of species, we evaluated the synteny conservation of the foreground (loop) and background regions (random non-loops) from the point of view of the flanking synteny-anchoring genes, using two different metrics: (1) the fraction of shared orthologous genes between the flanking genes across all regions in the foreground and background sets (testing the significance of the difference using a *χ*^2^ test for given probabilities) and (2) the distributions of per-region shared orthologs (tested using the one-sided Wilcoxon rank sum test with continuity correction).

### ATAC-seq analysis

We used previously published datasets of ATAC-seq for *C. owczarzaki*^17^, *S. rosetta*^39^, *T. adhaerens*^123^, *C. collaboinventa*^123^, *D. melanogaster*^124^ and *H. sapiens*^125^ as well as newly generated datasets for *N. vectensis*, *M. leidyi* and *E. muelleri*. Sequenced reads were demultiplexed and converted to fastq files using bcl2fastq v.2.20 Illumina. Raw reads were filtered and trimmed with Trimmomatic (v.0.39)^126^ before mapping to the reference genome with bwa mem (v.0.7.17-r1188) and duplicates were marked with bamsormadup from biobambam2 (https://github.com/gt1/biobambam2). Using deeptools alignmentSieve aligned reads were filtered and shifted with -ATACshift, which corresponds to mate reads being shifted +4 and −5 bp for positive and negative strands, respectively. To generate nucleosome-position data tracks, nucleosome-free and nucleosome-bound regions were defined using the following length thresholds 0–120 and 150–240 bp, respectively. ATAC peaks were called with MACS2 (ref. ^127^) on shifted nucleosome-free regions. Footrpint ATAC score was calculated using TOBIAS v.0.13.3 (ref. ^128^).

### ChIP–seq analysis

We analysed publicly available dataset for *D. melanogaster*^129^ and *H. sapiens*^130^ and 34 newly generated ChIP–seq datasets as described below. Raw reads after removal of 3′-adapters and quality filtering with Trimmomatic (v.0.39)^126^ were aligned to the reference genome with bwa mem (v.0.7.17-r1188). Duplicated reads were marked with bamsormadup (https://github.com/gt1/biobambam2), and peaks were called using MACS2 (v.2.2.6)^127^. Aggregated density plots were visualized with deeptools (v.3.1.3)^131^.

### DAP-seq analysis

Raw reads from amplified and native gDNA fragments bound by HALO-zf-C2H2 protein fusions were analysed as described for ChIP–seq. Motif enrichment analysis was performed using HOMER^114^ in de novo motif discovery mode for MACS2 identified narrow peaks resized to 300 bp (for CTEP1 *n* = 14,638; for CTEP2 *n* = 10,615). GC- and size-normalized random genomic regions were used as background (for CTEP1 *n* = 25,964; for CTEP2 *n* = 30,744).

### RNA-seq analysis

We used previously published datasets of bulk poly-A enriched RNA-seq for *S. arctica*^132^, *C. owczarzaki*^61^, *S. rosetta*^39^, *D. melanogaster*^124^ and *H. sapiens*^133^ (Supplementary Table 2). To process data, raw reads were aligned to the reference genome using STAR (v.020201)^134^ in --quantMode to estimate the number of read counts per gene. In downstream analysis, gene counts were reported as −log_2_(1 − gene counts quantile).

### MARS-seq analysis and single-cell expression atlases

Single-cell MARS-seq libraries generated previously^8,135^ were aligned to new reference genomes of *E. muelleri* (GCA_049114765.1)*, M. leidyi* (GCA_048537945.1) and *N. vectensis* (GCA_932526225.1) using Liftoff or de novo annotated gene models. To improve single-cell RNA-seq quantification, gene annotations for *E. muelleri* and *M. leidyi* have been extended using GeneExt^136^. Briefly, MARS-seq alignment files have been subsampled to 100 M reads and MACS2 (ref. ^127^) was used to call peaks using default parameters. Intergenic peaks were filtered based on the 20th percentile of the genic peak coverage and each gene was extended to the most distant peak within 5,000 nucleotides. Metacell and clustering analyses were performed as previously described^8^. The single-cell expression atlas for *T. adhaerens* was obtained from a previously published dataset^123^.

### Public datasets used in this study

All public datasets used in this study are listed in Supplementary Table 2. ATAC-seq, ChIP–seq and RNA-seq datasets were analysed as described above.

### Reporting summary

Further information on research design is available in the Nature Portfolio Reporting Summary linked to this article.

## Online content

Any methods, additional references, Nature Portfolio reporting summaries, source data, extended data, supplementary information, acknowledgements, peer review information; details of author contributions and competing interests; and statements of data and code availability are available at 10.1038/s41586-025-08960-w.

## Supplementary information

Supplementary Text 1 Detailed Micro-C protocol used in this study.

Reporting Summary

Supplementary Table 1 Quality control metrics for each Micro-C replicate.

Supplementary Table 2 List of publicly available datasets and their corresponding sequence read archive accession numbers used in this study.

Supplementary Table 3 This file contains a list of orthologous genes for CTEP1 and CTEP2 architectural proteins in other ctenophore species, as well as a list of 358 publicly available metazoan genome datasets used for multiple sequence alignments of CTEP1 and CTEP2.

Supplementary Data 1 Phylogenetic tree of TIR sequences of placozoan Mutator DNA transposable element.

## Data Availability

Raw and processed high-throughput sequencing data are available in a Genome Expression Omnibus (GEO) repository under accession number GEO GSE260572. Raw proteomics data have been deposited to the PRIDE^101^ repository with the dataset identifier PXD056500. The de novo sequenced genome of *C. owczarzaki* is deposited under BioProject PRJDB19057; for *M. leidyi* genome, BioProject PRJNA1174117 (genome accession number GCA_048537945.1) and for the *E. muelleri* genome, BioProject PRJNA1175447 (genome accession number GCA_049114765.1). Furthermore, sequenced and assembled genome sequences, genome annotations and genomic intervals used in this study, such as chromatin loop anchors, insulation boundaries and compartmentalization domains are also available on GitHub (https://github.com/sebepedroslab/early-metazoa-3D-chromatin). In addition, datasets can be explored in interactive genome browsers^137^ for each species at A.S.-P.’s laboratory site (https://sebelab.crg.eu/3d-genomes-arc-jb2).

## Code availability

Scripts to reproduce the data processing and downstream analysis are available at GitHub (https://github.com/sebepedroslab/early-metazoa-3D-chromatin). Unless otherwise specified, scripts are based on R v.4.3.2 and Python v.3.7.7, and the language-specific libraries specified in the Methods section.

## References

[1] Lieberman-Aiden, E. et al. Comprehensive mapping of long range interactions reveals folding principles of the human genome. *Science* **326**, 289–293 (2009). 19815776 10.1126/science.1181369 PMC2858594

[2] Sexton, T. et al. Three-dimensional folding and functional organization principles of the *Drosophila* genome. *Cell* **148**, 458–472 (2012). 22265598 10.1016/j.cell.2012.01.010

[3] Beagan, J. A. & Phillips-Cremins, J. E. On the existence and functionality of topologically associating domains. *Nat. Genet.* **52**, 8–16 (2020). 31925403 10.1038/s41588-019-0561-1 PMC7567612

[4] Szabo, Q., Bantignies, F. & Cavalli, G. Principles of genome folding into topologically associating domains. *Sci. Adv.* **5**, eaaw1668 (2019). 30989119 10.1126/sciadv.aaw1668 PMC6457944

[5] Rao, S. S. P. et al. A 3D map of the human genome at kilobase resolution reveals principles of chromatin looping. *Cell* **159**, 1665–1680 (2014). 25497547 10.1016/j.cell.2014.11.021 PMC5635824

[6] Tanay, A. & Cavalli, G. Chromosomal domains: epigenetic contexts and functional implications of genomic compartmentalization. *Curr. Opin. Genet. Dev.* **23**, 197–203 (2013). 23414654 10.1016/j.gde.2012.12.009

[7] Acemel, R. D. & Lupiáñez, D. G. Evolution of 3D chromatin organization at different scales. *Curr. Opin. Genet. Dev.* **78**, 102019 (2023). 36603519 10.1016/j.gde.2022.102019

[8] Sebé-Pedrós, A. et al. Early metazoan cell type diversity and the evolution of multicellular gene regulation. *Nat. Ecol. Evol.* **2**, 1176–1188 (2018). 29942020 10.1038/s41559-018-0575-6 PMC6040636

[9] Schultz, D. T. et al. Ancient gene linkages support ctenophores as sister to other animals. *Nature* **618**, 110–117 (2023). 37198475 10.1038/s41586-023-05936-6 PMC10232365

[10] Sebé-Pedrós, A., Degnan, B. M. & Ruiz-Trillo, I. The origin of Metazoa: a unicellular perspective. *Nat. Rev. Genet.* **18**, 498–512 (2017). 28479598 10.1038/nrg.2017.21

[11] Hsieh, T.-H. S. et al. Resolving the 3D landscape of transcription-linked mammalian chromatin folding. *Mol. Cell* **78**, 539–553 (2020). 32213323 10.1016/j.molcel.2020.03.002 PMC7703524

[12] Krietenstein, N. et al. Ultrastructural details of mammalian chromosome architecture. *Mol. Cell* **78**, 554–565 (2020). 32213324 10.1016/j.molcel.2020.03.003 PMC7222625

[13] Brunet, T. & King, N. The origin of animal multicellularity and cell differentiation. *Dev. Cell* **43**, 124–140 (2017). 29065305 10.1016/j.devcel.2017.09.016 PMC6089241

[14] Winick-Ng, W. et al. Cell-type specialization is encoded by specific chromatin topologies. *Nature* **599**, 684–691 (2021). 34789882 10.1038/s41586-021-04081-2 PMC8612935

[15] Gaiti, F. et al. Landscape of histone modifications in a sponge reveals the origin of animal cis-regulatory complexity. *eLife* **6**, e22194 (2017). 28395144 10.7554/eLife.22194 PMC5429095

[16] Schwaiger, M. et al. Evolutionary conservation of the eumetazoan gene regulatory landscape. *Genome Res.* **24**, 639–650 (2014). 24642862 10.1101/gr.162529.113 PMC3975063

[17] Sebé-Pedrós, A. et al. The dynamic regulatory genome of capsaspora and the origin of animal multicellularity. *Cell* **165**, 1224–1237 (2016). 27114036 10.1016/j.cell.2016.03.034 PMC4877666

[18] Simion, P. et al. A large and consistent phylogenomic dataset supports sponges as the sister group to all other animals. *Curr. Biol.* **27**, 958–967 (2017). 28318975 10.1016/j.cub.2017.02.031

[19] Ryan, J. F. et al. The genome of the ctenophore *Mnemiopsis leidyi* and its implications for cell type evolution. *Science* **342**, 1242592 (2013). 24337300 10.1126/science.1242592 PMC3920664

[20] Moroz, L. L. et al. The ctenophore genome and the evolutionary origins of neural systems. *Nature* **510**, 109–114 (2014). 24847885 10.1038/nature13400 PMC4337882

[21] Kenny, N. J. et al. Tracing animal genomic evolution with the chromosomal-level assembly of the freshwater sponge *Ephydatia muelleri*. *Nat. Commun.* **11**, 3676 (2020). 32719321 10.1038/s41467-020-17397-w PMC7385117

[22] Srivastava, M. et al. The Trichoplax genome and the nature of placozoans. *Nature* **454**, 955–960 (2008). 18719581 10.1038/nature07191

[23] Putnam, N. H. et al. Sea anemone genome reveals ancestral eumetazoan gene repertoire and genomic organization. *Science* **317**, 86–94 (2007). 17615350 10.1126/science.1139158

[24] Dudin, O. et al. A unicellular relative of animals generates a layer of polarized cells by actomyosin-dependent cellularization. *eLife* **8**, e49801 (2019). 31647412 10.7554/eLife.49801 PMC6855841

[25] Suga, H. et al. The Capsaspora genome reveals a complex unicellular prehistory of animals. *Nat. Commun.* **4**, 2325 (2013). 23942320 10.1038/ncomms3325 PMC3753549

[26] Fairclough, S. R. et al. Premetazoan genome evolution and the regulation of cell differentiation in the choanoflagellate *Salpingoeca rosetta*. *Genome Biol.* **14**, R15 (2013). 23419129 10.1186/gb-2013-14-2-r15 PMC4054682

[27] Batut, P. J. et al. Genome organization controls transcriptional dynamics during development. *Science* **375**, 566–570 (2022). 35113722 10.1126/science.abi7178 PMC10368186

[28] Nichols, M. H. & Corces, V. G. Principles of 3D compartmentalization of the human genome. *Cell Rep.* **35**, 109330 (2021). 34192544 10.1016/j.celrep.2021.109330 PMC8265014

[29] Spracklin, G. et al. Diverse silent chromatin states modulate genome compartmentalization and loop extrusion barriers. *Nat. Struct. Mol. Biol.* **30**, 38–51 (2023). 36550219 10.1038/s41594-022-00892-7 PMC9851908

[30] Schwarzer, W. et al. Two independent modes of chromatin organization revealed by cohesin removal. *Nature* **551**, 51–56 (2017). 29094699 10.1038/nature24281 PMC5687303

[31] Strom, A. R. et al. Phase separation drives heterochromatin domain formation. *Nature* **547**, 241–245 (2017). 28636597 10.1038/nature22989 PMC6022742

[32] Duan, Z. et al. A three-dimensional model of the yeast genome. *Nature* **465**, 363–367 (2010). 20436457 10.1038/nature08973 PMC2874121

[33] Nand, A. et al. Genetic and spatial organization of the unusual chromosomes of the dinoflagellate *Symbiodinium microadriaticum*. *Nat. Genet.* **53**, 618–629 (2021). 33927399 10.1038/s41588-021-00841-y PMC8110479

[34] Harris, H. L. et al. Chromatin alternates between A and B compartments at kilobase scale for subgenic organization. *Nat. Commun.* **14**, 3303 (2023). 37280210 10.1038/s41467-023-38429-1 PMC10244318

[35] Weintraub, A. S. et al. YY1 is a structural regulator of enhancer-promoter loops. *Cell* **171**, 1573–1588 (2017). 29224777 10.1016/j.cell.2017.11.008 PMC5785279

[36] Heger, P., Marin, B., Bartkuhn, M., Schierenberg, E. & Wiehe, T. The chromatin insulator CTCF and the emergence of metazoan diversity. *Proc. Natl Acad. Sci. USA* **109**, 17507–17512 (2012). 23045651 10.1073/pnas.1111941109 PMC3491479

[37] Rowley, M. J. et al. Evolutionarily conserved principles predict 3D chromatin organization. *Mol. Cell* **67**, 837–852 (2017). 28826674 10.1016/j.molcel.2017.07.022 PMC5591081

[38] Levo, M. et al. Transcriptional coupling of distant regulatory genes in living embryos. *Nature* **605**, 754–760 (2022). 35508662 10.1038/s41586-022-04680-7 PMC9886134

[39] Gahan, J. M. et al. Chromatin profiling identifies putative dual roles for H3K27me3 in regulating transposons and cell type-specific genes in choanoflagellates. Preprint at *bioRxiv*10.1101/2024.05.28.596151 (2024). 10.1038/s41467-025-64570-0 PMC12572184 41162361

[40] Galitsyna, A. et al. Extrusion fountains are hallmarks of chromosome organization emerging upon zygotic genome activation. Preprint at *bioRxiv*10.1101/2023.07.15.549120 (2023). 10.1038/s41467-026-69105-9 PMC13018191 41690937

[41] Hansen, K. L. et al. Synergy between *cis*-regulatory elements can render cohesin dispensable for distal enhancer function. Preprint at *bioRxiv*10.1101/2024.10.04.615095 (2024). 10.1126/science.adt4221 PMC13446962 41308125

[42] Cazet, J. F. et al. A chromosome-scale epigenetic map of the *Hydra* genome reveals conserved regulators of cell state. *Genome Res.* **33**, 283–298 (2023). 36639202 10.1101/gr.277040.122 PMC10069465

[43] Vian, L. et al. The energetics and physiological impact of cohesin extrusion. *Cell* **173**, 1165–1178 (2018). 29706548 10.1016/j.cell.2018.03.072 PMC6065110

[44] Lam, E. Y. N., Beraldi, D., Tannahill, D. & Balasubramanian, S. G-quadruplex structures are stable and detectable in human genomic DNA. *Nat. Commun.* **4**, 1796 (2013). 23653208 10.1038/ncomms2792 PMC3736099

[45] Li, L. et al. YY1 interacts with guanine quadruplexes to regulate DNA looping and gene expression. *Nat. Chem. Biol.* **17**, 161–168 (2021). 33199912 10.1038/s41589-020-00695-1 PMC7854983

[46] Dejosez, M. et al. Regulatory architecture of housekeeping genes is driven by promoter assemblies. *Cell Rep.* **42**, 112505 (2023). 37182209 10.1016/j.celrep.2023.112505 PMC10329844

[47] Goel, V. Y., Huseyin, M. K. & Hansen, A. S. Region Capture Micro-C reveals coalescence of enhancers and promoters into nested microcompartments. *Nat. Genet.* **55**, 1048–1056 (2023). 37157000 10.1038/s41588-023-01391-1 PMC10424778

[48] de Mendoza, A. et al. Convergent evolution of a vertebrate-like methylome in a marine sponge. *Nat. Ecol. Evol.* **3**, 1464–1473 (2019). 31558833 10.1038/s41559-019-0983-2 PMC6783312

[49] Yin, Y. et al. Impact of cytosine methylation on DNA binding specificities of human transcription factors. *Science* **356**, eaaj2239 (2017). 28473536 10.1126/science.aaj2239 PMC8009048

[50] Zolotarev, N. et al. Architectural proteins Pita, Zw5, and ZIPIC contain homodimerization domain and support specific long-range interactions in *Drosophila*. *Nucleic Acids Res.* **44**, 7228–7241 (2016). 27137890 10.1093/nar/gkw371 PMC5009728

[51] Schultz, D. T. et al. A chromosome-scale genome assembly and karyotype of the ctenophore *Hormiphora californensis*. *G3* **11**, jkab302 (2021). 34545398 10.1093/g3journal/jkab302 PMC8527503

[52] Hsieh, T.-H. S., Fudenberg, G., Goloborodko, A. & Rando, O. J. Micro-C XL: assaying chromosome conformation from the nucleosome to the entire genome. *Nat. Methods* **13**, 1009–1011 (2016). 27723753 10.1038/nmeth.4025

[53] Sun, L. et al. Mapping nucleosome-resolution chromatin organization and enhancer-promoter loops in plants using Micro-C-XL. *Nat. Commun.* **15**, 35 (2024). 38167349 10.1038/s41467-023-44347-z PMC10762229

[54] Solovei, I. & Mirny, L. Spandrels of the cell nucleus. *Curr. Opin. Cell Biol.* **90**, 102421 (2024). 39180905 10.1016/j.ceb.2024.102421

[55] Olivares-Chauvet, P. et al. Capturing pairwise and multi-way chromosomal conformations using chromosomal walks. *Nature* **540**, 296–300 (2016). 27919068 10.1038/nature20158

[56] Schmidt, D. et al. Waves of retrotransposon expansion remodel genome organization and CTCF binding in multiple mammalian lineages. *Cell* **148**, 335–348 (2012). 22244452 10.1016/j.cell.2011.11.058 PMC3368268

[57] Martín-Durán, J. M. et al. Conservative route to genome compaction in a miniature annelid. *Nat. Ecol. Evol.* **5**, 231–242 (2021). 33199869 10.1038/s41559-020-01327-6 PMC7854359

[58] Schmidbaur, H. et al. Emergence of novel cephalopod gene regulation and expression through large-scale genome reorganization. *Nat. Commun.* **13**, 2172 (2022). 35449136 10.1038/s41467-022-29694-7 PMC9023564

[59] Özdemir, I. & Gambetta, M. C. The role of insulation in patterning gene expression. *Genes* **10**, 767 (2019). 31569427 10.3390/genes10100767 PMC6827083

[60] Harmston, N. et al. Topologically associating domains are ancient features that coincide with Metazoan clusters of extreme noncoding conservation. *Nat. Commun.* **8**, 441 (2017). 28874668 10.1038/s41467-017-00524-5 PMC5585340

[61] Sebé-Pedrós, A. et al. Regulated aggregative multicellularity in a close unicellular relative of metazoa. *eLife* **2**, e01287 (2013). 24368732 10.7554/eLife.01287 PMC3870316

[62] Lafontaine, D. L., Yang, L., Dekker, J. & Gibcus, J. H. Hi-C 3.0: improved protocol for genome-wide chromosome conformation capture. *Curr. Protoc.* **1**, e198 (2021). 34286910 10.1002/cpz1.198 PMC8362010

[63] Curtis, A. S. G. & Vyver, G. The control of cell adhesion in a morphogenetic system. *Development* **26**, 295–312 (1971). 5157350

[64] Alié, A. et al. The ancestral gene repertoire of animal stem cells. *Proc. Natl Acad. Sci. USA* **112**, 7093–7100 (2015). 10.1073/pnas.1514789112 PMC4697369 26644562

[65] Nakanishi, N., Renfer, E., Technau, U. & Rentzsch, F. Nervous systems of the sea anemone *Nematostella vectensis* are generated by ectoderm and endoderm and shaped by distinct mechanisms. *Development* **139**, 347–357 (2012). 22159579 10.1242/dev.071902

[66] Zimmermann, B. et al. Topological structures and syntenic conservation in sea anemone genomes. *Nat. Commun.* **14**, 8270 (2023). 38092765 10.1038/s41467-023-44080-7 PMC10719294

[67] Corces, M. R. et al. An improved ATAC-seq protocol reduces background and enables interrogation of frozen tissues. *Nat. Methods* **14**, 959–962 (2017). 28846090 10.1038/nmeth.4396 PMC5623106

[68] Lara-Astiaso, D. et al. Immunogenetics. Chromatin state dynamics during blood formation. *Science* **345**, 943–949 (2014). 25103404 10.1126/science.1256271 PMC4412442

[69] Rafiee, M.-R. et al. Chromatin-contact atlas reveals disorder-mediated protein interactions and moonlighting chromatin-associated RBPs. *Nucleic Acids Res.* **49**, 13092–13107 (2021). 34871434 10.1093/nar/gkab1180 PMC8682780

[70] Chiva, C. et al. QCloud: a cloud-based quality control system for mass spectrometry-based proteomics laboratories. *PLoS ONE* **13**, e0189209 (2018). 29324744 10.1371/journal.pone.0189209 PMC5764250

[71] Perkins, D. N., Pappin, D. J. C., Creasy, D. M. & Cottrell, J. S. Probability-based protein identification by searching sequence databases using mass spectrometry data. *Electrophoresis* **20**, 3551–3567 (1999). 10612281 10.1002/(SICI)1522-2683(19991201)20:18<3551::AID-ELPS3551>3.0.CO;2-2

[72] Beer, L. A., Liu, P., Ky, B., Barnhart, K. T. & Speicher, D. W. in *Serum/Plasma Proteomics: Methods and Protocols* (eds Greening, D. W. & Simpson, R. J.) 339–352 (Springer, 2017).

[73] Bartlett, A. et al. Mapping genome-wide transcription-factor binding sites using DAP-seq. *Nat. Protoc.* **12**, 1659–1672 (2017). 28726847 10.1038/nprot.2017.055 PMC5576341

[74] Hu, J. et al. NextDenovo: an efficient error correction and accurate assembly tool for noisy long reads. *Genome Biol.* **25**, 107 (2024). 10.1186/s13059-024-03252-4 PMC11046930 38671502

[75] Kolmogorov, M., Yuan, J., Lin, Y. & Pevzner, P. A. Assembly of long, error-prone reads using repeat graphs. *Nat. Biotechnol.* **37**, 540–546 (2019). 30936562 10.1038/s41587-019-0072-8

[76] Chen, Y. et al. Efficient assembly of nanopore reads via highly accurate and intact error correction. *Nat. Commun.* **12**, 60 (2021). 33397900 10.1038/s41467-020-20236-7 PMC7782737

[77] Li, H. Minimap2: pairwise alignment for nucleotide sequences. *Bioinformatics* **34**, 3094–3100 (2018). 29750242 10.1093/bioinformatics/bty191 PMC6137996

[78] Denbo, S. et al. Revision of the *Capsaspora* genome using read mating information adjusts the view on Premetazoan genome. *Dev. Growth Differ.* **61**, 34–42 (2019). 30585312 10.1111/dgd.12587

[79] Kundu, R., Casey, J. & Sung, W.-K. HyPo: Super fast & accurate polisher for long read genome assemblies. Preprint at *bioRxiv*10.1101/2019.12.19.882506 (2019).

[80] Robinson, J. T. et al. Integrative genomics viewer. *Nat. Biotechnol.* **29**, 24–26 (2011). 21221095 10.1038/nbt.1754 PMC3346182

[81] Shafin, K. et al. Nanopore sequencing and the Shasta toolkit enable efficient de novo assembly of eleven human genomes. *Nat. Biotechnol.* **38**, 1044–1053 (2020). 32686750 10.1038/s41587-020-0503-6 PMC7483855

[82] Manni, M., Berkeley, M. R., Seppey, M., Simão, F. A. & Zdobnov, E. M. BUSCO update: novel and streamlined workflows along with broader and deeper phylogenetic coverage for scoring of eukaryotic, prokaryotic, and viral genomes. *Mol. Biol. Evol.* **38**, 4647–4654 (2021). 34320186 10.1093/molbev/msab199 PMC8476166

[83] Shumate, A. & Salzberg, S. L. Liftoff: accurate mapping of gene annotations. *Bioinformatics* **37**, 1639–1643 (2021). 33320174 10.1093/bioinformatics/btaa1016 PMC8289374

[84] Guan, D. et al. Identifying and removing haplotypic duplication in primary genome assemblies. *Bioinformatics* **36**, 2896–2898 (2020). 31971576 10.1093/bioinformatics/btaa025 PMC7203741

[85] Tessler, M. et al. Phylogenomics and the first higher taxonomy of Placozoa, an ancient and enigmatic animal phylum. *Front. Ecol. Evol.* **10**, 1016357 (2022).

[86] Durand, N. C. et al. Juicer provides a one-click system for analyzing loop-resolution Hi-C experiments. *Cell Syst.* **3**, 95–98 (2016). 27467249 10.1016/j.cels.2016.07.002 PMC5846465

[87] Dudchenko, O. et al. De novo assembly of the *Aedes aegypti* genome using Hi-C yields chromosome-length scaffolds. *Science* **356**, 92–95 (2017). 28336562 10.1126/science.aal3327 PMC5635820

[88] Durand, N. C. et al. Juicebox provides a visualization system for Hi-C contact maps with unlimited zoom. *Cell Syst.* **3**, 99–101 (2016). 27467250 10.1016/j.cels.2015.07.012 PMC5596920

[89] Brůna, T., Hoff, K. J., Lomsadze, A., Stanke, M. & Borodovsky, M. BRAKER2: automatic eukaryotic genome annotation with GeneMark-EP+ and AUGUSTUS supported by a protein database. *NAR Genom. Bioinform.* **3**, lqaa108 (2021). 33575650 10.1093/nargab/lqaa108 PMC7787252

[90] Kuznetsov, D. et al. OrthoDB v11: annotation of orthologs in the widest sampling of organismal diversity. *Nucleic Acids Res.* **51**, 445–451 (2023). 10.1093/nar/gkac998 PMC9825584 36350662

[91] Gabriel, L., Hoff, K. J., Brůna, T., Borodovsky, M. & Stanke, M. TSEBRA: transcript selector for BRAKER. *BMC Bioinform.* **22**, 566 (2021). 10.1186/s12859-021-04482-0 PMC8620231 34823473

[92] Wu, T. D. & Watanabe, C. K. GMAP: a genomic mapping and alignment program for mRNA and EST sequences. *Bioinformatics* **21**, 1859–1875 (2005). 15728110 10.1093/bioinformatics/bti310

[93] Kim, D., Paggi, J. M., Park, C., Bennett, C. & Salzberg, S. L. Graph-based genome alignment and genotyping with HISAT2 and HISAT-genotype. *Nat. Biotechnol.* **37**, 907–915 (2019). 31375807 10.1038/s41587-019-0201-4 PMC7605509

[94] Shumate, A., Wong, B., Pertea, G. & Pertea, M. Improved transcriptome assembly using a hybrid of long and short reads with StringTie. *PLoS Comput. Biol.* **18**, e1009730 (2022). 35648784 10.1371/journal.pcbi.1009730 PMC9191730

[95] Levy Karin, E., Mirdita, M. & Söding, J. MetaEuk—sensitive, high-throughput gene discovery, and annotation for large-scale eukaryotic metagenomics. *Microbiome* **8**, 48 (2020). 32245390 10.1186/s40168-020-00808-x PMC7126354

[96] Haas, B. J. et al. Improving the *Arabidopsis* genome annotation using maximal transcript alignment assemblies. *Nucleic Acids Res.* **31**, 5654–5666 (2003). 14500829 10.1093/nar/gkg770 PMC206470

[97] Ou, S. et al. Benchmarking transposable element annotation methods for creation of a streamlined, comprehensive pipeline. *Genome Biol.* **20**, 275 (2019). 31843001 10.1186/s13059-019-1905-y PMC6913007

[98] Reiff, S. B. et al. The 4D Nucleome Data Portal as a resource for searching and visualizing curated nucleomics data. *Nat. Commun.* **13**, 2365 (2022). 35501320 10.1038/s41467-022-29697-4 PMC9061818

[99] Open2C et al. Pairtools: from sequencing data to chromosome contacts. *PLOS Comput. Biol.* **20**, e1012164 (2024). 10.1371/journal.pcbi.1012164 PMC11164360 38809952

[100] Abdennur, N. & Mirny, L. A. Cooler: scalable storage for Hi-C data and other genomically labeled arrays. *Bioinformatics* **36**, 311–316 (2020). 31290943 10.1093/bioinformatics/btz540 PMC8205516

[101] Imakaev, M. et al. Iterative correction of Hi-C data reveals hallmarks of chromosome organization. *Nat. Methods* **9**, 999–1003 (2012). 22941365 10.1038/nmeth.2148 PMC3816492

[102] Knight, P. A. & Ruiz, D. A fast algorithm for matrix balancing. *IMA J. Numer. Anal.* **33**, 1029–1047 (2013).

[103] Open2C et al. Cooltools: enabling high-resolution Hi-C analysis in Python. *PLOS Comput. Biol.* **20**, e1012067 (2024) 10.1371/journal.pcbi.1012067 PMC11098495 38709825

[104] Xu, W. et al. CoolBox: a flexible toolkit for visual analysis of genomics data. *BMC Bioinform.* **22**, 489 (2021). 10.1186/s12859-021-04408-w PMC8504052 34629071

[105] Kerpedjiev, P. et al. HiGlass: web-based visual exploration and analysis of genome interaction maps. *Genome Biol.* **19**, 125 (2018). 30143029 10.1186/s13059-018-1486-1 PMC6109259

[106] Lin, D., Sanders, J. & Noble, W. S. HiCRep.py: fast comparison of Hi-C contact matrices in Python. *Bioinformatics* **37**, 2996–2997 (2021). 33576390 10.1093/bioinformatics/btab097 PMC8479650

[107] Vilarrasa-Blasi, R. et al. Dynamics of genome architecture and chromatin function during human B cell differentiation and neoplastic transformation. *Nat. Commun.* **12**, 651 (2021). 33510161 10.1038/s41467-020-20849-y PMC7844026

[108] Flyamer, I. M., Illingworth, R. S. & Bickmore, W. A. Coolpup.py: versatile pile-up analysis of Hi-C data. *Bioinformatics* **36**, 2980–2985 (2020). 32003791 10.1093/bioinformatics/btaa073 PMC7214034

[109] Rowley, M. J. et al. Analysis of Hi-C data using SIP effectively identifies loops in organisms from *C. elegans* to mammals. *Genome Res.*10.1101/gr.257832.119 (2020). 10.1101/gr.257832.119 PMC7111518 32127418

[110] Mota-Gómez, I. et al. Sex-determining 3D regulatory hubs revealed by genome spatial auto-correlation analysis. Preprint at *bioRxiv*10.1101/2022.11.18.516861 (2022).

[111] Kamada, T. & Kawai, S. An algorithm for drawing general undirected graphs. *Inf. Process. Lett.* **31**, 7–15 (1989).

[112] Moran, P. A. P. Notes on continuous stochastic phenomena. *Biometrika* **37**, 17–23 (1950). 15420245

[113] Anselin, L. Local indicators of spatial association—LISA. *Geogr. Anal.* **27**, 93–115 (1995).

[114] Heinz, S. et al. Simple combinations of lineage-determining transcription factors prime cis-regulatory elements required for macrophage and B cell identities. *Mol. Cell* **38**, 576–589 (2010). 20513432 10.1016/j.molcel.2010.05.004 PMC2898526

[115] Machlab, D. et al. monaLisa: an R/Bioconductor package for identifying regulatory motifs. *Bioinformatics* **38**, 2624–2625 (2022). 35199152 10.1093/bioinformatics/btac102 PMC9048699

[116] Armstrong, J. et al. Progressive Cactus is a multiple-genome aligner for the thousand-genome era. *Nature* **587**, 246–251 (2020). 33177663 10.1038/s41586-020-2871-y PMC7673649

[117] Hickey, G., Paten, B., Earl, D., Zerbino, D. & Haussler, D. HAL: a hierarchical format for storing and analyzing multiple genome alignments. *Bioinformatics* **29**, 1341–1342 (2013). 23505295 10.1093/bioinformatics/btt128 PMC3654707

[118] Hubisz, M. J., Pollard, K. S. & Siepel, A. PHAST and RPHAST: phylogenetic analysis with space/time models. *Brief. Bioinform.* **12**, 41–51 (2011). 21278375 10.1093/bib/bbq072 PMC3030812

[119] Pollard, K. S., Hubisz, M. J., Rosenbloom, K. R. & Siepel, A. Detection of nonneutral substitution rates on mammalian phylogenies. *Genome Res.* **20**, 110–121 (2010). 19858363 10.1101/gr.097857.109 PMC2798823

[120] Derelle, R., Philippe, H. & Colbourne, J. K. Broccoli: combining phylogenetic and network analyses for orthology assignment. *Mol. Biol. Evol.* **37**, 3389–3396 (2020). 32602888 10.1093/molbev/msaa159

[121] Minh, B. Q. et al. IQ-TREE 2: new models and efficient methods for phylogenetic inference in the genomic era. *Mol. Biol. Evol.* **37**, 1530–1534 (2020). 32011700 10.1093/molbev/msaa015 PMC7182206

[122] Gel, B. et al. regioneR: an R/Bioconductor package for the association analysis of genomic regions based on permutation tests. *Bioinformatics* **32**, 289–291 (2016). 26424858 10.1093/bioinformatics/btv562 PMC4708104

[123] Najle, S. R. et al. Stepwise emergence of the neuronal gene expression program in early animal evolution. *Cell* **186**, 4676–4693 (2023). 37729907 10.1016/j.cell.2023.08.027 PMC10580291

[124] Koromila, T. et al. Odd-paired is a pioneer-like factor that coordinates with Zelda to control gene expression in embryos. *eLife* **9**, e59610 (2020). 32701060 10.7554/eLife.59610 PMC7417190

[125] Akgol Oksuz, B. et al. Systematic evaluation of chromosome conformation capture assays. *Nat. Methods* **18**, 1046–1055 (2021). 34480151 10.1038/s41592-021-01248-7 PMC8446342

[126] Bolger, A. M., Lohse, M. & Usadel, B. Trimmomatic: a flexible trimmer for Illumina sequence data. *Bioinformatics* **30**, 2114–2120 (2014). 24695404 10.1093/bioinformatics/btu170 PMC4103590

[127] Zhang, Y. et al. Model-based analysis of ChIP–seq (MACS). *Genome Biol.* **9**, R137 (2008). 18798982 10.1186/gb-2008-9-9-r137 PMC2592715

[128] Bentsen, M. et al. ATAC-seq footprinting unravels kinetics of transcription factor binding during zygotic genome activation. *Nat. Commun.* **11**, 4267 (2020). 32848148 10.1038/s41467-020-18035-1 PMC7449963

[129] Li, X.-Y., Harrison, M. M., Villalta, J. E., Kaplan, T. & Eisen, M. B. Establishment of regions of genomic activity during the *Drosophila* maternal to zygotic transition. *eLife* **3**, e03737 (2014). 25313869 10.7554/eLife.03737 PMC4358338

[130] Rajagopal, N. et al. RFECS: a random-forest based algorithm for enhancer identification from chromatin state. *PLoS Comput. Biol.* **9**, e1002968 (2013). 23526891 10.1371/journal.pcbi.1002968 PMC3597546

[131] Ramírez, F. et al. deepTools2: a next generation web server for deep-sequencing data analysis. *Nucleic Acids Res.* **44**, 160–165 (2016). 10.1093/nar/gkw257 PMC4987876 27079975

[132] Zhang, P. et al. On the origin and evolution of RNA editing in metazoans. *Cell Rep.* **42**, 112112 (2023). 36795564 10.1016/j.celrep.2023.112112 PMC9989829

[133] Dunham, I. et al. An integrated encyclopedia of DNA elements in the human genome. *Nature* **489**, 57–74 (2012). 22955616 10.1038/nature11247 PMC3439153

[134] Dobin, A. et al. STAR: ultrafast universal RNA-seq aligner. *Bioinformatics* **29**, 15–21 (2013). 23104886 10.1093/bioinformatics/bts635 PMC3530905

[135] Sebé-Pedrós, A. et al. Cnidarian cell type diversity and regulation revealed by whole-organism single-cell RNA-seq. *Cell* **173**, 1520–1534.e20 (2018). 29856957 10.1016/j.cell.2018.05.019

[136] Zolotarov, G., Grau-Bové, X. & Sebé-Pedrós, A. GeneExt: a gene model extension tool for enhanced single-cell RNA-seq analysis. Preprint at *bioRxiv*10.1101/2023.12.05.570120 (2023). 10.1093/bioinformatics/btag094 PMC12970594 41769841

[137] Diesh, C. et al. JBrowse 2: a modular genome browser with views of synteny and structural variation. *Genome Biol.* **24**, 74 (2023). 37069644 10.1186/s13059-023-02914-z PMC10108523

[138] Hoencamp, C. et al. 3D genomics across the tree of life identifies condensin II as a determinant of architecture type. *Science* **372**, 984–989 (2021). 34045355 10.1126/science.abe2218 PMC8172041

[139] Wike, C. L. et al. Chromatin architecture transitions from zebrafish sperm through early embryogenesis. *Genome Res.* **31**, 981–994 (2021). 34006569 10.1101/gr.269860.120 PMC8168589

[140] Guo, Y. et al. Chromatin jets define the properties of cohesin-driven in vivo loop extrusion. *Mol. Cell* **82**, 3769–3780 (2022). 36182691 10.1016/j.molcel.2022.09.003

[141] Isiaka, B. N. et al. Cohesin forms fountains at active enhancers in *C. elegans*. Preprint at *bioRxiv*10.1101/2023.07.14.549011 (2023).

