# Supplementary material for: Chromatin loops are an ancestral hallmark of the animal regulatory genome
Source: Nature. 2025 May 7;642(8069):1097–105. doi: 10.1038/s41586-025-08960-w (PMC12221973; doi:10.1038/s41586-025-08960-w)
Supplement: Supplementary file 1 — Detailed Micro-C protocol used in this study. [file 41586_2025_8960_MOESM1_ESM.pdf]

nature portfolio

https://doi.org/10.1038/s41586-025-08960-w

Supplementary information

Chromatin loops are an ancestral hallmark of the animal regulatory genome

In the format provided by the authors and unedited

Micro-C protocol

Day 1

1. **Cell lysis and chromatin fragmentation**

   1.1 Resuspend cells (2x10^6 cells) in 500 μL of ice-cold MB1. Incubate on ice for 20 min. Triturate the sample up and down every two minutes or use mild vortexin. Keep the cells on ice throughout the procedure.
   MB1: 10 mM Tris-HCl (pH 7.4), 50 mM NaCl, 5 mM $MgCl_2$, 1 mM $CaCl_2$, 0.2% NP-40, 1xPIC (Roche, 11836170001)

   1.2 Pellet permeabilized cells and nuclei at 4,500 x g for 5 min at 4 °C. Discard supernatant.

   1.3 Wash cells with 500 μL of ice-cold MB1 buffer. Centrifuge at 4,500 x g for 5 min at 4 °C. Discard supernatant.

   1.4 Resuspend the pellet in 100 μL of MB1 buffer.

   1.5 Add the appropriate amount of MNase (Takara Bio, 2910a) to digest chromatin to 80% monomer / 20% dimer-oligomer nucleosome ratio.

   1.6 Incubate for 10 min at 37 °C at 850 rpm.

   1.7 Add 0.8 μL of 500 mM EGTA (pH 8.0) (to a final 4 mM) to stop the digestion reaction.
   QC1: save 7 ul of the reaction to estimate the efficiency of MNase digestion. To reverse crosslink chromatin and isolate digested DNA, adjust the volume to 100 μL and proceed to step 6.

   1.8 Incubate the sample for 10 min at 65 °C without agitation to fully inactivate the enzyme.

   1.9 Pellet cells at 4,500 x g for 5 min at 4 °C. Discard supernatant.

   1.10 Wash the pellet twice with 500 μL of ice-cold MB2 at 4,500 x g for 5 min at 4 °C.
   MB2: 10 mM Tris-HCl (pH 7.4), 50 mM NaCl, 10 mM $MgCl_2$, 0.1 % BSA

2. **Repair fragment ends after MNase treatment**

   Resuspend nuclei in 45 μL of the following reaction master mix:

	Amount, μL
Cells and nuclei from step 1	pellet
10x NEBuffer 2.1	5
nuclease-free H_2O	34
100 mM ATP	1
100 mM DTT	2.5
10 U/μL T4 PNK (NEB, M0201)	2.5
Incubate for 15 min at 37 °C with interval mixing at 850 rpm.<br>To generate 3’- 5’ single-stranded overhangs, add to the sample	
5 U/μL Klenow Fragment (NEB, M0210)	5
Incubate for another 15 min at 37 °C with interval mixing at 850 rpm.	

3. **Fill-in overhangs with biotin-labeled dNTPs**

Add to the reaction 25 μL of fill-in master mix to a total volume of 75 μL:

	Amount, μL
Reaction mixture from step 2	50
10x T4 DNA Ligase Buffer	2.5
nuclease-free H_2O	11.875
1 mM Biotin-dATP (Jena Bioscience, NU-835-BIO14)	5
1 mM Biotin-dCTP (Jena Bioscience, NU-809-BIOX)	5
10 mM dTTP + dGTP	0.5
20 mg/mL BSA	0.125

- Incubate the reaction mixture for 45 min at 25 °C with interval mixing at 850 rpm.
- Add 4.5 μL of 500 mM EDTA (pH 8.0) (to a final 30 mM). Incubate at 65 °C for 20 min without agitation. At this stage, all cells and nuclei are lysed. Chromatin is released into solution. If you are working with protists that have a cell wall, check the release of chromatin under a microscope. If chromatin is not released into solution, use bead beating.
- Add to the sample ice-cold 500 μL of MB3 buffer. Spin the chromatin for at 16,000 x g for 10 min at 4 °C. Remove supernatant.
  MB3: 50 mM Tris-HCl (pH 7.5), 10 mM $MgCl_2$
- Add another 500 μL of MB3 buffer and repeat the wash step. Spin the chromatin at 16,000 x g for 10 min at 4 °C. Discard supernatant.

4. **Proximity ligation**

Resuspend chromatin in 1.2 mL of the following reaction mixture (for 1M cells and genomes less than 1,000 Mb. If the genome is larger than 1,000 Mb, you can consider doubling the volume of a ligation reaction):

	Amount, μL
Chromatin	pellet
10X T4 DNA Ligase buffer	120
nuclease-free H_2O	920
10 % Triton X-100	100
20 mg/mL BSA	12
50 % PEG 4000	36
5 U/μL T4 DNA ligase (Thermo Scientific, EL0012)	12

- Incubate for >2.5 hours at room temperature with slow rotation.
- Spin the chromatin at 16,000 x g for 10 min at 4 °C. Discard supernatant.

5. **Remove biotin from unligated ends**

	Amount, μL
Chromatin	pellet
10x NEBuffer 1	10
nuclease-free H_2O	87
10 mg/ml RnaseA (Roche, Merck 10109142001)	1
Incubate for 10 min at 37 °C with interval mixing at 850 rpm. Add to the sample:	
100 U/μL Exonuclease III (NEB, M0206)	2
Incubate for another 5 min at 37 °C with interval mixing at 850 rpm.	

6. **Reverse crosslinking**
Add the following mixture:

	Amount, μL
Reaction mixture from step 5	100
20 mg/ml Proteinase K (Roche, 3115879001)	6
10 % SDS	12
5 M NaCl	8.5

- Incubate at 65 °C overnight with mixing at 850 rpm.
  QC1 digestion sample control incubate for at least 1 hour and proceed to DNA purification.

Day 2

7. **DNA purification**

7.1 Use DNA Clean & Concentrator-5 kit (Zymo Research, D4014) to purify de-crosslinked deproteinized DNA.

7.2 Elute isolated DNA in 33 μL of 10 mM Tris-HCl (pH 8.0).

7.3 Quantify the amount of eluted DNA with a Qubit dsDNA Quantification Assay Kit (Invitrogen, Q32854).
QC2: Save 2 μL of ligated DNA to estimate the quality of proximity ligation step. Profile both QC1 and QC2 using High sensitivity D5000 ScreenTapes (Agilent Technologies, 5067-5592) on the Agilent 2200 TapeStation systems.

8. **Capture biotinylated chimeric DNA fragments**

8.1 Wash 20 μL of Dynabeads MyOne Streptavidin T1 (Life technologies, 65602) slurry beads per sample with 400 μL of 1 x BB buffer supplemented with 0.1 % Triton X-100.
1x BB: 5 mM Tris-HCl (pH 7.5), 0.5 mM EDTA, 1 M NaCl

8.2 Place the tube on a magnet for 1 min and discard the supernatant.

8.3 Repeat the wash step one more time.

8.4 Resuspend washed beads in 100 μL of 2x BB and add 100 μL of purified DNA sample (30 μL of eluted sample + 70 μL of 10 mM Tris-HCl (pH 8.0)).

8.5 Gently mix the solution by pipetting and incubate for 30 min at room temperature on a rotating wheel.

8.6 After incubation, place the tube on a magnet for 1 min and discard the supernatant.

8.7 Wash the beads twice with 400 μL 1x BB with 0.1 % Triton-X100 incubating the solution at 55 °C for 2 min at 850 rpm.

8.8 Wash the beads once with 10 mM Tris-HCl (pH 8.0) by resuspending the solution and then placing immediately on a magnet for 1 min.

8.9 Remove supernatant.

8.10 Resuspend the beads in 55 μL of 10 mM Tris-HCl (pH 8.0) and proceed immediately to step 9.

9. **End-repair and dA-tailing**

Transfer beads to a 0.2 ml PCR tube and add the NEBNext End repair/dA-tailing mix (NEB, E7546).

	Amount, μL
Purified DNA	55.5
NEBNext Ultra II End Prep Rxn buffer	6.5
NEBNext Ultra II End Prep Enzyme Mix	3

Place the tube in a thermocycler running the following program

Temperature	Time
20 °C	30 min
65 °C	30 min
4 °C	∞

10. **Adapter ligation**

Add directly to the PCR tube from step 9 the following components of the NEBNext Ultra II Ligation Module (NEB, E7595S) and mix by pipetting. Transfer the mixture in a 1.5 mL DNA low-binding tube:

	Amount, μL
End-repair/dA-tailing mix from step 9	65
NEBNext Ultra II Ligation Master Mix	30
NEBNext Ligation Enhancer	1
1.5 μM Y-shaped annealed adapters (for adapter concentration check the NEB manual)	2.5

10.1 Mix the sample by pipetting at least 10 times.

10.2 Incubate the mixture at 20 °C for 15 min at room temperature.

10.3 Add 400 μL of 1x BB with 0.1 % Triton-X100, incubate at 55 °C for 2 min at 850 rpm mixing. Place the tube on a magnet for 1 min and discard supernatant.

10.4 Repeat the washing step once more.

10.5 Next, wash the beads once with 10 mM Tris-HCl (pH 8.0) at room temperature, place the tube on a magnet for 1 min and aspirate supernatant.

10.6 Resuspend the beads in 20 μL of 10 mM Tris-HCl (pH 8.0).

11. **PCR amplification**

Combine the following components in a 0.2 mL PCR tube:

	Amount, μL
Streptavidin bead mixture from step 10	20
25 μM Primer i5	2.5
25 μM Primer i7	2.5
NEBNext High-Fidelity 2x PCR Master Mix (NEB, M0541)	25

Place the tube in a thermocycler with the following program:

Temperature	Time	Cycles
98°C	30”	
98°C	10”	
65°C	30”	5
72°C	30”	
4°C	∞	

Remove a tube from a thermocycler and store on ice. Proceed to qPCR quantification to determine additional required cycles.

12. **qPCR amplification**

Place the PCR tube from step 11 on a magnet and take 5 μL of partially amplified library to assemble the following reaction:

	Amount, μL
Partially-amplified library without beads	5
nuclease-free H_2O	3.85
25 μM Primer i5	0.5
25 μM Primer i7	0.5
100x SYBR Green I (Life technologies, S7563)	0.15
NEBNext High-Fidelity 2x PCR Master Mix (NEB, M0541)	5

Place the tube in a qPCR thermocycler running the following program:

Temperature	Time	Cycles
98 °C	30”	
98 °C	10”	
65 °C	30”	25
72 °C	30”	
4 °C	∞	

Calculate the number of additional PCR cycles needed for each sample using identified Ct value. Total number of cycles: 5 + (Ct – 3.17).
If Ct equals 9, then the number of cycles added will be : 9 – 3.17 = 5.83 (6). And the total number of PCR cycles will be equal: 5 + 6 = 11. Alternative method: Cycles needed = 1/3 max R – 5 cycles (amplified already).

13. **Final PCR amplification**
Continue with PCR amplification by adding required number of cycles:

Temperature	Time	Cycles
98 °C	30”	
98 °C	10”	
65 °C	30”	additional N cycles
72 °C	30”	
72 °C	2’	
4 °C	∞	

14. **AMPure XP beads size selection (350 bp and 750 bp)**

14.1 Transfer the amplified library into 1.5 mL DNA low-binding tube and add 55 μL of 10 mM Tris-HCl (pH 8.0) to adjust sample volume to 100 μL.

14.2 Mix the reaction with 50 μL (0.5x sample volume) of AMPure XP beads and incubate for 5 min at room temperature.

14.3 Place the tube in a magnetic stand for 3 min. Transfer the supernatant into a new tube and discard the beads.

14.4 To a new tube with supernatant, add additional 25 μL (up to 0.75x sample volume) of AMPure XP beads and incubate at room temperature for 10 min.

14.5 Place the tube on a magnet for 5 min.

14.6 After the incubation discard the supernatant.

14.7 Keep the tube on a magnetic stand and add 200 μL of freshly-prepared 80 % (v/v) ethanol without disturbing the beads.

14.8 Wait for 30 seconds and discard the ethanol supernatant. Repeat the previous step

14.9 Remove ethanol supernatant and leave the tube to dry until the beads are no longer shiny.

14.10 Elute prepared libraries from the beads with 15 μL of 10 mM Tris-HCl (pH 8.0). Incubate the sample at room temperature for 5 min, then place on magnet for 5 min, and collect eluted Micro-C libraries in a new DNA low-binding tube. Store purified libraries at -20 °C.

Materials and reagents:

Item	Source	Identifier
cOmplete, Mini, EDTA-free Protease Inhibitor Cocktail	Roche	11836170001
Mnase	Takara Bio	2910a
NEBuffer 2.1	NEB	B7202S
T4 PNK	NEB	M0201
DNA Polymerase I, Large (Klenow)	NEB	M0210
Biotin-14-dATP	Jena Bioscience	NU-835-BIO14
Biotin-14-dCTP	Jena Bioscience	NU-809-BIOX
100 mM ATP	Thermo Scientific	R0441
BSA	NEB	B9000S
T4 DNA ligase	Thermo Scientific	EL0012
Rnase A	Roche	10109142001
Exonuclease III	NEB	M0206
Proteinase K recombinant PCR grade	Roche	3115879001
Dynabeads MyOne Streptavidin C1	Life technologies	65001
Nebnext Ultra II End Repair/dA-Tailing Module	NEB	E7546
NEBNext Ultra II Ligation Module	NEB	E7595
NEBNext High-Fidelity 2X PCR Master Mix	NEB	M0541
SYBR Green I	Life technologies	S7563
Ampure XP reagent	Beckman Coulter	A63881
DNA Clean & Concentrator-5	Zymo Research	D4014
