# Supplementary material for: Chromatin loops are an ancestral hallmark of the animal regulatory genome
Source: Nature. 2025 May 7;642(8069):1097–105. doi: 10.1038/s41586-025-08960-w (PMC12221973; doi:10.1038/s41586-025-08960-w)
Supplement: Supplementary file 2 — Reporting Summary [file 41586_2025_8960_MOESM2_ESM.pdf]

nature portfolio

Corresponding author(s): Arnau Sebe-Pedros

Last updated by author(s): Mar 11, 2025

Reporting Summary

Nature Portfolio wishes to improve the reproducibility of the work that we publish. This form provides structure for consistency and transparency in reporting. For further information on Nature Portfolio policies, see our Editorial Policies and the Editorial Policy Checklist.

Statistics

For all statistical analyses, confirm that the following items are present in the figure legend, table legend, main text, or Methods section.

n/a	Confirmed	
☐	☒	The exact sample size (*n*) for each experimental group/condition, given as a discrete number and unit of measurement
☐	☒	A statement on whether measurements were taken from distinct samples or whether the same sample was measured repeatedly
☐	☒	The statistical test(s) used AND whether they are one- or two-sided<br>*Only common tests should be described solely by name; describe more complex techniques in the Methods section.*
☒	☐	A description of all covariates tested
☒	☐	A description of any assumptions or corrections, such as tests of normality and adjustment for multiple comparisons
☐	☒	A full description of the statistical parameters including central tendency (e.g. means) or other basic estimates (e.g. regression coefficient) AND variation (e.g. standard deviation) or associated estimates of uncertainty (e.g. confidence intervals)
☐	☒	For null hypothesis testing, the test statistic (e.g. *F*, *t*, *r*) with confidence intervals, effect sizes, degrees of freedom and *P* value noted<br>*Give P values as exact values whenever suitable.*
☒	☐	For Bayesian analysis, information on the choice of priors and Markov chain Monte Carlo settings
☒	☐	For hierarchical and complex designs, identification of the appropriate level for tests and full reporting of outcomes
☒	☐	Estimates of effect sizes (e.g. Cohen's *d*, Pearson's *r*), indicating how they were calculated

Our web collection on statistics for biologists contains articles on many of the points above.

Software and code

Policy information about availability of computer code

Data collection	Sofwtare used to collect/preprocess data in this study (package/version/source):<br>bcl2fastq 2.20 Illumina<br>Guppy 6.0.1 Oxford Nanopore<br>Guppy 5.0.17 Oxford Nanopore<br>BD FACSDiva 6.1.3 BD Biosciences<br>FlowJo v10.7 BD Biosciences<br>Megalodon 2.5 Oxford Nanopore<br>Proteome Discoverer v2.5 Thermo Fisher Scientific<br>Mascot search engine v2.6 Matrix Science
Data analysis	Open source software/packages used in for data analysis in this study (package/version/source):<br>Medaka 1.5.0 Oxford Nanopore<br>NextDenovo 2.5.0 https://github.com/Nextomics/NextDenovo<br>Flye 2.9-b1768 https://github.com/mikolmogorov/Flye<br>NECAT 0.0.1 https://github.com/xiaochuanle/NECAT<br>minimap2 2.18-r1015 https://github.com/lh3/minimap2<br>Shasta 0.8.0 https://github.com/paoloshasta/shasta<br>purge_dups 1.2.5 https://github.com/dfguan/purge_dups<br>BRAKER2 https://github.com/Gaius-Augustus/BRAKER<br>TSEBRA https://github.com/Gaius-Augustus/TSEBRA

StringTie https://github.com/gpertea/stringtie
Liftoff 1.6.1 https://github.com/agshumate/Liftoff
Broccoli 1.2 https://github.com/rderelle/Broccoli
Cactus 2.6.4 https://github.com/ComparativeGenomicsToolkit/cactus
EDTA 2.1.0 https://github.com/oushujun/EDTA
biobambam2 (https://github.com/gt1/biobambam2)
Juicer 1.6 https://github.com/aidenlab/juicer
Juicebox Assembly Tools 1.11.08 https://github.com/aidenlab/Juicebox
METALoci 0.3.0 https://github.com/3DGenomes/METALoci
pybbi 0.4.0 https://github.com/nvictus/pybbi
metacell 0.3.41 https://github.com/tanaylab/metacells
bwa 0.7.17 https://github.com/lh3/bwa
pairtools 0.2.2 https://github.com/open2c/pairtools
cooler 0.8.11 https://github.com/open2c/cooler
cooltools 0.5.1 https://github.com/open2c/cooltools
coolpuppy 1.1.0 https://github.com/open2c/coolpuppy
coolbox 0.3.8 https://github.com/GangCaoLab/CoolBox
SIP_HiC 1.6.1 https://github.com/PouletAxel/SIP
TOBIAS 0.13.3 https://github.com/loosolab/TOBIAS
MACS2 2.2.6 https://github.com/macs3-project/MACS
Phast toolkit http://compgen.cshl.edu/phast/
HOMER 4.11.1 http://homer.ucsd.edu/homer/motif/
Trimmomatic 0.39 http://www.usadellab.org/cms/?page=trimmomatic
BUSCO 5.1.2 https://busco.ezlab.org/busco_userguide.html
HISAT2 https://daehwankimlab.github.io/hisat2/
bedGraphToBigWig 2.9 UCSC
data.table 1.13.0 cran.r-project.org
topGO 2.34.0 Bioconductor
ggplot2 3.4.4 Bioconductor
ggprism 1.0.4 Bioconductor
plyr 1.8.9 Bioconductor
rtracklayer 1.62.0 Bioconductor
GenomicRanges 1.54.1 Bioconductor
GenomicFeatures 1.54.3 Bioconductor
mixtools R 2.0.0 Bioconductor
mclust 6.1.1 Bioconductor
monaLisa 1.12.0 Bioconductor

For manuscripts utilizing custom algorithms or software that are central to the research but not yet described in published literature, software must be made available to editors and reviewers. We strongly encourage code deposition in a community repository (e.g. GitHub). See the Nature Portfolio guidelines for submitting code & software for further information.

Data

Policy information about availability of data

All manuscripts must include a data availability statement. This statement should provide the following information, where applicable:

- Accession codes, unique identifiers, or web links for publicly available datasets
- A description of any restrictions on data availability
- For clinical datasets or third party data, please ensure that the statement adheres to our policy

All sequencing data is deposited in GEO under accession number GSE260572. Processed data, annotation tables, and code for reproducing the analysis are available on GitHub https://github.com/sebepedroslab/early-metazoa-3D-chromatin. All generated datasets can be explored in interactive genome browsers: https://sebelab.crg.eu/3d-genomes-arc-jb2. The chromatin proteomics data is deposited to the PRIDE repository with the dataset identifier PXD056500. The de novo sequenced genome of C. owczarzaki is deposited under BioProject PRJDB19057; M. leidyi genome: BioProject PRJNA1174117 (genome accession number JBMABS000000000); E. muelleri genome: BioProject PRJNA1175447. We used publicly available RepeatMasker (version open-4-0-3) annotation of GRCh38 genome released by UCSC https://hgdownload.soe.ucsc.edu/goldenPath/hg38/bigZips/.

Human research participants

Policy information about studies involving human research participants and Sex and Gender in Research.

Reporting on sex and gender	NA
Population characteristics	NA
Recruitment	NA
Ethics oversight	NA

Note that full information on the approval of the study protocol must also be provided in the manuscript.

Field-specific reporting

Please select the one below that is the best fit for your research. If you are not sure, read the appropriate sections before making your selection.

☒ Life sciences ☐ Behavioural & social sciences ☐ Ecological, evolutionary & environmental sciences

For a reference copy of the document with all sections, see nature.com/documents/nr-reporting-summary-flat.pdf

Life sciences study design

All studies must disclose on these points even when the disclosure is negative.

Sample size	No statistical method was used to predetermine sample size. Sample size was determined based on preliminary experiments. Multiple nuclei (n = 2,000,000 per replicate) from multiple animals were used to prepare Micro-C libraries. For ATAC-seq at least 50,000 nuclei from minimum 2 individuals were used per replicate. ChIP-seq libraries were prepared from chromatin obtained from at least 4 specimens per replicate or at least 500,000 cells.
Data exclusions	No data were excluded from the analysis.
Replication	Each Micro-C dataset is represented by at least 2 replicates. The reproducibility of replicates is confirmed by the stratum adjusted correlation coefficient. ChIP-seq and ATAC-seq datasets are represented by at least two replicates. The MARS-seq experiment was conducted using four 384-well MARS-seq plates. The DAP-seq binding assay was performed three times in independent experiments. All replication attempts were successful for all experiments.
Randomization	For each Micro-C, ChIP-seq, ATAC-seq, and RNA-seq experiment, we used more than six animals per biological replicate. Each animal was randomly selected from the laboratory culture. For unicellular organisms, at least two independent cell cultures were pooled for each biological replicate.
Blinding	Data collection and analysis was not performed blind. The experimental design of this study required direct observation, handling, and processing of morphologically distinct organisms, which prevented blinding.

Reporting for specific materials, systems and methods

We require information from authors about some types of materials, experimental systems and methods used in many studies. Here, indicate whether each material, system or method listed is relevant to your study. If you are not sure if a list item applies to your research, read the appropriate section before selecting a response.

Materials & experimental systems

n/a	Involved in the study	
☐	☒	Antibodies
☒	☐	Eukaryotic cell lines
☒	☐	Palaeontology and archaeology
☐	☒	Animals and other organisms
☒	☐	Clinical data
☒	☐	Dual use research of concern

Methods

n/a	Involved in the study	
☐	☒	ChIP-seq
☐	☒	Flow cytometry
☒	☐	MRI-based neuroimaging

Antibodies

Antibodies used	anti-H3K4me1 (Cell Signaling, 5326)<br>anti-H3K4me2 (Abcam, ab32356)<br>anti-H3K4me3 (Millipore, 07-473)<br>anti-SMC1 (ThermoFisher , A300-055A)<br>anti-H3 (Abcam, ab1791)
Validation	All antibodies used in this study are commercially available products. Their validation statements are provided on the manufacturer's websites.

Animals and other research organisms

Policy information about studies involving animals; ARRIVE guidelines recommended for reporting animal research, and Sex and Gender in Research

Laboratory animals	Sphaeroforma arctica; Salpingoeca rosetta; Capsaspora owczarzaki strain ATCC30864; Ephydatia muelleri, 7 days old specimens; Mnemiopsis leidyi, adult specimens; Hormiphora californensis, adult specimens; Trichoplax adhaerens H1 strain, adult specimens; Cladtertia collaboinventa, adult specimens; Nematostella vectensis NvElav1::mOrange transgenic line, 1.5-2 month old adult specimens.
Wild animals	No wild animals were used in this study.
Reporting on sex	The sex of the sampled animals was not recorded during collection. In unicellular holozoans, sex determination is either unreported (S. arctica, C. owczarzaki) or may represent a temporal differentiation state, where the same genotype can produce both male and female gametes (S. rosetta). Both ctenophores (M. leidyi and H. californensis) are simultaneous hermaphrodites. The sponge E. muelleri and placozoans (T. adhaerens and C. collaboinventa) do not complete their sexual cycle under laboratory conditions and reproduce asexually. N. vectensis specimens were collected before sexual maturity, making it impossible to distinguish between male and female individuals. However, we assume an equal representation of both sexes in the sample. The findings of this study reveal global genome organization principles at the whole-genome level that apply to both sexes where relevant.
Field-collected samples	No field collected samples were used in this study.
Ethics oversight	This study does not require an ethical approval.

Note that full information on the approval of the study protocol must also be provided in the manuscript.

ChIP-seq

Data deposition

☒ Confirm that both raw and final processed data have been deposited in a public database such as GEO.

☒ Confirm that you have deposited or provided access to graph files (e.g. BED files) for the called peaks.

Data access links<br>*May remain private before publication.*	The raw and processed sequencing data are deposited in GEO under accession number GSE260572.
Files in database submission	NA
Genome browser session<br>(e.g. UCSC)	https://sebelab.crg.eu/3d-genomes-arc-jb2

Methodology

Replicates	For each species, we performed at least two replicates per antibody used in this study.
Sequencing depth	15-20M reads/experiment.
Antibodies	anti-H3K4me1 (Cell Signaling, 5326)<br>anti-H3K4me2 (Abcam, ab32356)<br>anti-H3K4me3 (Millipore, 07-473)<br>anti-SMC1 (ThermoFisher , A300-055A)<br>anti-H3 (Abcam, ab1791)
Peak calling parameters	we used MACS2 2.2.6 to call peaks using the concatenated libraries for each species. Specifically, we used the callpeak utility to identify peaks from the filtered BAM files, with the following options: (i) an effective genome size equal to the ungapped genome length of each species (i.e. removing uncalled N bases), (ii) keeping duplicates from different libraries (--keep-dup all flag), (iii) retaining peaks with a false discovery rate ≤ 0.01, (iv) enabling multiple summit detection (--call-summits flag), and (v) disabling the modelling of peak extension for ChIP-seq libraries (--nomodel flag).
Data quality	FDR 5% for peak selection.
Software	macs2 version 2.2.6

Flow Cytometry

Plots

Confirm that:

☒ The axis labels state the marker and fluorochrome used (e.g. CD4-FITC).

☒ The axis scales are clearly visible. Include numbers along axes only for bottom left plot of group (a 'group' is an analysis of identical markers).

☒ All plots are contour plots with outliers or pseudocolor plots.

☒ A numerical value for number of cells or percentage (with statistics) is provided.

Methodology

Sample preparation	To sort choanocytes of E. muelleri, 7 days post hatching sponges were fed with 0.5 µm fluorescent carboxylate-modified FluoSpheres (Invitrogen, F8813). Next, animal tissue was dissociated into cells and crosslinked with formaldehyde (1%) ofr 10 min and DSG (3mM) for 40 min. Crosslinked cells were filtered through 40 µm cell strainer and stained with 2 µg/ml Hoechst 33342. For each replicate, we sorted 2,000,000 cells.<br>To sort the neuronal cell population from the N. vectensis transgenic line NvElav1::mOrange, tissue from one-day starved animals was crosslinked with 1% formaldehyde for 10 minutes under vacuum. The crosslinked tissue was then dissociated into single cells by incubating it with 10 mg/mL Protease XIV (Sigma-Aldrich, P5147) in 1/3 CMF and 1 mM $CaCl_2$ for 5 minutes at 24°C. Following digestion, the tissue was pelleted at 800 g for 5 minutes, reconstituted in 1/3 CMF supplemented with 2 mM EDTA and 2 µg/mL Hoechst 33342 (Thermo Scientific, 62249), and triturated for an additional 5–10 minutes. The crosslinked cells were passed through a 40 µm cell strainer and stained with 2 µg/mL Hoechst 33342. For each replicate, 2,000,000 cells were sorted.
Instrument	BD FACS Aria II
Software	BD FACSDiva 6.1.3 BD Biosciences
Cell population abundance	Choanocytes constituted 17-20% of total cells. Neuronal NvElav+ cell population of N. vectensis constituted 1.5-2% of total cells.
Gating strategy	For E. muelleri, cells positive for nuclei staining (Hoechst 33342) staining together with fluorescent beads (FITC channel) were sorted. For N. vectensis, we sorted cells positive for nuclei staining (Hoechst 33342) together with the fluorescent mOrange protein (PE-Texas Red channel).

☒ Tick this box to confirm that a figure exemplifying the gating strategy is provided in the Supplementary Information.
