# Supplementary figures and images for: Chromatin loops are an ancestral hallmark of the animal regulatory genome
Source: Nature. 2025 May 7;642(8069):1097–105. doi: 10.1038/s41586-025-08960-w (PMC12221973; doi:10.1038/s41586-025-08960-w)

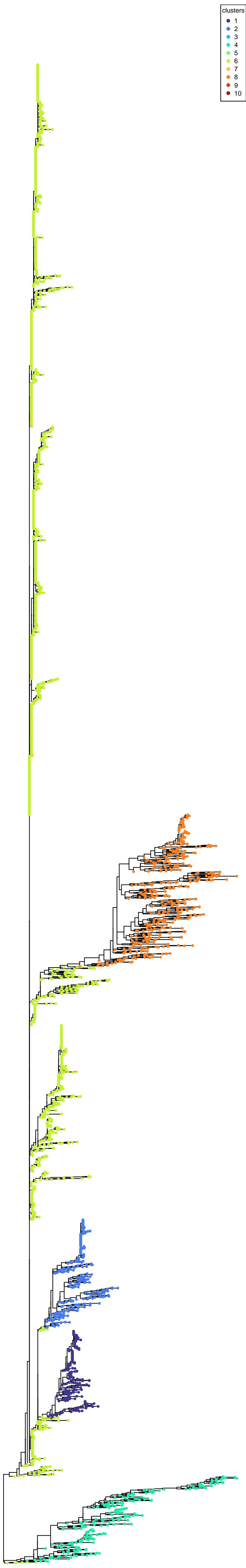

Supplement: Supplementary file 6 — Phylogenetic tree of TIR sequences of placozoan Mutator DNA transposable element. [file 41586_2025_8960_MOESM6_ESM.pdf]
